# Extracellular vesicle-mediated transmission of circPDLIM5 promotes lymphatic metastasis in prostate cancer

**DOI:** 10.1186/s13046-025-03443-2

**Published:** 2025-07-03

**Authors:** Tao He, Wen Tao, Jie Zhang, Tao-Lin Xia, Bin Xu, Liao-Yuan Li

**Affiliations:** 1 Department of Urology, Shanghai Ninth People’s Hospital, Shanghai Jiaotong University School of Medicine, 639 Zhizaoju Road, Shanghai, 200011 China; 2 Department of Urology, The First Affiliated Hospital of Guangzhou Medical University, Guangzhou, China; 3 Department of Urology, Chinese PLA General Hospital, Beijing, China; 4 Department of Health Management, Shanghai East Hospital, Tongji University School of Medicine, Shanghai, China; 5 Department of Urology, Foshan First Municipal People’s Hospital, Foshan, 528000 China

**Keywords:** Prostate cancer, Metastasis, Extracellular vesicles, Circular RNA

## Abstract

For patients with prostate cancer (PCa), pelvic lymph node (LN) metastasis remains a major poor prognostic factor associated with cancer-specific mortality. VEGF-C is a major lymphangiogenic ligand that plays a vital role in LN metastasis in PCa. However, in some PCa caseswith LN metastasis,VEGF-C is not upregulated, indicating that some VEGF-C-independent mechanisms are essential for lymphangiogenesis.Herein, we confirmed that extracellular vesicles (EVs) derived from PCa cells could promote LN metastasis in PCa independent of VEGF-C. We identified an EV circular RNA, circPDLIM5, that could promote lymphangiogenesis and lymphatic metastasis in both PCa cell lines and mouse models. Mechanistically, the packaging of circPDLIM5 into EVs was regulated by heterogeneous nuclear ribonucleoprotein A2B1. Subsequently, EVs were transmitted to human lymphatic endothelial cells, and EVs carrying circPDLIM5 could then directly interact with the transcription factor Yin Yang 1 to enhance the expression of Prospero homeobox 1, which is crucial for the formation, differentiation, and maturation of lymphatic vessels. Our findingshighlight the importance of a molecular mechanism mediated by EVs carrying circPDLIM5that is involved in lymphangiogenesis and LN metastasis in PCa; as a result, EVscarrying circPDLIM5 may be an attractive therapeutic target for LN-metastatic PCa.

**Supplementary Information:**

The online version contains supplementary material available at 10.1186/s13046-025-03443-2.

## Introduction

Prostate cancer (PCa) is the second most commonly diagnosed cancer in men, with an estimated 1.3 million diagnoses and 416,000 deaths worldwide in 2017 [1]. The majority of PCa-related deaths are associated with incurable metastatic diseases. For example, metastasis to pelvic lymph nodes (LNs) is a frequent early event and a strong predictor of poor clinical prognosis, as approximately 75% ofpatients with LN metastatic PCadevelop bone metastases within 5 years, regardless of treatment [2]. Although several clinical features of LN-metastatic PCa are known, the molecular signatures underlying the transition from a localized tumor to LN-metastatic disease are not fully understood.

Extracellular vesicles (EVs) are extracellular particles with diameters ranging from 30–150 nm that contain various bioactive molecules, such as proteins, mRNAs, microRNAs, long non-coding RNAs (lncRNAs), and circRNAs [3]. Recent studies have confirmed that EVs derived from cancer cells can act as messengers by transmitting bioactive molecules to distant organs, thereby inducing a parenchymal signaling response and remodeling the microenvironment of the metastasis site [3–5]. Indeed, EVs secreted by gastric cancer cells can deliver EGFR to hepatic stellate and Kupffer cells, thus altering the liver microenvironment and promoting liver metastasis [6]. Additionally, miR-934 in EVs derived from colorectal cancer has been shown to enhance the formation of the premetastatic niche in the liver through M2 macrophage polarization [7]. Several studies have demonstrated that tumor-derived EVs play a key role in promoting lymphangiogenesis and lymphatic metastasis across several tumor types; for example, melanoma-derived EVscontaining nerve growth factor receptor can reinforce LN pre-metastatic niche formation and metastasis [8], and cervical squamous cell carcinoma-secreted EVs-miR-221-3p can be transferred into human lymphatic endothelial cells (HLECs) to accelerate lymphatic metastasis [9]. In addition, colorectal cancer-derived EVs can remodel the lymphatic network in a sentinel LN, facilitating sentinel LN metastasis [10], and EVs containing elevated chemokine recepter-4 from highly LN-metastatic mouse hepatocarcinoma cells can promote lymphangiogenesis [11]. Recently, it has been reported that EVs derived from bladder cancer carrying LNMAT2can accelerate LN metastasis by promoting lymphangiogenesis [12].However, the underlying mechanisms of EV-induced LN metastasis in PCa remain elusive.

Circular RNA (circRNA) is a component of non-coding RNAs, and its lack of 5′ caps and 3′ poly tails endows it with unique regulatory functions during biological processes [13, 14]. Recent studies have confirmed that circRNA is associated with the progression of various cancers, including PCa [15–18]. Notably, several species of RNAs, including circRNAs, have been found inside EVs [19]. For example, an increased number of EVs carrying circMYC was detected in patients with nasopharyngeal carcinoma, and this was found to be correlated with clinicopathological variables [20]. Furthermore, PCa cells can regulate cell growth and invasion by loading EVs with high levels of circSLC19A1 [21].Despite the abundance of circRNAs in EVs, their precise function inside EVs has not yet been completely elucidated. In our previous high-throughput RNA sequencing studies of urinary EVs, we identified a novel circRNA, circPDLIM5 (circbase ID: hsa_circ_0004028), which was found to be significantly relevant to high-grade PCa [22]. CircPDLIM5 is back-spliced from six exons (exons 3 to 8) of the PDLIM5 gene (chr4:95,444,874–95,539,342) and located at the 4q22.3 amplicon. In the present study, we first confirmed that circPDLIM5 overexpression was significantly correlated with PCa LN metastasis. Subsequent in vitro and in vivo experiments showed that EVs-circPDLIM5 could promote lymphangiogenesis and LN metastasis in PCa. Mechanistically, the packaging of circPDLIM5 into EVs was found to be regulated by heterogeneous nuclear ribonucleoprotein A2B1 (hnRNPA2B1). We then transmitted EVs toHLECs, where EVs-circPDLIM5 could directly interact with transcription factor Yin Yang 1(YY1) to enhance the expression of Prospero homeobox 1 (PROX1), which is crucial for the formation, differentiation, and maturation of lymphatic vessels [12, 23]. Taken together, our results suggest that EVs-circPDLIM5 may play a vital role in accelerating lymphangiogenesis and LN metastasis of PCa independent of VEGF-C; therefore, EVs-circPDLIM5 may represent a novel therapeutic target.

## Results

### Identification of LN metastasis-associated circRNAs in PCa

**Fig. 1:**
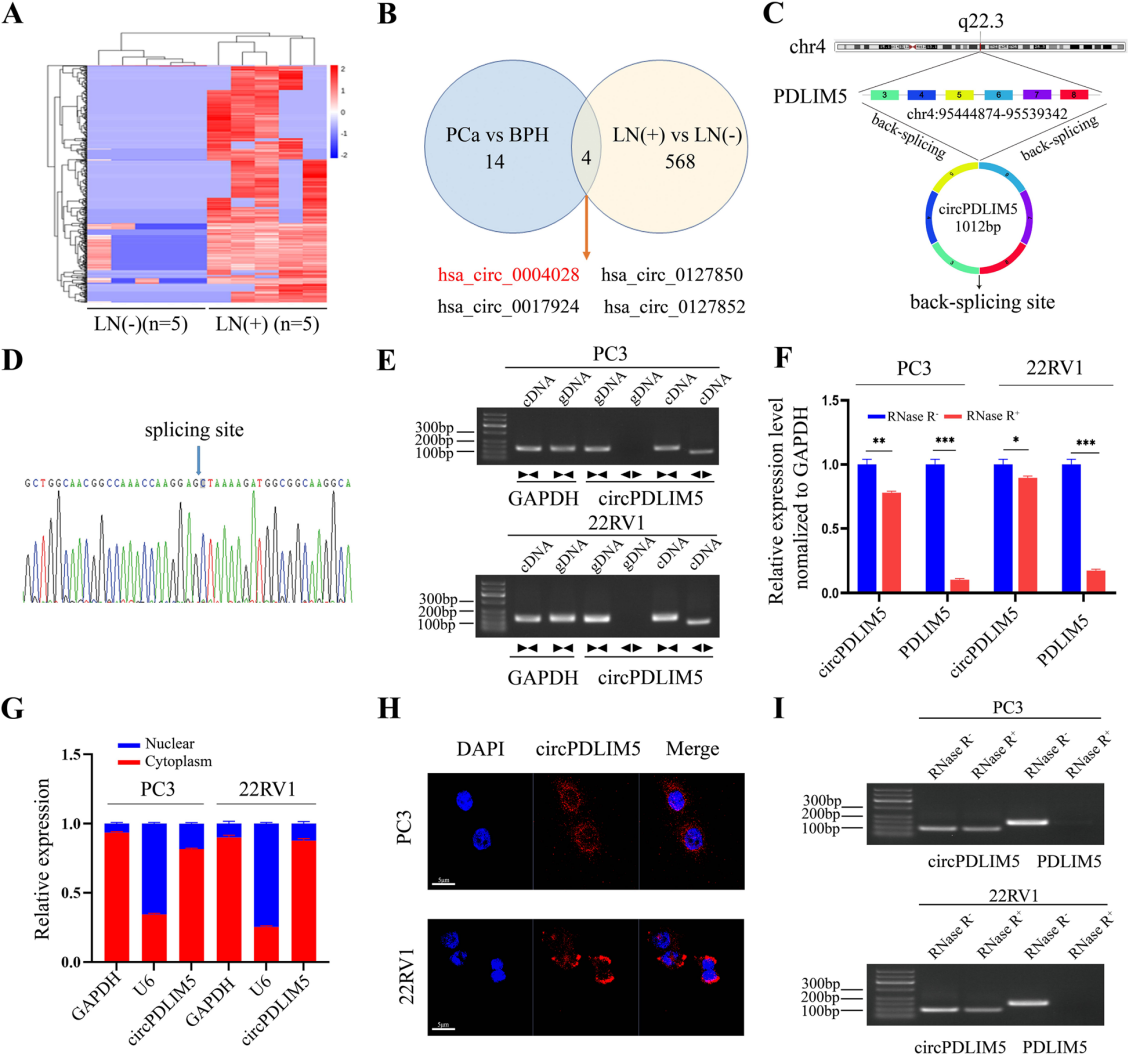
The characterization and validation of circPDLIM5 in PCa cells. **A** The cluster heat map showed the 576 differentially expressed circRNAs in 5 pairs of urinary EVs with LN metastasis and without LN metastasis. The red and blue strips indicated up-regulated and down-regulated circRNAs, respectively. **B** The overlapping analysis of up-regulated circRNAs in the urinary EVs of PCa patients compared with BPH patients and in the LN-positive patients compared with the LN-negative patients. **C** Schematic illustration revealing circPDLIM5 derived from exons 3 to 8 of PDLIM5. **D** Rolling circle reverse transcription and Sanger sequencing were used to confirm the full length of circPDLIM5 in PC3 cells. The arrow indicated the special back splicing junction site of circPDLIM5. **E** Divergent and convergent primers were used to detect circular RNAs in cDNA and gDNA, divergent primers can detect the existence of circular RNAs in cDNA but not in gDNA. GAPDH was used as a control for a linear RNA transcript (**F** and **I)**. The abundances of circPDLIM5 and PDLIM5 mRNA were determined by PCR and qRT-PCR after treatment with RNase R in PC3 and 22RV1 cells. **G** and **H** Cytoplasmic-nuclear fractionation assay and FISH assay showed that circPDLIM5 was mainly localized in the cytoplasm of PC3 and 22RV1 cells. GAPDH and U6 were used as cytoplasm control and nuclear control, respectively. Scale bars: 5 μm. Error bars represent the standard deviation (SD) of three independent experiments. *P < 0.05; **P < 0.01;***P < 0.001. Statistical significance was assessed using 2-tailed Student’s t test followed by Dunnett’s tests (**F**)

We previously used high-throughput RNA sequencing to identify 18 urinary EV circRNAs that were upregulated in high-grade PCa (GSE147761) [22]. Furthermore,to identify critical circRNAs that contribute to PCa LN metastasis, we explored the expression profiles of circRNAs in the urinary EVs of five paired LN-positive and LN-negative PCa patients, whose characteristics are summarized in Supplemental Table 1. As shown in Fig. 1 A, 572 and 4 circRNAs were upregulated and downregulated, respectively, by more than twofold in the LN-positive samples compared to the LN-negative samples. Moreover, four circRNAs, namely, hsa_circ_0004028, hsa_circ_0127850, hsa_circ_0017924, and hsa_circ_0127852, were consistently upregulated in both the high-grade PCa and LN-positive samples (Fig. 1B and Supplemental Table 2). Then, qRT-PCR was performed to analyze the expression levels of the four circRNAs in 50 LN-positive tumor tissues and paired LN-negative tumor tissues. The results showed that hsa_circ_0004028 had the highest relative expression level (Supplemental Fig. 1); thus, we selected hsa_circ_0004028 for further study.

We termed hsa_circ_0004028 as “circPDLIM5”, given that hsa_circ_0004028 is derived from PDLIM5. circPDLIM5 was derived from six exons (exons 3 to 8) of the PDLIM5 gene (chr4: 95,444,874–95,539,342) with a length of 1012 bp and located at the 4q22.3 amplicon (Fig. 1C). We then used rolling circle reverse transcription and Sanger sequencing to confirm the full-length sequence of circPDLIM5 in PC3 cells, which showed that the full-length sequence was in accordance with that reported in Circbase (Fig. 1D and Supplemental Fig. 2). To confirm the circular structure of circPDLIM5, the circPDLIM5 circular transcript and its linear counterparts were amplified by convergent and divergent primers, respectively. The gel electrophoresis results of the PCR products showed that circPDLIM5 could be detected from both primers in cDNA, while in genomic DNA (gDNA), circPDLIM5 could be detected only by the convergent primers (Fig. 1E). Additionally, the stability of circPDLIM5 was measured using an RNase R digestion assay, which demonstrated that circPDLIM5 was more resistant to RNase R digestion than its linear counterpart (Fig. 1F and I). The distribution of circPDLIM5 in PC3 and 22RV1 cells was evaluated via a cytoplasmic-nuclear fractionation assay and FISH assay, showing that circPDLIM5 was principally enriched in the cytoplasm (Fig. 1G and H). Collectively, these results showed that circPDLIM5, as a circRNA, possesses a unique circular structure and is more stable than its linear counterpart.

**Fig. 2:**
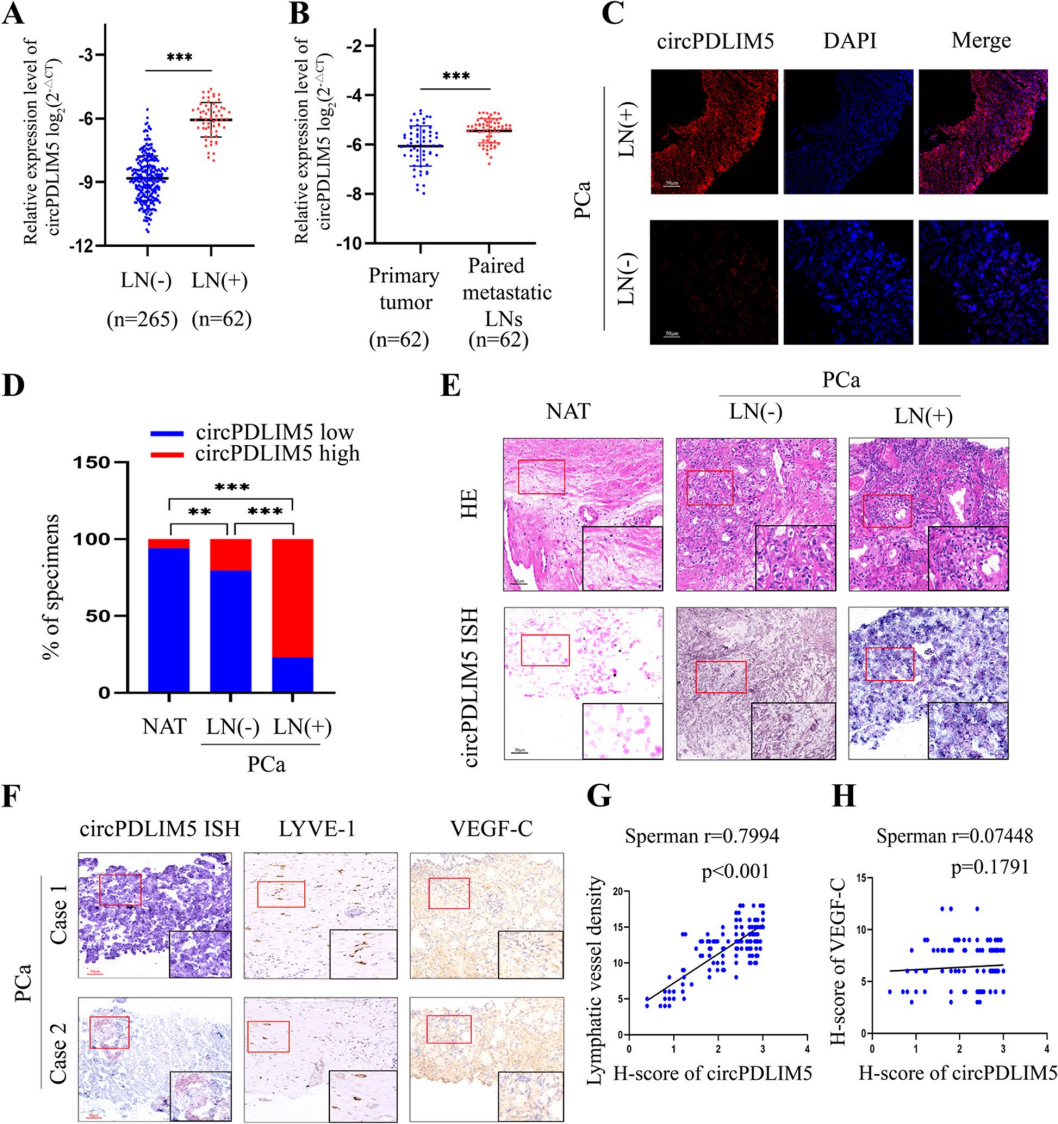
The up-regulation of circPDLIM5 is highly associated with PCa lymphatic metastasis. **A** qRT-PCR analysis of circPDLIM5 expression in PCa patients (*n* = 327) according to the LN status. **B** qRT-PCR analysis for the expression level of circPDLIM5 in the metastatic lymph nodes and paired primary PCa tissues (*n* = 62). **C** Representative images for the circPDLIM5 expression in LN(+) tissues (*n* = 62) and LN(-) tissues (*n* = 265) via FISH. Scale bars: 50 μm. **D** The percentages of circPDLIM5 expression in PCa tissues (with or without LN metastasis) and normal adjacent tissues (NAT). **E** Representative images showing circPDLIM5 expression in PCa tissues (with or without LN metastasis) and NAT by in situ hybridization (*n* = 327). Scale bars: 50 μm. The black rectangle inset is a magnification of the red rectanglearea. **F** Representative images of ISH and IHC showing the expression of circPDLIM5, LYVE-1 and VEGF-C in PCa tissues (*n* = 327). The black rectangle inset is a magnification of the red rectanglearea.The Pearson correlation analysis of circPDLIM5 and LYVE-1 (**G**), circPDLIM5 and VEGF-C (**H**) displayed that circPDLIM5 was positively correlated with lymphatic vessel density, but no correlation was observed between circPDLIM5 and VEGF-C (*n* = 327). **P < 0.01;***P < 0.001. Statistical significance was assessed using nonparametric Mann–Whitney U test (**A** and **B**), x.^2^ test (**D**)

### CircPDLIM5 overexpression correlated with PCa LN metastasis

To explore the expression profile of circPDLIM5 in PCa tissues, samples from 327 patients with PCa were investigated by qRT-PCR. The results showed that circPDLIM5 was significantly upregulated in the LN-positive tissues of patients with PCa compared to LN-negative patients (Fig. 2A and Supplemental Table 3). Similar results were obtained via FISH in PCa tissues according to LN status (Fig. 2C and Supplemental Fig. 3A). circPDLIM5 overexpression was also observed in the metastatic tumor cells in the LNs compared to the paired primary PCa tumors, indicating that circPDLIM5 is an important component of metastatic PCa cells (Fig. 2B). Consistently, circPDLIM5 was found to be significantly upregulated in the LN-positive PCa tissues compared to the LN-negative PCa tissues using an in situ hybridization (ISH) assay, whereas circPDLIM5 was rarely detected in paired normal adjacent tissues (Fig. 2D and E, and Supplemental Fig. 3B). We also found a positive correlation between the circPDLIM5 expression level and lymphatic vessel density, as indicated by the specific lymphatic vessel marker lymphatic vessel endothelial hyaluronan receptor 1 (LYVE-1) (Fig. 2F and G). Interestingly, circPDLIM5 expression was not significantly correlated with VEGF-C expression in PCa tissues (Fig. 2H).In addition, we found the expression of VEGF-C was not correlated with lymphatic vessel density (Supplemental Fig. 3C). Collectively, these results indicate that circPDLIM5 plays a key role in LN metastasis in PCa, as well asthat this process is independent of VEGF-C.

**Fig. 3:**
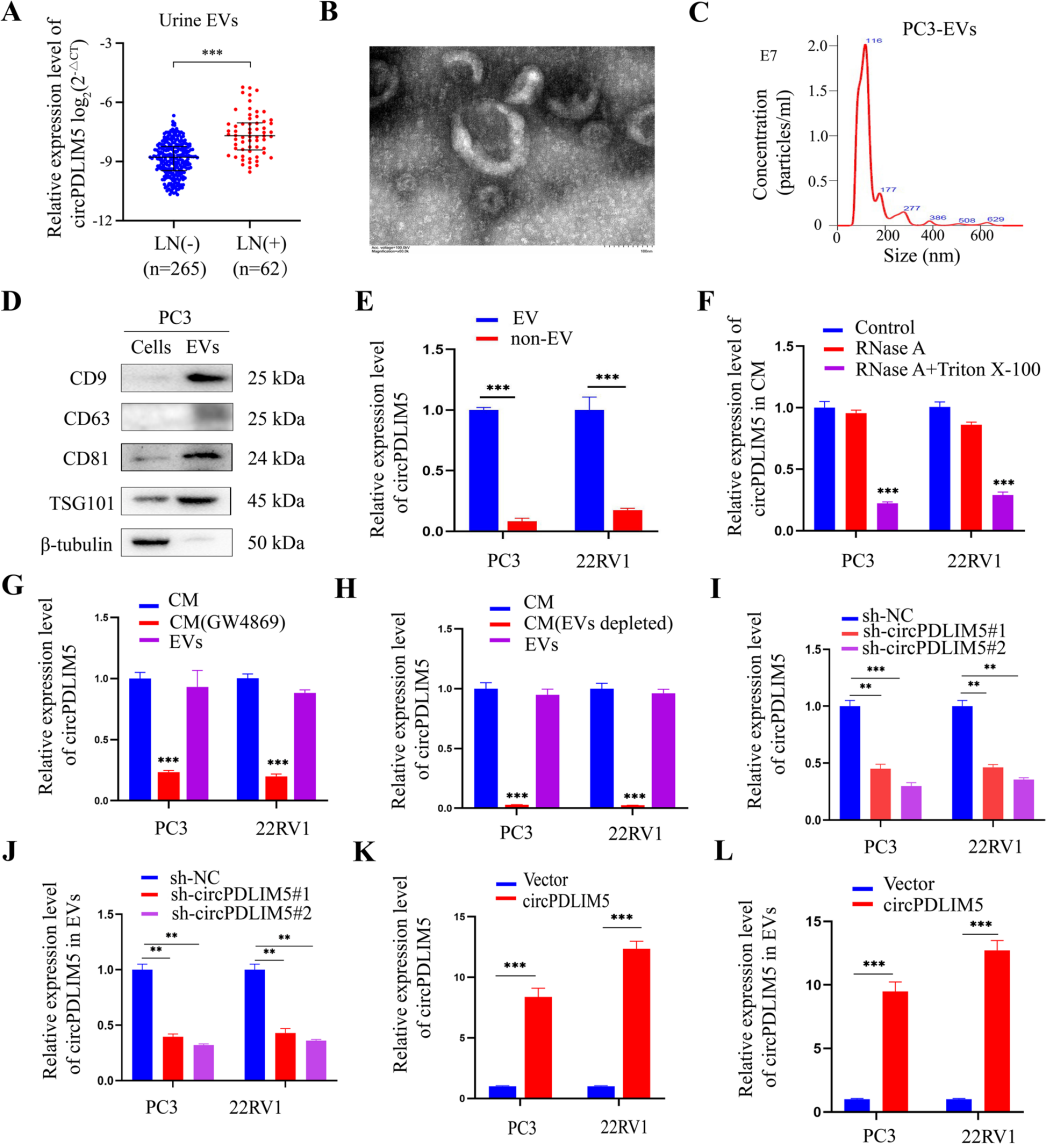
circPDLIM5 is enriched in EVs derived from PCa cells. **A** The expression level of circPDLIM5 in urinary EVs from 327 PCa patients according to the LN status. **B** and **C** TEM and NanoSight were used to identify the character of EVs purified from PC3 cells. Scale bars: 100 nm. **D** Western blot analysis of EVs-markers from PC3 EVs or cell lysates. **E** qRT-PCR analysis for the expression level of circPDLIM5 in the EV and non-EV fractions. **F** qRT-PCR analysis for the expression level of circPDLIM5 in the medium of PCa cells treated with RNase A (2 mg/mL) or in combination with Triton X-100 (0.1%) for 30 min. miR-39-3P was used as an external control. qRT-PCR analysis for the expression level of circPDLIM5 in the CM of PCa cells depleted of EVs by GW4869 (an inhibitor of EVs secretion,20 μM) (**G**) or by ultracentrifugation (**H**). miR-39-3P was used as an external control. **I** and **J** qRT-PCR analysis of circPDLIM5 expression after knockdown of circPDLIM5 in PCa cells and their corresponding EVs. **K** and **L** qRT-PCR analysis of circPDLIM5 expression after up-regulation of circPDLIM5 in PCa cells and their corresponding EVs. Error bars represent the standard deviation (SD) of three independent experiments. **P < 0.01;***P < 0.001. Statistical significance was assessed using nonparametric Mann–Whitney U test (**A**), 1-way ANOVA followed by Dunnett’s tests for multiple comparisons (**F-J**), 2-tailed Student’s t test (**E**, **K** and **L**)

### CircPDLIM5 was encapsulated in PCa cell-derived EVs

As extracellular circRNAs are mainly encapsulated in EVs, we used qRT-PCR to detect circPDLIM5 expression in the urinary EVs of 62 LN-positive and 265 LN-negative PCa patients. We found that circPDLIM5 was upregulated in the urinary EVs obtained from LN-positive PCa patients compared to those from LN-negative PCa patients (Fig. 3A).Meanwhile, increased circPDLIM5 expression was also found in the extracellular space of PCa through ISH (Fig. 2E). These results suggest that EVs- circPDLIM5 play a crucial role in LN metastasis.

We further examined circPDLIM5 expression across a panel of four PCa cell lines (PC3, 22RV1, DU145, C4-2) and human prostate epithelial cell line (RWPE-1) using a qRT-PCR assay. We found that circPDLIM5 was significantly upregulated in PCa cell lines compared to RWPE-1 (Supplemental Fig. 4A), with similar results observed in their corresponding EVs (Supplemental Fig. 4A). Interestingly, the enrichment of circPDLIM5 was detected in the EVs secreted by PCa cells, and the expression level of circPDLIM5 in EVs was similar to its expression in the PCa cells, indicating that circPDLIM5 may exert its main functions through EVs (Supplemental Fig. 4A). Subsequently, we selected two PCa cell lines (PC3 and 22RV1) with relatively high expression levels of circPDLIM5 for further study. EVs were isolated and purified from the culture medium of the two PCa cell lines and transmission electron microscopy (TEM) and NanoSight analysis were used to identify the morphology and size of the EVs, respectively. The EVs possessed a typical cup-shaped morphology and were approximately 30–150 nm in size (Fig. 3B and C, and Supplemental Figs. 4B and C). As CD9, CD63, CD81, and TSG101 serve as typical protein biomarkers for EVs [3, 4], western blotting was used to detect these biomarkers from the extracts of EVs. These experiments were used to confirm that the particles purified from the culture medium were EVs (Fig. 3D and Supplemental Fig. 4D). In addition, we found circPDLIM5 was mainly in the EVs when compared with the non-EV fractions (Fig. 3E). Notably, after treating the culture medium of PCa cells with RNase A, the expression level of circPDLIM5 changed insignificantly, while the expression level of circPDLIM5 decreased significantly after treatment with RNase A plus Triton X-100 (Fig. 3F). To further elucidate whether circPDLIM5 was mainly packaged into the EVs from PCa cells, GW4869 was used to inhibit EV secretion. The results showed that the expression level of circPDLIM5 was clearly higher in the EVs than that after treatment with GW4869, while the expression level remained unchanged compared to that in the culture medium (Fig. 3G). Additionally, qRT-PCR was used to detect the expression level of circPDLIM5 in the culture medium, EVs, and EV-depleted culture medium (purified by ultracentrifugation), showing that circPDLIM5 expression was significantly downregulated in the EV-depleted culture medium compared to that in EVs or that in total culture medium (Fig. 3H). Furthermore, we designed two shRNAs that specifically targeted the back-splice junction sites of circPDLIM5. The full length of circPDLIM5 was cloned into expression vectors to construct the overexpression vector and qRT-PCR was applied to validate the efficiency of overexpression or inhibition of circPDLIM5 in PC3 and 22RV1 cells. The results showed that both shRNAs could stably inhibit the expression of circPDLIM5, while the circPDLIM5 overexpression vector could clearly increase the expression level of circPDLIM5 (Fig. 3I and K). Similar results were observed in the EVs derived from PCa cells (Fig. 3J and L). These results suggest that the circPDLIM5 expression level in EVs was markedly influenced by that in the cell, solidifying our hypothesis that the extracellular expression of circPDLIM5 mainly exists in EVs. However, we found that the expression of circPDLIM5 can not influence the quantity of EVs (Supplemental Figs. 4E and F). Interestingly, we found that the mRNA expression of linear counterparts of circPDLIM5 was not influenced by the upregulation or downregulation of circPDLIM5 (Supplemental Figs. 4G and H). Taken together, our results indicate that the extracellular form of circPDLIM5 mainly exists in EVs.

**Fig. 4:**
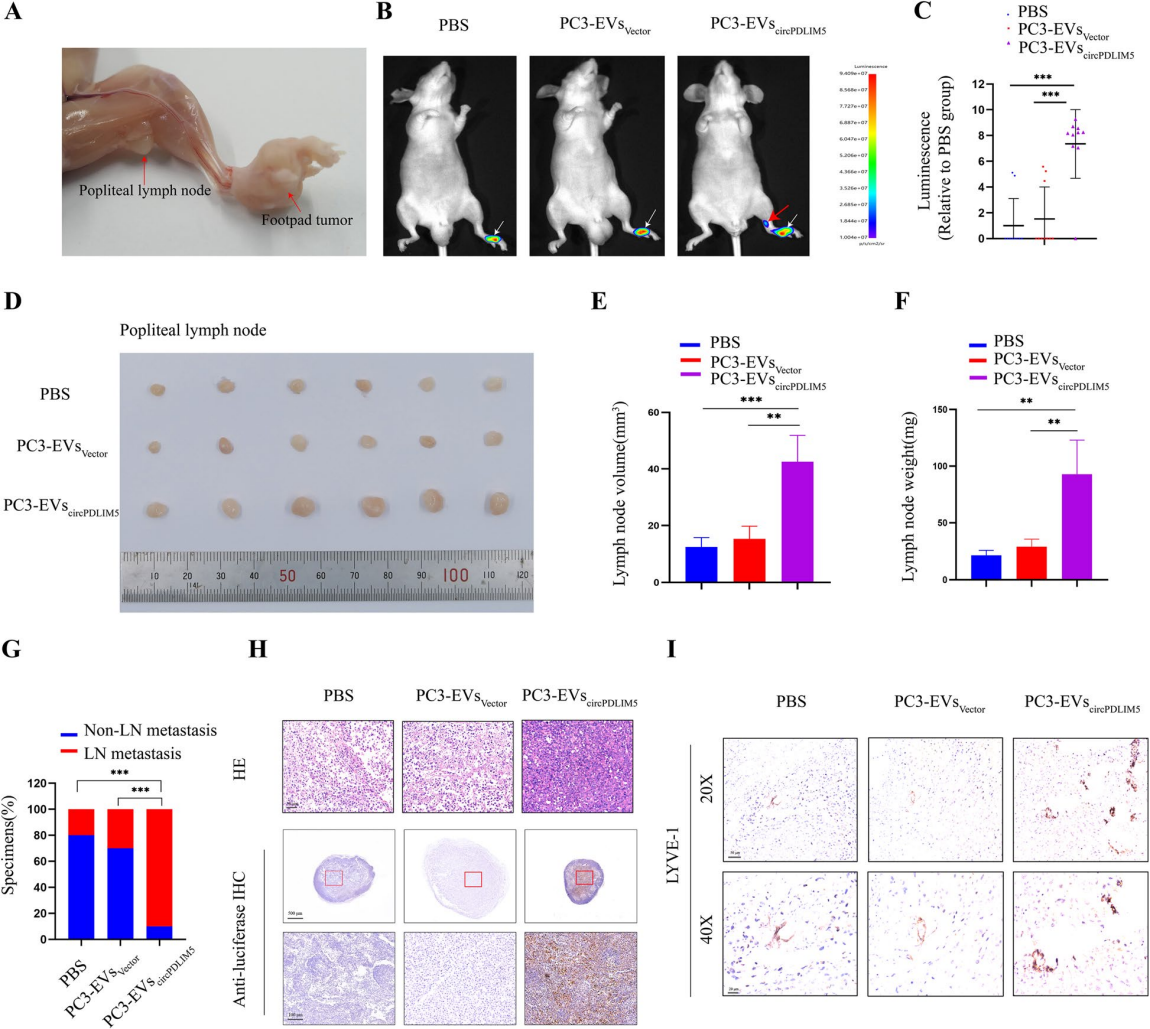
EVs-circPDLIM5 promotes lymphatic metastasis in vivo. **A** Image of the popliteal LN metastasis model in nude mice. Representative bioluminescence images (**B**) and mean photon radiance analysis (**C**) of metastatic popliteal lymph nodes from nude mice treated with PBS, PC3-EVs_Vector_, or PC3-EVs_circPDLIM5_ after inoculated with PC3 cells into the footpad (*n* = 10 per group). The white arrow represented the footpad tumor and the red arrow represented the metastatic popliteal lymph node. Representative images of dissected popliteal lymph nodes (**D**), the volume of lymph nodes (**E**), and the weight of lymph nodes (**F**) in all groups. **G** Percentages of LN metastasis in all groups (*n* = 10 per group). **H** Representative images of HE and IHC staining by anti-luciferase antibody in LNs (*n* = 10 per group). Scale bars: HE 50 μm, IHC 500 μm (up) and 100 μm (down). **I** Representative images of IHC staining by anti-LYVE-1 anti-body in footpad tumors (*n* = 10 per group).Scale bars: 50 μm (up) and 20 μm (down). *P < 0.05; **P < 0.01;***P < 0.001. Statistical significance was assessed using 1-way ANOVA followed by Dunnett’s tests (**C**, **E** and **F**), x.^2^ test (**G**)

### EVs-circPDLIM5 promoted lymphatic metastasis in vivo

To further investigate the effects of EVs-circPDLIM5 on lymphatic metastasis, we used a popliteal lymphatic metastasis model as described previously [24, 25]. First, PC3 cells that stably expressed firefly luciferase were implanted in the footpads of nude mice. Next, the mice were randomly divided into three groups (*n* = 10 per group), and subsequently received intratumoral injections of phosphate buffered saline (PBS), or of EVs secreted by vector- or circPDLIM5-transfected PC3 cells (PC3-EV_Vector_ or PC3-EV_circPDLIM5_) every 3 days (Fig. 4A). The foot-pad tumors and popliteal LNs were excised and analyzed when the primary tumor reached a size of 200 mm^3^. Interestingly, the bioluminescence signals from the IVIS System showed that PC3-EV_circPDLIM5_promoted the metastasis of PC3 cells to popliteal LNs compared to the PC3-EV_Vector_ cell or PBS group (Fig. 4B and C). Moreover, a larger popliteal LN volume and heavier popliteal LN weight were observed in the PC3-EV_circPDLIM5_ group compared to the othergroups (Fig. 4D–F). Luciferase immunostaining also showed that there was an increased LN metastasis rate in the PC3-EV_circPDLIM5_ group (Fig. 4G and H).Moreover, the expression level of LYVE-1 in murine tumor tissues was strongly correlated with EV_circPDLIM5_(Fig. 4I).Collectively, these results suggest that EV-mediated circPDLIM5 significantly facilitates the LN metastasis of PCa in vivo.

Next, to explore the tumorigenic capacity of EV-mediated circPDLIM5 in vivo, we used a subcutaneous xenograft model as previously described [15]. Briefly, PC3 cells were injected subcutaneously into the right flankof nude mice, which were then randomly divided into three groups (*n* = 6 per group). Each group received intratumoral PBS, PC3-EV_Vector_, or PC3-EV_circPDLIM5_ every 3 days for 4 consecutive weeks. After 4 weeks, there were no significant differences in tumor size or weight among these three groups (Supplemental Figs. 5A–C). Therefore, it can be concluded that EV-mediated circPDLIM5 did not play a vital role in the tumorigenicity of PCa in vivo.

**Fig. 5:**
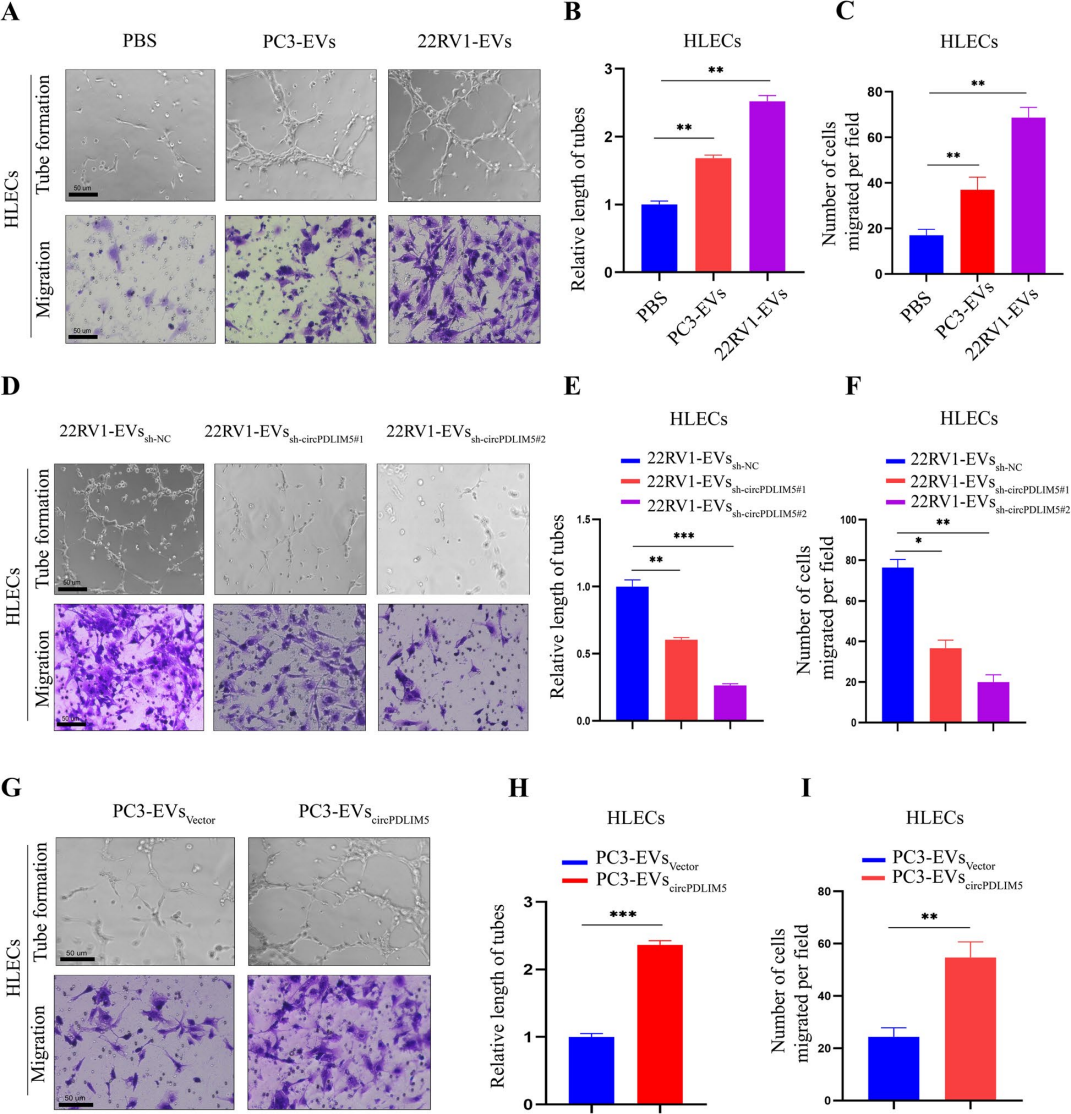
EVs-circPDLIM5 promotes lymphangiogenesis and migration of HLECs in vitro. **A-C** Representative images of HLECs cultured with PBS, PC3-EVs and 22RV1-EVs. Scale bars: 50 μm. **B** The length of the formation tubes and (**C**) the number of Transwell migration cells. **D-F** Representative images of HLECs cultured with 22RV1-EVs_sh-NC_, 22RV1-EVs_sh-circPDLIM5#1_ and 22RV1-EVs_sh-circPDLIM5#2_. Scale bars: 50 μm. **E** The length of the formation tubes and (**F**) the number of Transwell migration cells. **G-I** Representative images of HLECs cultured with PC3-EVs_Vector_ and PC3-EVs_circPDLIM5_. Scale bars: 50 μm. **H** The length of the formation tubes and (**I**) the number of Transwell migration cells. Error bars represent the standard deviation (SD) of three independent experiments. *P < 0.05; **P < 0.01;***P < 0.001. Statistical significance was assessed using 1-way ANOVA followed by Dunnett’s tests (**B**, **C**, **E** and **F**), 2-tailed Student’s t test (**H** and **I**)

### PCa cell-secreted EV circPDLIM5 promoted lymphangiogenesis in vitro

As lymphangiogenesis is a critical step for LN metastasis in PCa [26], we next investigated whether EV-mediated circPDLIM5 could facilitate lymphangiogenesis in vitro. To this end, we analyzed thetube formation and migration of HLECs incubated with EVs derived from PCa cells. Compared to the control group, the EVs secreted by PCa cells significantly promoted the tube formation and migration of HLECs (Fig. 5A–C). Furthermore, HLEC tube formation and migration were dramatically facilitated by EVs secreted by circPDLIM5-overexpressing PC3 cells (Fig. 5G–I). Conversely, circPDLIM5knockdown abolished the ability of 22RV1 cell-secreted EVs (22RV1-EV_sh-circPDLIM5_) to induce HLEC tube formation and migration (Fig. 5D–F). These results suggest that EV-mediated circPDLIM5 can induce lymphangiogenesis in vitro.

### CircPDLIM5 directly interacts with hnRNPA2B1

**Fig. 6:**
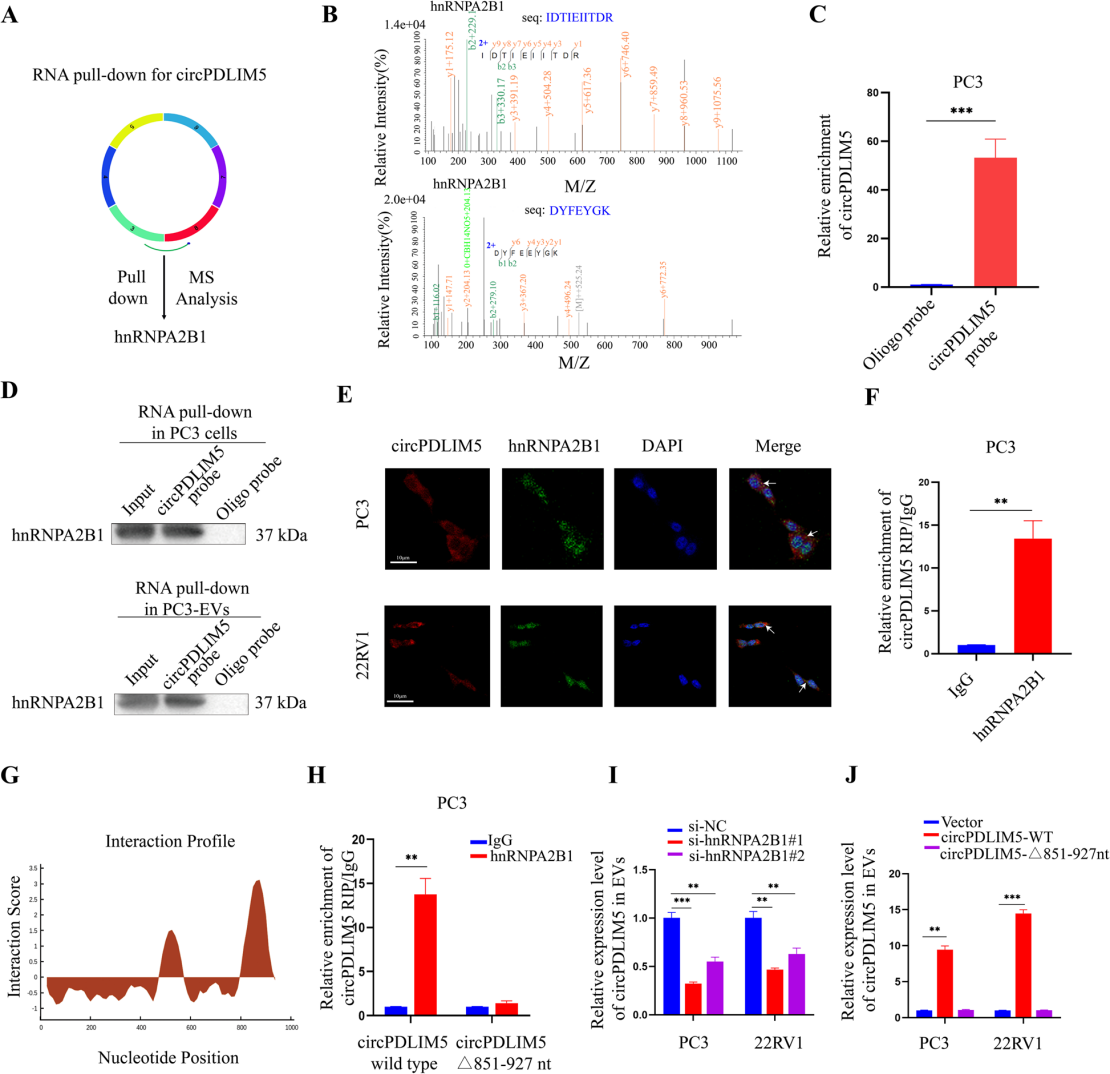
The direct interaction between circPDLIM5 and hnRNPA2B1. **A** Schematic illustration of circPDLIM5 pull-down following mass spectrometry (MS) toidentify proteins interacting with circPDLIM5. **B** Mass spectrometry analysis revealed that circPDLIM5 could interact with hnRNPA2B1. **C** qRT-PCR analysis confirmed that circPDLIM5 probe could enrich circPDLIM5 through RNA pull-down assay in PC3 cells. **D** CircPDLIM5 pull-down assay and Western blot assay in PC3 cells or in PC3-EVs validated the interaction between circPDLIM5 and hnRNPA2B1. **E** Subcellular co-localization of circPDLIM5 and hnRNPA2B1 in PCa cells measured by fluorescence staining assay. Scale bars: 10 μm. **F** The enrichment of circPDLIM5 through RIP assay by anti-hnRNPA2B1 antibody in PC3 cells. IgG was used as a negative control. **G** The binding region of circPDLIM5 to hnRNPA2B1 was predicted by catRAPID. **H** RIP assay was used to validate the binding site after mutating the 851–927 nt region of circPDLIM5 in PC3 cells. **I** qRT-PCR analysis for the expression level of circPDLIM5 in EVs when knockdown of hnRNPA2B1 in PCa cells. **J** qRT-PCR analysis of circPDLIM5 in EVs when mutating the binding sites of circPDLIM5 to hnRNPA2B1 in PCa cells.Error bars represent the standard deviation (SD) of three independent experiments. **P < 0.01;***P < 0.001. Statistical significance was assessed using 2-tailed Student’s test (**C**, **F** and **H**), 1-way ANOVA followed by Dunnett’s tests (**I** and **J**)

To investigate the molecular mechanism and interacting partners of circPDLIM5in PCa, RNA pull-down using a biotin-coupled circPDLIM5 probe was performed in circPDLIM5-overexpressing PC3 cells. Mass spectrometry (MS) analysis confirmed that hnRNPA2B1 was abundantly enriched in the circPDLIM5 probe compared to the oligo probe (Fig. 6A and B). Moreover, qRT-PCR showed that the circPDLIM5 probe could specifically enrich circPDLIM5 (Fig. 6C and Supplemental Fig. 6A). Western blotting of the RNA pull-down proteins from the circPDLIM5 probe also confirmed that circPDLIM5 could be bound to hnRNPA2B1 (Fig. 6D). Subsequently, a FISH-IF assay performed through confocal microscopy demonstrated that circPDLIM5 and hnRNPA2B1 were colocalized in the cytoplasm and nucleus of PCa cells (Fig. 6E). Additionally, an RNA immunoprecipitation (RIP) assay conducted via qRT-PCR showed that circPDLIM5 was enriched by hnRNPA2B1 in PCa cells, further confirming that hnRNPA2B1 could combine with circPDLIM5 (Fig. 6F and Supplemental Fig. 6B). To confirm which region of circPDLIM5 directly interacts with hnRNPA2B1, we first used catRAPID (http://service.tartaglialab.com/page/catrapid_group) to predict that the binding site of hnRNPA2B1 was the 851–927-nt region of circPDLIM5 (Fig. 6G). Then,serial deletion analysis was performed to validate the binding site of circPDLIM5, showing that the 850–950-nt region of circPDLIM5 was essential for combination with hnRNPA2B1 (Supplemental Fig. 6C). In addition, an RIP assay was performed after mutation of the binding site (851–927-nt region), which confirmed that this region was vital forthe interaction between circPDLIM5 and hnRNPA2B1 (Fig. 6H and Supplemental Fig. 6D).

### hnRNPA2B1 regulated circPDLIM5 packaging into EVs

As previously reported, RNAs are selectively packaged into EVs by specific RNA binding proteins, including hnRNPA2B1 [27–29]. Given that hnRNPA2B1 can bind to circPDLIM5, we next investigated whether the direct interaction between circPDLIM5 and hnRNPA2B1 could contribute to the packaging of circPDLIM5 into EVs. We found that knockdown of hnRNPA2B1 led to a decrease in circPDLIM5 in EVs, while the expression of circPDLIM5 in PCa cells was not significantly affected (Fig. 6I and Supplemental Figs. 6E and F). hnRNPA2B1 is an RNA-binding protein that can regulate the packaging of RNAs into EVs by recognizing the specific motifs GGAG/CCCU [7, 12, 27]. In particular, as we recognized the GGAG motif in the binding region (851–927 nt) of circPDLIM5, we assumed that hnRNPA2B1 may mediate the packaging of circPDLIM5 into EVs. Indeed, we observed decreased expression of circPDLIM5 in EVs when we mutated these binding sites, while the expression level of circPDLIM5 in PCa cells remained unchanged (Fig. 6J and Supplemental Fig. 6G). These results confirmed that circPDLIM5 is specifically packaged into EVs in an hnRNPA2B1-dependent manner.

### EVs-circPDLIM5 were internally absorbed by HLECs to induce lymphangiogenesis

**Fig. 7:**
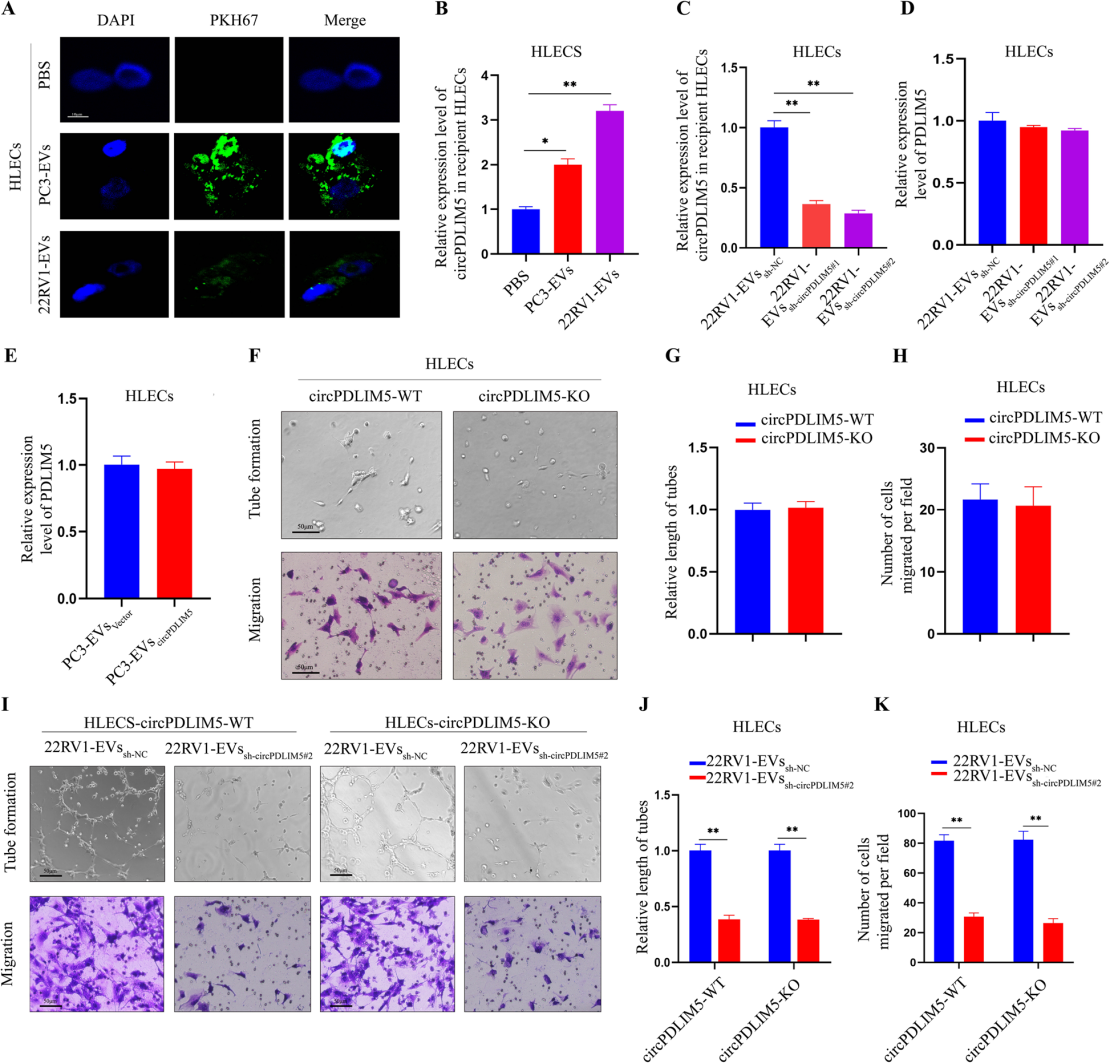
hnRNPA2B1 regulates the package of circPDLIM5 into EVs and EVs-circPDLIM5 can be absorbed by HLECs. **A** Representative images of HLECs after incubation with EVs derived from PCa cells labeled with PKH67. Scale bars: 10 μm. **B** The expression level of circPDLIM5 in HLECs when incubated with PBS, PC3-EVs and 22RV1-EVs through qRT-PCR. **C** The expression level of circPDLIM5 in HLECs when incubated with 22RV1-EVs_sh-NC_, 22RV1-EVs_sh-circPDLIM5#1_ and 22RV1-EVs_sh-circPDLIM5#2_ through qRT-PCR. qRT-PCR analysis of PDLIM5 mRNA expression in HLECs after incubated with 22RV1-EVs_sh-circPDLIM5_ (**D**) and PC3-EVs_circPDLIM5_ (**E**). **F–H** Representative images for HLECs-circPDLIM5-WT and HLECs-circPDLIM5-KO of tube formation assay and Transwell migration assay. Scale bars: 50 μm. **G** The length of the formation tubes and (**H**) the number of Transwell migration cells. **I-K** Representative images of HLECs (circPDLIM5-WT or circPDLIM5-KO) cultured with 22RV1-EVs_sh-NC_ and 22RV1-EVs_sh-circPDLIM5#2_. Scale bars: 50 μm. **J** The length of the formation tubes and (**K**) the number of Transwell migration cells. Error bars represent the standard deviation (SD) of three independent experiments. *P < 0.05; **P < 0.01. Statistical significance was assessed using 1-way ANOVA followed by Dunnett’s tests (**B-D**), 2-tailed Student’s t test (**E**,** G**,** H**,** J** and** K**)

As our previous experiments showed that EVs secreted by PCa cells could accelerate lymphangiogenesis, we next investigatedthe process by which EVs were internalized by HLECs. To this end, EVs were purified from PCa cells and labeled with PKH67 (green), then cocultured with HLECs for 12 h. The results showed that the green fluorescence signal was observed in the cytoplasm of recipient HLECs but was absent in the PBS group, indicating that HLECs could internalize the EVs secreted by PCa cells (Fig. 7A). Additionally, we found a significant increase in the amount ofcircPDLIM5 in HLECs after coculture with EVs derived from PCa cells (Fig. 7B). Furthermore, the expression of circPDLIM5 in the HLECs was decreased following incubation with EVs derived from 22RV1 cells with downregulated circPDLIM5, while the circPDLIM5 level was upregulated in HLECs when incubated with PC3-EVs_circPDLIM5_ (Fig. 7C and Supplemental Fig. 7A). However, the mRNA level of circPDLIM5 in HLECs was not influenced by incubation with 22RV1-EVs_sh-circPDLIM5_ or PC3-EVs_circPDLIM5_ (Fig. 7D and E). To investigate the mechanism of endocytosis in extracellular vesicles, we treat the culture medium of HLECs with chlorpromazine, amiloride, and nystatin after incubated with EVs derived from PCa cells. These results showed that circPDLIM5 was significantly downregulated in HLECs after being treated with amiloride, indicating that EVs were involved in endocytosis through macropinocytosis (Supplemental Fig. 7B).

To exclude the possibility that endogenous circPDLIM5 transcription in HLECs activates lymphangiogenesis, CRISPR-Cas9 was used to establish circPDLIM5-KO cells in HLECs (Supplemental Fig. 7C). Subsequently, qRT-PCR was used to determinethe depletion efficiency of circPDLIM5 in HLECs, with the results showing a goodinhibition efficiency of circPDLIM5 in HLECs (Supplemental Fig. 7D). However, the results of tube formation and Transwell assays showed that the tube formation and migration abilities of HLECs were not impacted in the circPDLIM5-KO HLECs compared to those in the circPDLIM5-WT HLECs (Fig. 7F–H).Both assays were then applied to estimate the tube formation and migration abilities of circPDLIM5-WT and circPDLIM5-KO HLECs caused by EVs-circPDLIM5. The results demonstrated that when circPDLIM5-KO HLECs were incubated with EVs derived from 22RV1 cells transfected with sh-circPDLIM5#2 (22RV1-EVs_sh-circPDLIM5_), the tube formation and migration abilities were significantly decreased compared to those of the HLECs co-cultured with EVs derived from 22RV1 cells transfected with sh-NC (22RV1-EVs_sh-NC_) (Fig. 7I–K). However, the EVs derived from PC3 cells transfected with circPDLIM5 (PC3-EVs_circPDLIM5_) promoted the tube formation and migration of circPDLIM5-KO HLECs more thanEVs derived from PC3 cells transfected with vector (PC3-EVs_Vector_) (Supplemental Figs. 7E–G). These results were consistent with those in circPDLIM5-WT HLECs (Fig. 7I–K and Supplemental Figs. 7E–G). Taken together, our results confirmed that EVs-circPDLIM5 secreted by PCa cells can induce the lymphangiogenesis of HLECs.

### EVs-circPDLIM5 promoted PROX1 expression independently of VEGF-C and VEGF-D

**Fig. 8:**
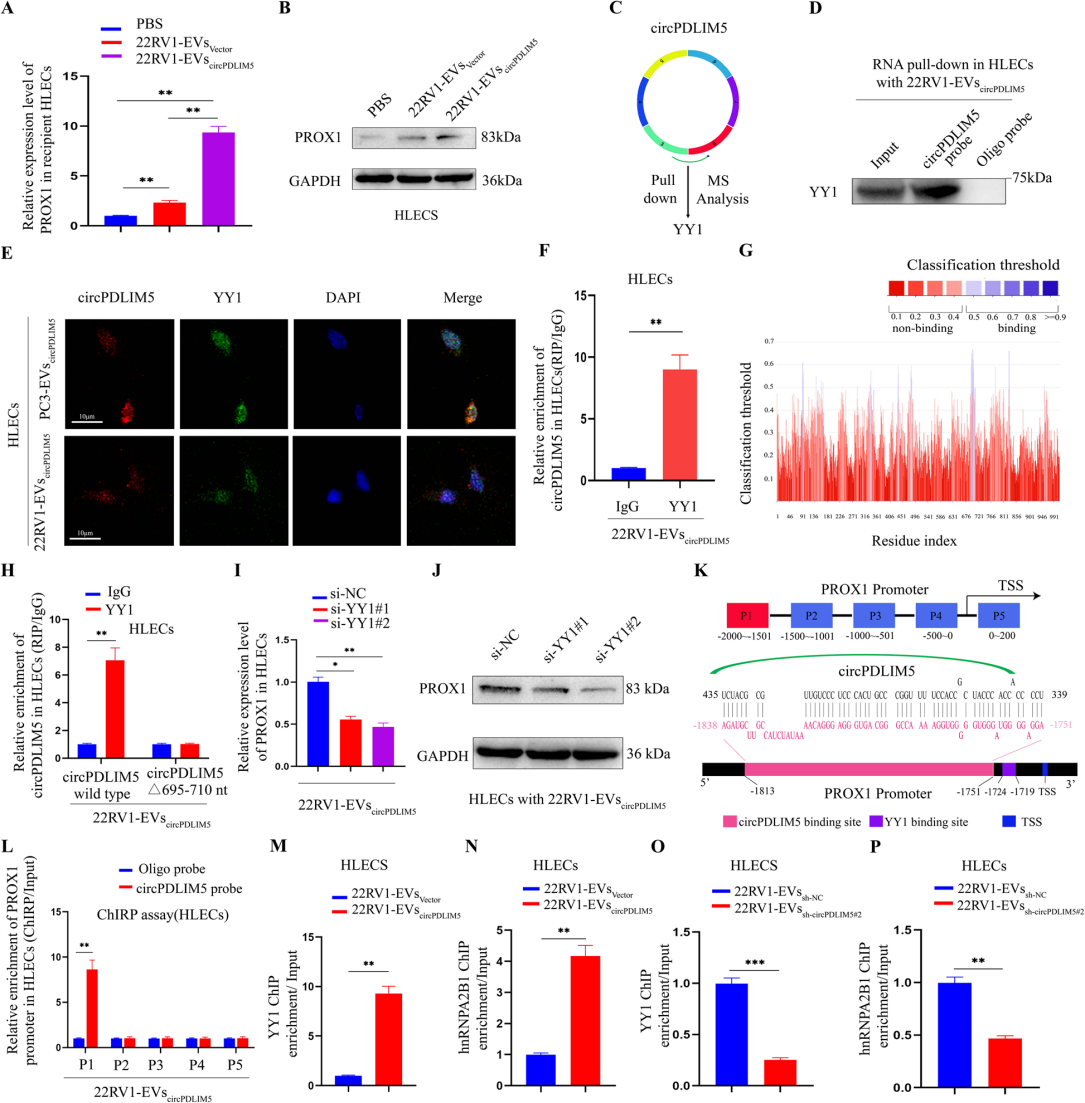
CircPDLIM5 recruits YY1 to the promoter of PROX1. qRT-PCR analysis (**A**) and Western blot assay (**B**) showed the PROX1 expression level after HLECs incubated with PBS, 22RV1-EVs_Vector_ and 22RV1-EVs_circPDLIM5_. **C** Schematic illustration of circPDLIM5 pull-down assay followed by mass spectrometry analysis in HLECs treated with 22RV1-EVs_circPDLIM5_ revealed that circPDLIM5 could interact with YY1. **D** Western blot assay for circPDLIM5 pull-down in HLECs treated with 22RV1-EVs_circPDLIM5_ confirmed the interaction between circPDLIM5 and YY1.** E** Subcellular co-localization of circPDLIM5 and YY1 in HLECs incubated with PC3-EVs_circPDLIM5_ and 22RV1-EVs_circPDLIM5_ as measured by fluorescence staining assay. Scale bars: 10 μm. **F** RIP assay through anti-YY1 antibody in HLECs cocultured with 22RV1-EVs_circPDLIM5_. IgG was used as a negative control. **G** The binding region of circPDLIM5 to YY1 was predicted by RNAInter. **H** RIP assay was used to validate the binding site of circPDLIM5 to YY1 after mutating the 695–710 nt region of circPDLIM5 in HLECs treated with 22RV1-EVs_circPDLIM5_. qRT-PCR analysis (**I**) and Western blot assay (**J**) showed the expression level of PROX1 in HLECs cocultured with 22RV1-EVs_circPDLIM5_ after knockdown of YY1. **K** Schematic illustration of the binding sites of circPDLIM5 and YY1 on PROX1 promoter and PCR-amplified fragments of the PROX1 promoter. **L** ChIRP analysis for the enrichment of PROX1 promoter fragments in HLECs incubated with 22RV1-EVs_circPDLIM5_. ChIP-qPCR of YY1 (**M** and **O**) and hnRNPA2B1 (**N** and **P**) revealed the enrichment of PROX1 promoter in HLECs incubated with relevant EVs. Error bars represent the standard deviation (SD) of three independent experiments. *P < 0.05; **P < 0.01;***P < 0.001. Statistical significance was assessed using 1-way ANOVA followed by Dunnett’s tests (**A** and **I**), 2-tailed Student’s t test (**F**,** H** and **L-P**)

VEGF-C is a major lymphangiogenic ligand that can induce the sprouting of lymphatic vessels and the proliferation of lymphatic endothelial cells [30, 31]. However, VEGF-C is not upregulated in all LN metastatic cases [32], indicating that some as yet unknown VEGF-C-independent mechanisms are essential for lymphangiogenesis. Our results showed that VEGF-C mRNA or protein remained unchanged in both circPDLIM5*-*overexpressing or circPDLIM5*-*knockdown PCa cells (PC3 and 22RV1) (Supplemental Figs. 8A–D), suggesting thatlymphangiogenesis and LN metastasisinduced by EVs-circPDLIM5in PCa might occur in a manner independent of VEGF-C. Moreover, VEGF-D,ANGPT2, FGFR3 and CXCL12 were also reported to promote lymphangiogenesis [32–34].However, we found that VEGF-D, ANGPT2, FGFR3 and CXCL12 remained unchanged in circPDLIM5-overexpressing and circPDLIM5-knockdown PCa cells through ELISA and PCR assays (Supplemental Figs. 8E-L).As PROX1 is crucial for the formation and development of lymphatic vessels [23, 35], we next applied qRT-PCR and western blotting to measure the PROX1 expression in HLECs when treated with EVs-circPDLIM5 or the control. In HLECs treated with EVs-circPDLIM5, PROX1 expression was remarkably upregulated compared to the control-treated HLECs (Fig. 8A and B, and Supplemental Figs. 8M and 8 N).

### EVs-circPDLIM5 promoted PROX1 expression by recruiting YY1 to the promoter of PROX1

To explore the underlying mechanisms by which EVs-circPDLIM5 regulate PROX1 expression in HLECs, an RNA pull-down assay through a biotin-coupled circPDLIM5 probe was performed in HLECs incubated with EVs-circPDLIM5 derived from 22RV1 cells. The results of MS analysis showed that YY1 was significantly enriched in the circPDLIM5 probe compared to that in the oligo probe (Fig. 8C and Supplemental Fig. 9A). Next, western blotting was employed to confirm that circPDLIM5 could interact with YY1 through RNA pull-down proteins from the circPDLIM5 probe in HLECs co-cultured with EVs-circPDLIM5 (Fig. 8D). Subsequently, to investigate the location of circPDLIM5 and YY1 in HLECs, a FISH-IF assay through confocal microscopy was applied in the HLECs incubated with EVs-circPDLIM5, the results of which showed that circPDLIM5 and YY1 were primarily colocalized in the nuclei of HLECs (Fig. 8E). These results suggest that circPDLIM5 delivered by EVs derived from PCa cells were transposed to the nuclei of HLECs and played vital roles. Next, a RIP assay was conducted using HLECs co-cultured with EVs-circPDLIM5, and the results showed that YY1 could accumulate circPDLIM5in abundance (Fig. 8F and Supplemental Fig. 9B). Previous studies have shown that the transcription factor YY1 is associated with tumor progression and metastasis [36–38]. Our results showed that circPDLIM5 could interact with YY1 in the nucleus of HLECs, thus we hypothesized that in HLECs, circPDLIM5 recruits YY1 to the promoter of PROX1 and sustains the expression of PROX1.

**Fig. 9:**
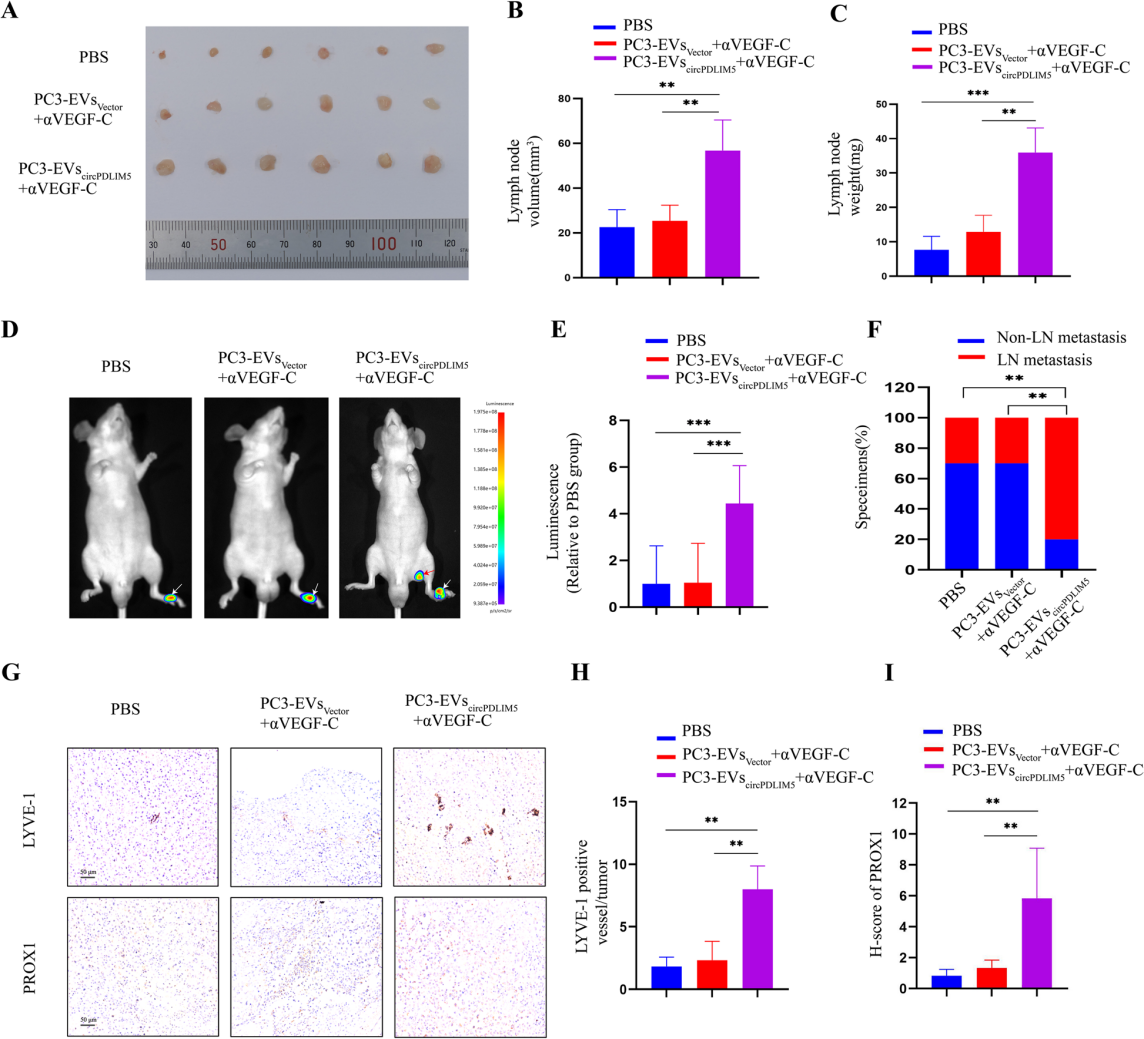
PROX1 is crucial for the EVs-circPDLIM5 induced lymphangiogenesis. **A** Representative images of dissected popliteal lymph nodes from nude mice treated with PBS, PC3-EVs_Vector_, and PC3-EVs_circPDLIM5_ with or without αVEGF-C after inoculated with PC3 cells into the footpad (*n* = 10 per group). Representative images of the volume of lymph nodes (**B**), and the weight of lymph nodes (**C**) in all groups. Representative bioluminescence images (**D**) and mean photon radiance analysis (**E**) of metastatic popliteal lymph nodes from nude mice (*n* = 10 per group). The white arrow represented the footpad tumor and the red arrow represented the metastatic popliteal lymph node. **F** Percentages of LN metastasis in all groups (*n* = 10 per group). **G** Representative images of IHC staining by anti-LYVE-1 anti-body and anti-PROX1 anti-body in footpad tumors (*n* = 10 per group). Scale bars: 50 μm. The analysis of lymphatic vessel density based on LYVE-1 (**H**) and the H-score of PROX1 (**I**) in footpad tumors (*n* = 10 per group). *P < 0.05; **P < 0.01;***P < 0.001. Statistical significance was assessed using 1-way ANOVA followed by Dunnett’s tests (**B**,** C**,** E**,** H** and **I**), x.^2^ test (**F**)

To confirm this hypothesis, bioinformatics analysis was performed using the RNAInter platform (http://www.rna-society.org/rnainter/) to explore the binding site between circPDLIM5 and YY1, with the results showing that YY1 could directly interact with circPDLIM5 in the region 695–710 nt (Fig. 8G). Interestingly, when we mutated the binding site (695–710 nt) in circPDLIM5, the RIP assay revealed that YY1 could not enrich circPDLIM5 (Fig. 8H and Supplemental Fig. 9C), confirming that this region is vital for the interaction between circPDLIM5 and YY1. Next, qRT-PCR and western blotting were conducted in HLECs co-cultured with EVs-circPDLIM5, and the results showed that PROX1 expression was significantly decreased following knockdown of YY1 (Fig. 8I and J, and Supplemental Figs. 9D and E). These results suggest that PROX1 expression may be regulated via circPDLIM5 binding with YY1.

To clarify the underlying mechanism of the upregulation of PROX1 induced by circPDLIM5 interacting with YY1, data from the RNAInter platform (http://www.rna-society.org/rnainter/) and JASPAR (http://jaspar.genereg.net/) was examined, and theyshowed that both circPDLIM5 and YY1 could directly bind to the promoter of PROX1 and that the binding sites of circPDLIM5 and YY1 are very close (Fig. 8K). Furthermore, chromatin isolation by RNA purification (ChIRP) assays confirmed that EVs-circPDLIM5 could physiologically interact with the P1 region (− 1813– − 1751 bp) of the PROX1 promoter in HLECs (Fig. 8L). Moreover, the results of the chromatin immunoprecipitation(ChIP) assay demonstrated that the PROX1 promoter sequences related to YY1 and hnRNPA2B1 could be significantly enriched in HLECs treated with EVs derived from circPDLIM5-transfected PC3 cells (PC3-EVs_circPDLIM5_) and 22RV1-EVs_circPDLIM5_ (Fig. 8M and N, and Supplemental Figs. 9F and G), while the enrichment of the PROX1 promoter sequences exhibiteda decreasing tendency in HLECs treated with PC3-EVs_sh-circPDLIM5#2_ and 22RV1-EVs_sh-circPDLIM5#2_ (Fig. 8O and P, and Supplemental Figs. 9H and I). Taken together, our results suggest that EVs-circPDLIM5 promote PROX1 expression in HLECs by recruiting YY1 to the promoter of PROX1, consequently remodeling the LN microenvironment.

### EVs-circPDLIM5 induced PROX1 upregulation promoted lymphatic metastasis

Next, we investigated the vital role of PROX1 in the process of lymphangiogenesis in PCa induced by EVs-circPDLIM5. The results showed that the tube formation and migration abilities of HLECs were reduced when incubated with EVs derived from 22RV1 cells with circPDLIM5 knockdown. Moreover, ectopic expression of PROX1 could eliminate the suppressive effects, which were independent of VEGF-C (Supplemental Figs. 10A–C). In contrast, silencing PROX1 could significantly abolish the tube formation and migration capabilities of HLECs co-cultured with PC3-EVs_circPDLIM5_ (Supplemental Figs. 10D–F). These results confirmed that PROX1 is crucial for the regulation of lymphangiogenesis and lymphatic metastasis as mediated by EVs-circPDLIM5, independent of VEGF-C.

**Fig. 10:**
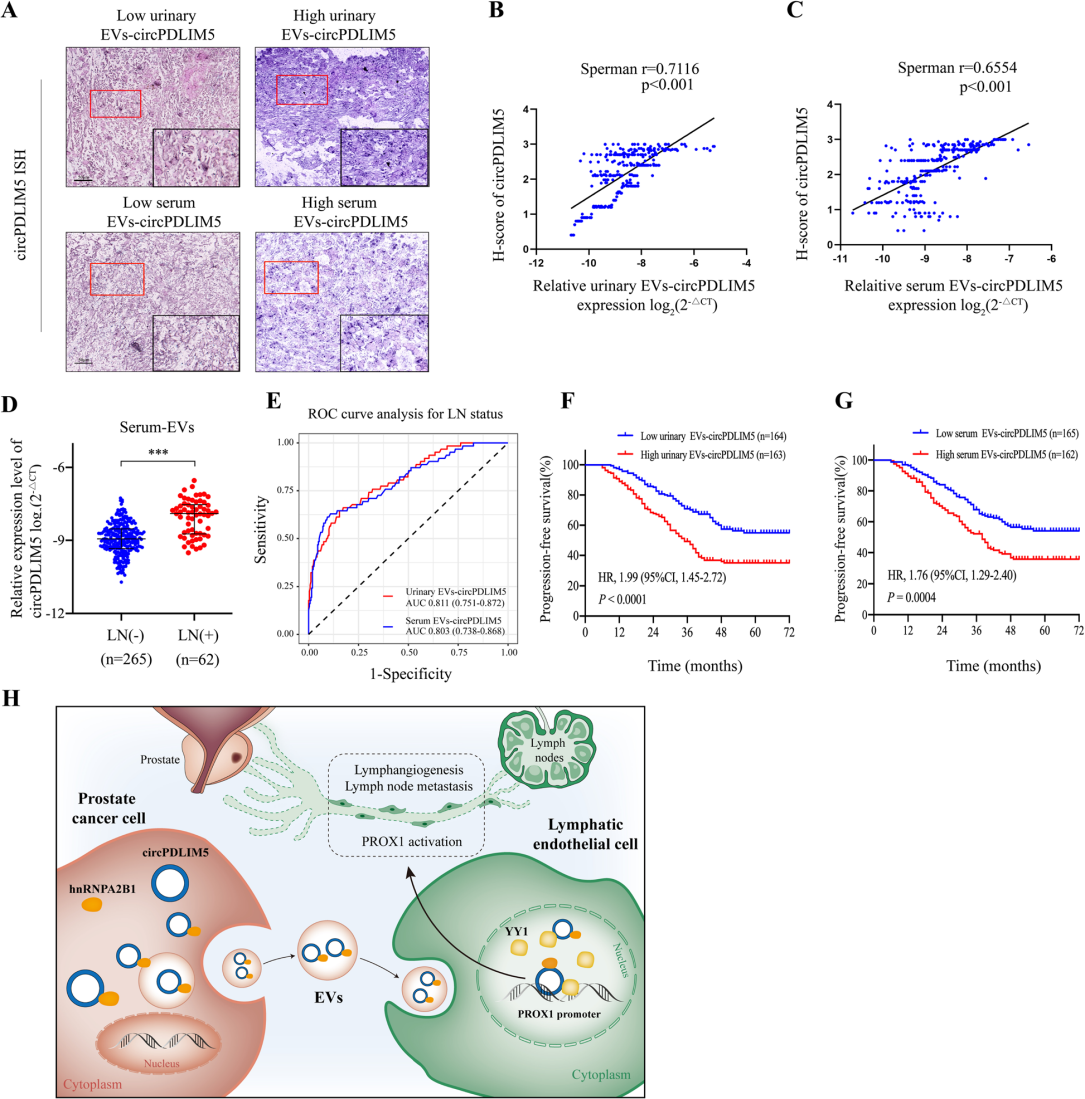
EVs-circPDLIM5 is closely related to lymphatic metastasis of PCa. **A** Representative images of circPDLIM5 expression in PCa tissues by ISH according to the expression level of circPDLIM5 in urinary EVs and serum EVs (*n* = 327). Scale bars: 50 μm. The black rectangle inset is a magnification of the red rectanglearea. **B** Correlation analysis of urinary EVs-circPDLIM5 and H-score of circPDLIM5 in tissues (*n* = 327). **C** Correlation analysis of serum EVs-circPDLIM5 and H-score of circPDLIM5 in tissues (*n* = 327). **D** qRT-PCR analysis of serum EVs-circPDLIM5 in PCa patients (*n* = 327) according to the LN status. **E** ROC curve analysis to assess the diagnostic potential of urinary EVs-circPDLIM5 and serum EVs-circPDLIM5 for PCa with LN metastasis. **F** Kaplan–Meier curves of progression-free survival according to the expression level of urinary EVs-circPDLIM5 in PCa patients (*n* = 327). **G** Kaplan–Meier curves of progression-free survival according to the expression level of serum EVs-circPDLIM5 in PCa patients (*n* = 327). **H** A schematic model showing the mechanism of PCa cell-derived EVs-circPDLIM5 promoting lymphangiogenesis and LN metastasis by up-regulation of PROX1 in HLECs. ***P < 0.001. Statistical significance was assessed using nonparametric Mann–Whitney U test (**D**)

To investigate the vital role of hnRNPA2B1 in circPDLIM5 sorting and function, tube formation assay and transwell migration assay were conducted in HLECs incubated with EVs derived from PC3 cells treated with si-NC, si-hnRNPA2B1#1 and si-hnRNPA2B1#1 + hnRNPA2B1. Our results showed that hnRNPA2B1 could partially restore the tube formation and migration capabilities of HLECs (Supplemental Figs. 11A–C). In addition, to validate the role of PROX1 in vivo, PC3 cells were implanted in the footpads of nude mice. Then, the mice were intratumoral injected with EVs-circPDLIM5. Subsequently, the mice were tail-vein injected with si-NC and si-PROX1, and we found that si-PROX1 could inhibit LN metastasis of PCa cells in mice (Supplemental Figs. 11D–F).

Furthermore, the popliteal LN metastasis model showed that the popliteal LN volume and weight were significantly larger in the PC3-EVs_circPDLIM5_ group than in the PC3-EVs_Vector_ group when both groups were treated with VEGF-C neutralizing antibody (Fig. 9A–C). Additionally, the bioluminescence signals from the IVIS System revealed that PC3-EVs_circPDLIM5_ could significantly promote the LN metastasis of PCa cells in mice relative to the PC3-EVs_Vector_ group (Fig. 9D–F). Subsequently, the expression levels of LYVE-1 in murine tumor tissues by IHC were strongly correlated with the circPDLIM5 levels, but the expression of PROX1 was low in tumor tissues (Fig. 9G–I). More importantly, we found PROX1 was highly expressed in peri-tumor tissues (Supplemental Fig. 11G and H). Our results indicate that EVs-circPDLIM5 can upregulate PROX1 expression and promote the lymphatic metastasis of PCa in a VEGF-C-independent manner.

### EVs-circPDLIM5 was closely related to lymphatic metastasis in patients with PCa

The results of the ISH assay showed that the expression of circPDLIM5 in PCa tissues was positively correlated with that in urinary and serum EVs (Fig. 10A–C). Next, we investigated the expression of circPDLIM5 in serum EVs from patients with PCa. The results showed higher expression of circPDLIM5 in the serum EVs from patients with LN metastasis compared to those without (Fig. 10D). We next evaluated the diagnostic value of urinary and serum EVs-circPDLIM5 in PCa with lymphatic metastasis. Receiver operating characteristic analysis revealed that urinary and serum EVs-circPDLIM5 could be used to discriminate between patients with PCa with LN metastasis and those without (Fig. 10E). Moreover, 71.1% (32/45) of patients with PCa incorrectly assessed as LN-negative by MRI were correctly classified as being LN-positive through the analysis of urinary EVs-circPDLIM5, showing that these EVs could be a better alternative to MRI in the detection of LN metastasis in PCa (Supplemental Table 4). In addition, Kaplan–Meier analysis showed that high levels of urinary or serumEVs-circPDLIM5 were associated with poor radiographic progression-free survival (PFS) of patients with PCa (Fig. 10F and G). Univariate and multivariate Cox proportional hazard analyses suggested that levels of urinary or serum-EVs-circPDLIM5are an independent prognostic factor for PFS in patients with PCa (Supplemental Fig. 12).Thus, our findings suggest that EVs-circPDLIM5 may be a useful biomarker and therapeutic target for PCa with LN metastases (Fig. 10H).

## Discussion

For patients with PCa, pelvic LN metastasis remains a major factorof poor prognosis associated with biochemical recurrence and cancer-specific mortality. Lymphangiogenesis, as an important mediator of cancer cell dissemination, plays a vital role in PCa LN metastasis. VEGF-C is a major lymphangiogenic ligandthat can stimulate the proliferation of lymphatic endothelial cells and the sprouting of lymphatic vessels [30, 31]. However, in some LN cases of metastatic PCa, VEGF-C is not upregulated [32], indicating that an as yet unknown VEGF-C-independent mechanism is crucial for lymphangiogenesis. In addition, numerous molecular mediators, including noncoding RNAs, adhesion molecules, cytokines, enzymes, and chemokines, contribute to lymphangiogenesis through mechanisms that either depend on or operate independently of VEGF-C/D signalling [32].Herein, we first show that a novel circRNA, circPDLIM5, is highly expressed in urinary EVs and PCa tissues. Mechanistically, EVs-circPDLIM5 promote PCa lymphatic metastasis in a VEGF-C-independent manner.Importantly, we also showed that the sorting of circPDLIM5 into EVs was regulated by hnRNPA2B1. Then, circPDLIM5 was confirmed to recruit YY1 to the PROX1 promoter to upregulate PROX1 expression in HLECs via EV internalization, ultimately promoting the lymphangiogenesis and LN metastasis of PCa. These results provide evidence of a novel VEGF-C-independent mechanismin lymphangiogenesis and LN metastasis in PCa that is mediated by EVs-circPDLIM5, indicating that circPDLIM5 is a potential therapeutic target.

Recent studies have shown that the sorting of selected RNAs into EVs is regulated by severalRNA binding proteins, although the exact mechanisms remain to be fully elucidated [27]. hnRNPA2B1, a type of RNA binding protein, has been reported to be involved in the packaging of RNA into EVs by identifying the specific motifs GGAG/CCCU of the target RNAs [7, 27]. Herein, we confirmed the presence of the GGAG motif in the binding region (851–927 nt) of circPDLIM5 and observed a decrease in circPDLIM5 expression in EVs following the mutation of these binding sites. Thus, the loading of circPDLIM5 into EVs was mediated by hnRNPA2B1, which may provide distinctive tactics for the blocking of EVs-circPDLIM5 to diminish lymphangiogenesis in PCa. Interestingly, Luo et al. reported that *KRAS* mutant-driven SUMOylation can trigger the transmission of EV-packaged heterogeneous nuclear ribonucleoprotein A1 (hnRNPA1) to promote lymphangiogenesis and LN metastasis in pancreatic ductal adenocarcinoma [39].However, our MS results showed that the enrichment of hnRNPA1 by the circPDLIM5 probe was less than that by the control probe (Sup Table 8).

Notably, we demonstrated that EVs-circPDLIM5 derived from PCa cells could significantly upregulate PROX1 expression in HLECs. PROX1 is a master regulator that acts as a fate determinator in HLECs, and it mediates numerous lymphatic-specific proteins [23, 40, 41]. PROX1 is crucial for the formation, differentiation, and maturation of lymphatic vessels, and it can enhance the proliferation of lymphatic endothelial cells [23]. Other studies have confirmed a positive relationship between PROX1 overexpression and lymphangiogenesis [42, 43]. However, the mechanisms by which PCa cell-secreted EVs-circPDLIM5 regulate PROX1 in HLECs remain elusive.

As mechanisms of action, circRNAs have been proposed to sponge microRNAs, bind directly to proteins, or act as a scaffold [44, 45]. Our results showed that circPDLIM5 could bind to YY1 in HLECs and that PROX1 was downregulated following YY1 knockdown in HLECs.Previous studies have confirmed that histone modifications are crucialin YY1-mediated gene expression. By influencing specific histone markers such as H3K27Ac and H3K4me3, YY1 can regulate enhancer-promoter interactions, thereby regulating gene expression. These histone modifications are closely related to the changes in YY1 binding sitesand play a key role in the gene regulation process [46–48].Our results also showed that the PROX1 promoter could be enriched by H3K27Ac and H3K4me3 (Supplemental Fig. 13).However, the detailed mechanism by which circPDLIM5 induced histone modifications will be further investigated.Furthermore, overexpression or silencing of PROX1 could partially reverse the prolymphangiogenesis effect induced by EVscarryingsh-circPDLIM5 or circPDLIM5, respectively. These results highlight novel mechanisms in the process of LN metastasis, which are regulated by PCa cell-derived EVs-circPDLIM5.

Another important finding of this study is that circPDLIM5 was overexpressed in both the urinaryEVs and serumEVs of patients with PCa, which was positively correlated with PCa tissues. Moreover, the high expression of circPDLIM5 was associatedwith a poor prognosis. As a type of liquid biopsy assay, the measurement of urinary or serum EVs-circPDLIM5 is repeatable, minimally invasive, and easily implemented during treatment. Additionally, owing to their biological barrier permeability, considerate biocompatibility, low toxicity, and low immunogenicity, EVs may become a promising therapeutic vehicle [49, 50]. Indeed, EVs have been shown to deliver KRAS siRNA to inhibit the growth of lung cancer [51], while the transfer of anti-miR21 oligonucleotides via modified EVs has been found to suppress tumor growth in glioblastoma [52]. In the context of PCa, accumulating evidence indicates that EVs can be bioengineered to enhance cargo encapsulation efficiency and tumor-targeting specificity. Modified EVs loaded with nucleic acids including siRNAs, mRNAs, and DNAs, as well as immune-modulating agents have demonstrated promising antitumor activity [53, 54].Therefore, the transfer of circPDLIM5 siRNA via modified EVs may represent a novel strategy for treating LN metastatic PCa.

## Conclusions

In summary, we elucidated a novel mechanism by which EVs-circPDLIM5 induced lymphangiogenesis and LN metastasis in PCa independent of VEGF-C. Our findings suggest that EVs-circPDLIM5 represent a potential noninvasive diagnostic method and therapeutic strategy for LN metastatic PCa.

## Materials and methods

### Clinical samples

A total of 327 patients with PCa who underwent radical prostatectomy and pelvic LN dissection were obtained fromFoshan First Municipal People’s Hospital.Among these patients, 62 and 265 had positive and negative LNs, respectively.We also collected preoperative serum and first-catch non-digital rectal examination urine samplesfrom all of these patients. The sample preparation, EV isolation, and characterization have all been described previously [22].

This study, which was conducted inaccordance with Declaration of Helsinki, was approved by the Institutional Ethical Review Board (SH9H-24-TK619-1), and written informed consent wasobtained from all study participants.

### Cell lines

All human PCa cell lines (PC3, 22RV1, C4-2, DU-145) and the human prostate epithelial cell line (RWPE-1) were purchased from the American Type Culture Collection (ATCC, Manassas, VA, USA). All cell lines were maintained at 37 °C with 5% CO_2_. The PC3,22RV1, and DU-145 cell lines were cultured in RPMI 1640 medium (Bioss, Beijing, China), and the C4-2 cell line was maintained in DMEM medium (Biosharp, Guangzhou, China). All media were supplemented with 10% fetal bovine serum (FBS, Gibco, USA).The RWPE-1 cell line was grown in K-SFM Basal medium, which contained both Keratinocyte-SFM (Gibco, Carlsbad, CA, USA) and BPE (Lonza, Basel, Switzerland).HLECs were purchased from ScienCell ResearchLaboratories and maintained in ECM (ScienCell ResearchLaboratories),which contained 5% FBS.

### Animal study

All animal experiments were approved by the Animal Ethics Committee of Shanghai Ninth People's Hospital.The popliteal LN metastasis model and subcutaneous tumor models were constructed using BALB/c nude mice. For the popliteal LN metastasis model, 4-week-old male BALB/c nude mice were randomly divided into aPBS group (*n* = 10), PC3-EVs_Vector_group (*n* = 10), and PC3-EVs_circPDLIM5_group (*n* = 10). Approximately 1 × 10^6^ PC3 cells were resuspended in the medium containing 20% Matrigel (BD, San Jose, CA, USA), and then injected into the footpads of each mouse. Then, each group was intratumorally injected with PBS, PC3-EVs_Vector_, and PC3-EVs_circPDLIM5_ every 3 days(100 ug of EVs). In vivo imaging was used to determine the lymphatic metastasis using the AniView100 system (BLT, Guangzhou, China). The foot-pad tumors and popliteal LNs were excised and analyzed after 4 weeks. The volume of the LN was calculated according to the formula (length × width^2^/2), and the weight of the LN was determined by electronic balance. Then, the LNs were fixed with 3.7% paraformaldehyde and embedded in paraffin, after which the sectionswere analyzed using IHC.

For the subcutaneous tumor model, 4-week-old male BALB/c nude mice were randomly divided into a PBS group (*n* = 6), PC3-EVs_Vector_group (*n* = 6), and PC3-EVs_circPDLIM5_group (*n* = 6). Approximately 2 × 10^6^ PC3 cells were resuspended in the medium containing 20% Matrigel (BD, San Jose, CA, USA), and then subcutaneously injected into the right flank of each mouse. Then, each group was intratumorally injected with PBS, PC3-EVs_Vector_, and PC3-EVs_circPDLIM5_ every 3 days(100 ug of EVs). The tumor growth was measured every week, and the tumor volume was calculated according to the formula (length × width^2^/2). All of the mice were euthanized after 4 weeks and the tumor weight was determined using anelectronic balance.

### Isolation and analysis of EVs

For EV isolation in the PCa cell medium, first, PCa cells were cultured in a medium supplemented with 10% FBS without EVs. Then, the medium was collected after culturing for 72 h at 37 °C with 5% CO_2_. In brief, the medium was centrifuged at 3,000 × g for 15 min at 4 °C to remove cells and debris. Subsequently, the supernatants were centrifuged at 10,000 × g for 70 min at 4 °C, then at 170,000 × g for 70 min at 4 °C. The pellets containing the EV were stored at − 80 °C for further use. To isolate EVs from serum, approximately8-mlwhole bloodwas collected from patients using a Vacutainer (BD, USA) and then incubated at room temperature for 30 min. The blood was then centrifuged at 1,000 × g for 10 min, after which the supernatant was centrifuged again at 2,000 × g for 20 min.The remaining supernatant was collected and purified through ultracentrifugation, as discussed previously. To isolate EVs from PCa tissues, approximately 2 g of fresh tumor tissues were kept in PBS on ice. The tissue was carefully cut into small pieces (about 2 × 2 × 2 mm) and then incubated with collagenase D and DNase I for 30 min at 37 °C under mild agitation on a nutating mixer at 24 rpm. Then, a filter with a pore size of 0.7 μm was applied to remove the largestcomponents. The remaining liquid was subsequently subjected to differential centrifugation at 300 × g for 10 min and then at 2,000 × g for 20 min toremove cells and tissue debris. Finally, thesupernatant was collected and purified through ultracentrifugation, as described previously [55]. Theprocess for isolatingEVs from urine was the same as in the medium.Next, the purification of EVs in PCa cell medium, serum,urine, and tumor tissues was performed and the morphology, size, and typical biomarkers (CD9,CD63, CD81, and TSG101)were analyzed as outlined previously [22].

### Plasmid construction and cell transfection

To knock down circPDLIM5, two siRNAs that specifically targeted the junction site of circPDLIM5 were designed and synthesized by GenePharma (Suzhou, China), and siRNA-NC was used as a control. Then, the two siRNAs and siRNA-NC were subcloned into the lentiviral vector to construct the sh-circPDLIM5#1 vectorand sh-circPDLIM5#2 vector, and the sh-NC vector was used as a control. For the overexpression of circPDLIM5, the lentivirus carryingthe EF-1aF-circPDLIM5 vector was designed and synthesized by GenePharma (Suzhou, China), and the control vector without a circPDLIM5 sequence was used as a control. To select stably transfected cells, the lentiviruses carryingthe EF-1aF-circPDLIM5 vector, control vector, sh-circPDLIM5#1 vector, sh-circPDLIM5#2 vector, and sh-NC vector were added to PC3 and 22RV1 cells in combination with 1 μl Polybrene (5 μg/μl) (GenePharma, Suzhou, China). After 48 h, puromycinwas added to the medium at a concentration of 5 μg per 1 ml of medium. After 72 h, the cells were no longer dead, and stable transfected cells were constructed for further research. To knockdown hnRNPA2B1, YY1, and PROX1, siRNAs were designed and synthesized by RiboBio (Guangzhou, China).PCa cells and HLECs were cultivated in 6-well plates, and siRNAs were transfected into PCa cells and HLECs with Lipofectamine 3000 (Invitrogen, Carlsbad, CA, USA) according to the manufacturer’s instructions. After 72 h, qRT-PCR and western blotting were used to confirm the knockdown efficiency of hnRNPA2B1 in PCa cells and the knockdown efficiency of YY1 and PROX1 in HLECs, separately. The sequences ofthe shRNAs and siRNAs are described in Supplemental Table 5.

### Fluorescent labeling and EV internalization

A PKH67 Green Fluorescent Cell Linker Kit (Umibio, Shanghai, China) was used to label the EVs derived from PCa cells. Briefly, 1 μl of PKH67 Linker (Umibio) was added into 9 μl of Diluent C (Umibio) and mixed well. Then, 1 μl of PKH67 solution was added into 10 μg of EVs and incubated at room temperature for 10 min in the dark. Following this, 30 ml PBS was added into the EVs and mixed well, after which the mixture was centrifuged at 170,000 × g for 70 min at 4 °C. The pellets containing the PKH67-labeled EVs were then resuspended for furtheruse. Subsequently, 10 μg/mL of PKH67-labeled EVs were incubated with HLECs for 12 h. After the nuclei of HLECs were stained with 4′,6-diamidino-2-phenylindole (DAPI), the internalization of the EVs was photographed through confocal fluorescence microscopy (Carl Zeiss AG, Jenna, Germany).

### Tube formation assays and Transwell migration assays

For the tube formation assays, 200 μl of 1:1 medium containing Matrigel (BDBiosciences, Bedford, Massachusetts, USA) and ECM medium was added to a 24-well plate and allowed to solidify for 1 h at 37 °C. Then, HLECs were trypsinized and approximately 5 × 10^4^ of these HLECs were seeded in the 24-well plate and incubated with 20 μg of EVsor with PBS for 12 h at 37 °C with 5% CO_2_. Imageswere taken in a bright field by a fluorescence microscope(Olympus, Tokyo, Japan), and the length of the tube structure was measured. HLEC Transwell migration assays were performed according to our previous report [56].

### RNA/gDNA extraction

Total RNA was extracted from PCa cell lines, HLECs, and PCa tissuesusing TRIzol reagent (Invitrogen, Carlsbad, CA, USA) according to the manufacturer’s protocol. Briefly, approximately 2 × 10^6^ cells or 100 mg of tissuewere incubated with 1 ml of TRIzol reagent for 10 min at room temperature, after which 200 μl of chloroform was added to the lysis buffer for 5 min at 4 °C, followed by centrifugation for 15 min at 12,000 × g and 4 °C. After centrifugation, the aqueous phase containing the RNA was transferred to a new tube, and an equal volume of isopropanol was added to the aqueous phase for 10 min at 4 °C, followed by centrifugation at 12,000 × g for 10 min at 4 °C. Next, the supernatant was discarded, and the retained pellet was combined with 1 ml of 75% ethanol before centrifuging it at 10,000 × g for 10 min at 4 °C. The supernatant was then mixed using a micropipettor to resuspend the RNA in 30–50 μl of RNase-free water. Genomic DNA (gDNA) was extracted fromPC3 and 22RV1 cells according to the PureLink Genomic DNAMini Kit protocol (Thermo Fisher Scientific, Waltham, MA, USA).

### qRT-PCR

In brief, 500 ng of total RNA was reverse transcribed to cDNA through the PrimeScript RT Reagent Kit (Takara, Tokyo, Japan) following the manufacturer’s instructions, after which TB Green Premix Ex Taq (Takara,Tokyo, Japan)was used for qRT-PCR analysis.The reactions were conducted on a RocheLightCycler® 480II PCR instrument (Basel, Switzerland), with GAPDH as an internal standard control. The relative RNA expression levels were calculated using the 2^–ΔΔCT^ method. The primers used in the study are described in Supplemental Table 6.

### Nuclear and cytoplasmic fractionation experiment

The nuclear and cytoplasmic fractionswere separated using a Cytoplasmic & Nuclear RNA Purification Kit (Thorold, ON, Canada). Briefly, approximately 1–3 × 10^6^ PC3 or 22RV1 cells were collected and washed twice with PBS. Next, 200 μlof ice-cold Lysis Buffer J was directly added to the cells, followed by gentle tappingwhile keeping it on ice for 5 min. Then, the lysis buffer sample was centrifuged at 14,000 g for 3 min at room temperature. The cytoplasmic fraction in the supernatant was carefully transferred to another tube, and the nuclear fraction was retained in the bottom pellet of the tube. Then, 200 μl or 400 μlofBuffer SK was added to the cytoplasmic fraction and nuclear fraction, respectively, after which 200 μl of 100% ethanol was added to the cytoplasmic and nuclear fractions. Following this, the mixtures were vortexed for 10–15 s and centrifuged at 4000 × g for 1 min at room temperature. Subsequently, 400 μlof Wash Solution A was added and centrifuged at 14,000 × g for 1 min at room temperature. Then, 40–50 μlof Elution Buffer E was added and centrifuged at 200 × g for 2 min, then at 14,000 × g for 1 min. Finally, the RNA was reverse transcribed to cDNAusing the PrimeScript RT Reagent Kit (Takara,Tokyo, Japan) following the manufacturer’s instructions and stored at − 80 °C for further use.

### RNase R treatment

The RNA extracted from the PC3 and 22RV1 cells was treated with RNase R (1.5 U/μg) (Geenseed, Guangzhou, China) for 30 min at 37 °C, while an equal amount of total RNA without RNase R was incubated in the same environment as the control.Then, the stability of circPDLIM5 and its liner counterpart was measured using qRT–PCR.

### Electrophoresisanalysis

To obtain the 1.5% agarose gel, 0.75 g of agarose was added to 50 ml 1 × TAE buffer and heated to a boil, after which it was cooled to 70 °C–80 °C.A total of 5 μl of 4S GelRed (Sangon Biotech, Shanghai, China) was then added to it, and the mixture was mixed thoroughly. Next, the liquid solution was poured into a mold until the gel was completely cooled. The mold containing the gel was placed into an electrophoresis tank with 1 × TAE buffer. Approximately 10 μl of DNA samples mixed with loading buffer were added to each well, and electrophoresis was conducted at 120 V for 30 min. All of the bands on the gel were photographed usingan ultraviolet imaging system.

### IHC and ISH

For IHC, theparaffin sections were deparaffinized and dehydrated by xylene and a series of graded ethanol. Then, the sections were treated with proteinase K at 37 °C for 20 min, followed by fixation with 3.7% paraformaldehyde for 10 min. Subsequently, the sections were subjected to antigen retrieval by trisodium citrate dehydrate buffer under high-pressure conditions, after which the sections were blocked with 1% bovine albumin (BSA) for 30 min at 37 °C. Next, the primary antibodies against VEGF-C (YT5297, 1:250, Immunoway, Plano, Texas, USA), LYVE-1 (ab219556, 1:10,000, Abcam, Burlingame, CA, USA),Renilla Luciferase (GTX125851, 1:350, GeneTex, Irvine, CA, USA), and PROX1 (ab199359, 1:500, Abcam, Burlingame, CA, USA) were incubated with the paraffin sections at 4 °C overnight. Following incubation, the paraffin sections were incubated with a secondary antibody (bs-0295G, 1:1000, Bioss, Beijing, China) for 1 h at room temperature. After three washes with PBS, the sections were stained with diaminobenzidine and hematoxylin, and they were then dehydrated by a series of graded ethanol and xylene, respectively. Finally, neutral balata was used to fix the sections, which were then photographed using a fluorescence microscope (Olympus, Tokyo, Japan).TheIHC score was measured as follows: 1) staining area score: 0, < 5%; 1, 5%–25%; 2, 25%–50%;3, 50%–75%; and 4, > 75%; 2) staining intensity score: 0, no staining; 1, weak staining; 2, moderate staining; and 3, intense staining; 3). Finally, the total staining score was estimated by combining the staining intensity and area scores; samples with a score ≥ 6 wereconsidered to indicate high expression, while those with a score < 6 wereconsidered to have a low expression [57].

For ISH, a 5′-digoxin (DIG)-labeled probe that specifically targets circPDLIM5 was designed and synthesized by Servicebio (Wuhan, China). The U6 probeand scramble probe were used as the internal control and negative control, respectively. In brief, the paraffin sections were deparaffinized, dehydrated, digested, and fixed with paraformaldehyde as mentioned previously, in the section on IHC. Then, the sections were incubated with hybridization buffer containing the circPDLIM5 probe at 37 °C overnight, followed by incubation with the anti-digoxin antibody at 4 °C overnight. Then, 5-Bromo-4-Chloro-3-Indolylphosphate/NitroblueTetrazolium (BCIP/NBT) was used to stain the sections for 30 min, after which they were photographed with an Olympus microscope(Olympus, Tokyo, Japan).The H-score of circPDLIM5 was assessed as follows: H-score = Σ (P × I), where P is the percentage of stained cells and I is the staining intensity score, which was categorized as follows: 0 (no staining), 1 (weak staining), 2 (moderate staining), and 3 (intense staining) (8). The probes used in the ISH are listed in Supplemental Table 7.

### Western blotting

Proteins were extracted from PCa/HLECs cells and exosomes using a Total Protein Extraction Kit (KeyGEN, Nanjing, China), and their concentrations were measured using a BCAprotein assay kit (KeyGEN, Nanjing, China) in accordance witha previously outlined procedure(52). Antibodies against CD9 (ab92726, 1:2000), CD81 (ab109201, 1:2500), TSG101 (ab125011, 1:5000), hnRNPA2B1 (ab31645, 1:400), YY1 (ab109228, 1:2000), PROX1 (ab199359, 1:1000), GAPDH (ab245355, 1:5000), and β-tubulin (ab179513, 1:1000)were obtained from Abcam (Burlingame, CA, USA).The secondary antibody (bs-0295G, 1:5000) was provided by Bioss (Beijing, China). Chemiluminescent signals were detected using Western ECL Substrate (Advansta, Menlo Park, CA, USA) and images were taken with a ChemiDoc Imaging System (Bio-Rad,Hercules, CA, USA).

### ELISA

A Human VEGF-C ELISA kit (ab100664, Abcam, Burlingame, CA, USA) was used to estimate the expression level of VEGF-C protein secreted in the medium of PCa cells with circPDLIM5 knockdown or overexpression according to the manufacturer’s instructions.

### Serial deletion circPDLIM5 sequence and mutation of the binding sites

For the serial deletion of the circPDLIM5 sequence and the mutation of the binding sites (851–927 nt region) of circPDLIM5 mentioned in the study, relevant sequences were designed and synthesizedthroughchemical gene synthesis. The plasmids were then transfected using Lipofectamine 3000 (Invitrogen, Carlsbad, CA, USA) according to the manufacturer’s instructions.

### RNA FISH

To estimate the distribution of circPDLIM5 in PCa cells, as well as the expression level and location of circPDLIM5 in PCatissues and benign prostatic hyperplasiatissues, a Cy3-labeled probe that could specifically target circPDLIM5, was designed and synthesized by RiboBio (Guangzhou, China). Briefly, for PCa cells, 2000 cells were placed into a 48-well plate with cover glass, and when the cells reached 70%–90% confluence, the cells were washed three times with PBS. After washing, 3.7% paraformaldehyde and 0.5% Triton-100 were used to fix and permeate the cells, respectively. Subsequently, the cells were pre-hybridized for 30 min at 37 °C with a pre-hybridization buffer, after which the hybridization buffer mixed with the FISH probe was incubated with the cells overnight at 37 °C in the dark. Following incubation, the cells were washed with SSC solution and the nuclei were stained with DAPI for 15 min. For PCa and benign prostatic hyperplasia tissues, the paraffin sections were deparaffinized and dehydrated by xylene and a series of graded ethanol, and they were then treatedwith proteinaseK for 5–10 min. The remaining processing steps were the same as those for PCa cells. The results were photographed by confocal fluorescence microscope (Carl Zeiss AG, Jenna, Germany). The probes used in the FISH assay are listed in Supplemental Table 7.

### Colocalization of circPDLIM5 with hnRNPA2B1/YY1

The colocalization of circPDLIM5 with hnRNPA2B1 in PCa cells and circPDLIM5 with YY1 in HLECs was measured by fluorescence staining. Briefly, for PCa cells, 2000 cells were placed into a 48-well plate with cover glass and cultured for 48 h. Then, the cells were washed three times with PBS, followed by incubation with 3.7% paraformaldehyde for 15 min and 0.5% Triton-100 for 5 min. Following incubation, the cells were pre-hybridized for 30 min at 37 °C with pre-hybridization buffer, followed by hybridization buffer containing Cy3-labeled circPDLIM5 probe(RiboBio, Guangzhou, China) overnight at 37 °C in dark. The cells were then permeated with 0.5% Triton-100 for 5 min, followed by incubation with an anti-hnRNPA2B1 antibody (Abcam, Burlingame, CA, USA) overnight at 4 °C in the dark with mild rotation. Following incubation, the cells were washed with PBS and the nuclei werestained with DAPI for 15 min. To determinethe colocalization of circPDLIM5 with YY1 in HLECs, HLECs incubated with EVs-circPDLIM5 from PCa cells were trypsinized and counted. Next, 2000 cells were plated into a 48-well plate with cover glass and cultured for 48 h. The remaining steps were the same as those outlined for PCa cells. Finally, the cells were photographed using aconfocal fluorescence microscope (Carl Zeiss AG, Jenna, Germany).

### RNA pull-down assay using a specific biotin-coupled probe

The biotin-coupled circPDLIM5 probe and oligo probe (RiboBio, Guangzhou, China) were used to pull down the proteins that interacted with circPDLIM5. Briefly, approximately 1 × 10^7^ PCa cells with circPDLIM5 overexpression or 1 × 10^7^ HLECs incubated with EVs derived from PCa cells transfected with circPDLIM5 were lysed and sonicated in a 4 °C water bath for 2–3 h. Then, the lysate was mixed with the probes (100 pg) at room temperature for 16–24 h, after which the streptavidin magnetic beads (MCE, Monmouth Junction, NJ, USA) were co-incubated with the lysis solution at room temperature for 2–4 h. Next, the lysis solution was set on a magnetic stand for 1 min and washed with washing buffer five times, and then 1 ml of the washing buffer was used to resuspend the magnetic beads, of which 100 μl was used to extract RNA and 900 μl was used to extract protein. For protein extraction, 20 μl of 5 × loading buffer was placed into the 900 μl sample and incubated at 100 °C for 10 min before the magnetic beads and supernatant were separated on a magnetic stand, with the supernatant containing the protein product. The protein was further used for MS analysis and western blot. For RNA extraction, the 100-μl sample was mixed with proteinase K (Sangon Biotech, Shanghai, China) and RNA PK buffer at 50 °C for 45 min, followed by 95 °C for 10 min to break the formaldehyde cross-links. The RNA was purified by TRIzol reagent (Invitrogen, Carlsbad, CA, USA) and the abundance of circPDLIM5 was estimated by qRT-PCR. The sequences ofthe circPDLIM5probe and oligo probe were as follows:

- circPDLIM5 Probe:5′-CTTTTAGCTCCTTGGTTTGG −3′;
- Oligo Probe:5′-AGTCGGAACTGAAATCATGT −3′.

### RNA immunoprecipitation (RIP) assay

To confirm the direct interaction between circPDLIM5 with hnRNPA2B1 in PCa cells, and circPDLIM5 with YY1 in HLECs, RIP assays were performed using a RIP kit (Millipore, MA,USA). Briefly,almost 2 × 10^7^ PCa cells transfected with circPDLIM5 or 2 × 10^7^ HLECs incubated with EVs-circPDLIM5 derived from PCa cells and transfected with circPDLIM5 were collected and lysed. First, 50 μl of the lysate was transferred into a new tube as input. Then, 5 μg of anti-hnRNPA2B1 antibody(Abcam, ab31645, Burlingame, CA, USA) or anti-YY1 antibody (Abcam, ab109228, Burlingame, CA, USA) and 5 μg of anti-IgG antibody (Cell Signaling Technology, Beverly, MA, USA) were incubated with the cell lysate for 16 h at 4 °C with mild rotation. Subsequently, approximately 5 μg of A/G protein magnetic beads were added to the cell lysate and incubated overnight at 4 °C with mild rotation. Then, the coprecipitated RNAs were purifiedusing aTRIzol reagent (Invitrogen, Carlsbad, CA, USA), and the abundance of RNAs was estimated through qRT-PCR.

### Mass spectrometry

The proteins that were determined to interact with circPDLIM5 through an RNA pull-down assay were used for MS analysis. In brief, the protein samples were analyzed using an Ultimate 3000 RSLCnano system coupled with a Q Exactive HF mass spectrometer through a Nanospray Flex ion source (Thermo Fisher Scientific, Waltham, MA, USA). The peptide fragments were analyzedusing theNational Biotechnology Information Center’s non-redundant protein database and Mascot (Matrix Science, USA).

### ChIP assay

AnEZ-ChIP assay kit (Millipore, MA, USA) was used to demonstrate the interaction between YY1/hnRNPA2B1 protein with the DNA of PROX1. In brief, 2 × 10^7^HLECs incubated with EVs-circPDLIM5 were collected and lysed. Then, 50 μl of lysate was transferred to a new tube as the input. Subsequently, the lysis solution was sonicated for approximately 30 min with protease inhibitors until the lysate was no longer turbid. Following this, 5 μg of anti-YY1 antibody (Abcam, ab109228,Burlingame, CA, USA), anti-hnRNPA2B1 antibody(Abcam, ab31645, Burlingame, CA, USA), or anti-IgG antibody (Cell Signaling Technology, Beverly, MA, USA) was incubated with the cell lysate for 16 h at 4 °C with mild rotation. Next, A/G protein coupled with magnetic beads were added to the cell lysate and incubated overnight at 4 °C with mild rotation, after which the retrieved DNA was measured via qRT-PCR.

### ChIRP assay

The circPDLIM5 probe thattargeted the back-spliced site of circPDLIM5 was designed and synthesized by RiboBio (Guangzhou, China), and the oligo probe was used as a negative control. Briefly, 2 × 10^7^ HLECs incubated withEVs-circPDLIM5 derived from 22RV1 were lysed and sonicated in a 4 °C water bath for 2–3 h. Approximately 50 µl of the lysate was transferred to a new tube as the input. Then, the lysate was mixed with the probes(100 pg) at room temperature for 16–24 h, before co-incubating the streptavidin magnetic beads (MCE, Monmouth Junction, NJ, USA) with the lysis solution at room temperature for 2–4 h. Subsequently, the lysis solution was set on a magnetic stand for 1 min and washed with washing buffer five times. Then, the DNA waspurified from post-ChIRP beads, and the enrichment of DNAretrieval wasestimated through qRT-PCR. The sequences ofthe circPDLIM5probe and oligo probe were as follows:

- CircPDLIM5 Probe:5′-CTTTTAGCTCCTTGGTTTGG −3′;
- Oligo Probe:5′-AGTCGGAACTGAAATCATGT −3′.

### Bioinformatics analysis

The catRAPID platform (http://service.tartaglialab.com/page/catrapid_group) was used to predict the region where circPDLIM5 interacted with hnRNPA2B1. The RNAInter platform (http://www.rna-society.org/rnainter/) and JASPAR (http://jaspar.genereg.net/) were applied to predict the relationship between circPDLIM5, YY1, and PROX1.

### Statistical analysis

All statistical analyses were performed using SPSS 20.0(SPSS, Chicago, IL, USA), R software (version 3.6.1), and GraphPad Prism 8.0 (GraphPad Software Inc, CA, USA). All of the in vitro experiments were performed in triplicate, and the results are presented as the mean ± SD. Student’s t-test, the Mann–Whitney U test,one-way analysis of variance, or the chi-square test was used to analyze the differences between groups. Kaplan–Meier curves and the log-rank test were used to evaluate the PFS. The Cox proportional hazards regression model was constructed to estimate the impact of circPDLIM5 on the PFS. The correlations between groups were assessed using Pearson correlation analysis. *P* values < 0.05 were considered statistically significant.

## Supplementary Information

Supplementary Material 1.

Supplementary Material 3. Supplemental Table 8 Mass spectrometry results revealed the proteins interacted with circPDLIM5 in PC3 cells.

Supplementary Material 4. Supplemental Table 9 Mass spectrometry results revealed the proteins interacted with circPDLIM5 in HLECs treated with EVs-circPDLIM5.

Supplementary Material 4.

## Data Availability

Sequence data that support the findings of this study have been deposited in the National Center for Biotechnology Information’s Gene Expression Omnibus (https://www.ncbi.nlm.nih.gov/geo/query/acc.cgi?acc=GSE147761).

## Abbreviations

ATCC: American Type Culture Collection
BCIP/NBT: 5-Bromo-4-Chloro-3-Indolylphosphate/Nitroblue Tetrazolium
circRNA: Circular RNA
circPDLIM5: Circular RNA PDLIM5
cDNA: Complementary DNA
ChIP: Chromatin immunoprecipitation
ChIRP: Chromatin isolation by RNA purification
DIG: Digoxin
DAPI: 4′,6-Diamidino-2-phenylindole
EVs: Extracellular vesicles
ELISA: Enzyme linked immunosorbent assay
FBS: Fetal bovine serum
FISH: Fluorescence in situ hybridization
FISH-IF: Fluorescence in situ hybridization and immunofluorescence
gDNA: Genomic DNA
hnRNPA2B1: Heterogeneous nuclear ribonucleoprotein A2B1
HLECs: Human lymphatic endothelial cells
HE: Hematoxylin–eosin
IHC: Immunohistochemistry
ISH: In situ hybridization
LN: Lymph node
LYVE-1: Lymphatic vessel endothelial hyaluronan receptor 1
MS: Mass spectrometry
PCa: Prostate cancer
PROX1: Prospero homeobox 1
PBS: Phosphate buffered saline
PFS: Progression-free survival
qRT-PCR: Quantitative real-time PCR
RWPE-1: Human prostate epithelial cell line
RIP: RNA immunoprecipitation
ROC: Receiver operating characteristic
shRNA: Short hairpin RNA
si-RNA: Small interfering RNA
TEM: Transmission electron microscopy
WB: Western blot
WT: Wild type
YY1: Transcription factor Yin Yang 1

## References

[1] Sartor O, de Bono JS. Metastatic prostate cancer. N Engl J Med. 2018;378:645–57. 29412780 10.1056/NEJMra1701695

[2] Wilczak W, Wittmer C, Clauditz T, Minner S, Steurer S, Büscheck F, Krech T, Lennartz M, Harms L, Leleu D, et al. Marked prognostic impact of minimal lymphatic tumor spread in prostate cancer. Eur Urol. 2018;74:376–86. 29908878 10.1016/j.eururo.2018.05.034

[3] Isaac R, Reis FCG, Ying W, Olefsky JM. Exosomes as mediators of intercellular crosstalk in metabolism. Cell Metab. 2021;33:1744–62. 34496230 10.1016/j.cmet.2021.08.006 PMC8428804

[4] Kalluri R, LeBleu VS. The biology, function, and biomedical applications of exosomes. Science. 2020;367:eaau6977. 32029601 10.1126/science.aau6977 PMC7717626

[5] Becker A, Thakur BK, Weiss JM, Kim HS, Peinado H, Lyden D. Extracellular vesicles in cancer: cell-to-cell mediators of metastasis. Cancer Cell. 2016;30:836–48. 27960084 10.1016/j.ccell.2016.10.009 PMC5157696

[6] Zhang H, Deng T, Liu R, Bai M, Zhou L, Wang X, Li S, Wang X, Yang H, Li J, et al. Exosome-delivered EGFR regulates liver microenvironment to promote gastric cancer liver metastasis. Nat Commun. 2017;8:15016. 28393839 10.1038/ncomms15016 PMC5394240

[7] Zhao S, Mi Y, Guan B, Zheng B, Wei P, Gu Y, Zhang Z, Cai S, Xu Y, Li X, et al. Tumor-derived exosomal miR-934 induces macrophage M2 polarization to promote liver metastasis of colorectal cancer. J Hematol Oncol. 2020;13:156. 33213490 10.1186/s13045-020-00991-2 PMC7678301

[8] García-Silva S, Benito-Martín A, Nogués L, Hernández-Barranco A, Mazariegos MS, Santos V, Hergueta-Redondo M, Ximénez-Embún P, Kataru RP, Lopez AA, et al. Melanoma-derived small extracellular vesicles induce lymphangiogenesis and metastasis through an NGFR-dependent mechanism. Nat Cancer. 2021;2:1387–405. 34957415 10.1038/s43018-021-00272-y PMC8697753

[9] Zhou CF, Ma J, Huang L, Yi HY, Zhang YM, Wu XG, Yan RM, Liang L, Zhong M, Yu YH, et al. Cervical squamous cell carcinoma-secreted exosomal miR-221–3p promotes lymphangiogenesis and lymphatic metastasis by targeting VASH1. Oncogene. 2019;38:1256–68. 30254211 10.1038/s41388-018-0511-x PMC6363643

[10] Sun B, Zhou Y, Fang Y, Li Z, Gu X, Xiang J. Colorectal cancer exosomes induce lymphatic network remodeling in lymph nodes. Int J Cancer. 2019;145:1648–59. 30734278 10.1002/ijc.32196

[11] Li M, Lu Y, Xu Y, Wang J, Zhang C, Du Y, Wang L, Li L, Wang B, Shen J, et al. Horizontal transfer of exosomal CXCR4 promotes murine hepatocarcinoma cell migration, invasion and lymphangiogenesis. Gene. 2018;676:101–9. 30010038 10.1016/j.gene.2018.07.018

[12] Chen C, Luo Y, He W, Zhao Y, Kong Y, Liu H, Zhong G, Li Y, Li J, Huang J, et al. Exosomal long noncoding RNA LNMAT2 promotes lymphatic metastasis in bladder cancer. J Clin Invest. 2020;130:404–21. 31593555 10.1172/JCI130892 PMC6934220

[13] Gong X, Tian M, Cao N, Yang P, Xu Z, Zheng S, Liao Q, Chen C, Zeng C, Jose PA, et al. Circular RNA circEsyt2 regulates vascular smooth muscle cell remodeling via splicing regulation. J Clin Invest. 2021;131:e147031. 34907911 10.1172/JCI147031 PMC8670847

[14] Chen Z, Lu T, Huang L, Wang Z, Yan Z, Guan Y, Hu W, Fan Z, Zhu P. Circular RNA cia-MAF drives self-renewal and metastasis of liver tumor-initiating cells via transcription factor MAFF. J Clin Invest. 2021;131:e148020. 34403373 10.1172/JCI148020 PMC8483755

[15] He T, Tao W, Zhang LL, Wang BY, Li K, Lu HM, Tang GJ, He YD, Li LY. CircSCAF8 promotes growth and metastasis of prostate cancer through the circSCAF8-miR-140-3p/miR-335-LIF pathway. Cell Death Dis. 2022;13:517. 35654787 10.1038/s41419-022-04913-7 PMC9163066

[16] Xu H, Sun Y, You B, Huang CP, Ye D, Chang C. Androgen receptor reverses the oncometabolite R-2-hydroxyglutarate-induced prostate cancer cell invasion via suppressing the circRNA-51217/miRNA-646/TGFβ1/p-Smad2/3 signaling. Cancer Lett. 2020;472:151–64. 31846689 10.1016/j.canlet.2019.12.014

[17] Yang Z, Qu CB, Zhang Y, Zhang WF, Wang DD, Gao CC, Ma L, Chen JS, Liu KL, Zheng B, et al. Dysregulation of p53-RBM25-mediated circAMOTL1L biogenesis contributes to prostate cancer progression through the circAMOTL1L-miR-193a-5p-Pcdha pathway. Oncogene. 2019;38:2516–32. 30531834 10.1038/s41388-018-0602-8 PMC6484770

[18] Cao S, Ma T, Ungerleider N, Roberts C, Kobelski M, Jin L, Concha M, Wang X, Baddoo M, Nguyen HM, et al. Circular RNAs add diversity to androgen receptor isoform repertoire in castration-resistant prostate cancer. Oncogene. 2019;38:7060–72. 31409897 10.1038/s41388-019-0947-7 PMC6842090

[19] Tao W, Wang BY, Luo L, Li Q, Meng ZA, Xia TL, Deng WM, Yang M, Zhou J, Zhang X, et al. A urine extracellular vesicle lncRNA classifier for high-grade prostate cancer and increased risk of progression: a multi-center study. Cell Rep Med. 2023;4:101240. 37852185 10.1016/j.xcrm.2023.101240 PMC10591064

[20] Luo Y, Ma J, Liu F, Guo J, Gui R. Diagnostic value of exosomal circMYC in radioresistant nasopharyngeal carcinoma. Head Neck. 2020;42:3702–11. 32945062 10.1002/hed.26441

[21] Zheng Y, Li JX, Chen CJ, Lin ZY, Liu JX, Lin FJ. Extracellular vesicle-derived circ_SLC19A1 promotes prostate cancer cell growth and invasion through the miR-497/septin 2 pathway. Cell Biol Int. 2020;44:1037–45. 31903637 10.1002/cbin.11303

[22] He YD, Tao W, He T, Wang BY, Tang XM, Zhang LM, Wu ZQ, Deng WM, Zhang LX, Shao CK, et al. A urine extracellular vesicle circRNA classifier for detection of high-grade prostate cancer in patients with prostate-specific antigen 2–10 ng/mL at initial biopsy. Mol Cancer. 2021;20:96. 34301266 10.1186/s12943-021-01388-6 PMC8299620

[23] Ducoli L, Detmar M. Beyond PROX1: transcriptional, epigenetic, and noncoding RNA regulation of lymphatic identity and function. Dev Cell. 2021;56:406–26. 33621491 10.1016/j.devcel.2021.01.018

[24] Liu L, Lin C, Liang W, Wu S, Liu A, Wu J, Zhang X, Ren P, Li M, Song L. TBL1XR1 promotes lymphangiogenesis and lymphatic metastasis in esophageal squamous cell carcinoma. Gut. 2015;64:26–36. 24667177 10.1136/gutjnl-2013-306388

[25] Chen C, He W, Huang J, Wang B, Li H, Cai Q, Su F, Bi J, Liu H, Zhang B, et al. LNMAT1 promotes lymphatic metastasis of bladder cancer via CCL2 dependent macrophage recruitment. Nat Commun. 2018;9:3826. 30237493 10.1038/s41467-018-06152-x PMC6148066

[26] Narla G, DiFeo A, Fernandez Y, Dhanasekaran S, Huang F, Sangodkar J, Hod E, Leake D, Friedman SL, Hall SJ, et al. KLF6-SV1 overexpression accelerates human and mouse prostate cancer progression and metastasis. J Clin Invest. 2008;118:2711–21. 18596922 10.1172/JCI34780 PMC2441856

[27] Villarroya-Beltri C, Gutiérrez-Vázquez C, Sánchez-Cabo F, Pérez-Hernández D, Vázquez J, Martin-Cofreces N, Martinez-Herrera DJ, Pascual-Montano A, Mittelbrunn M, Sánchez-Madrid F. Sumoylated hnRNPA2B1 controls the sorting of miRNAs into exosomes through binding to specific motifs. Nat Commun. 2013;4:2980. 24356509 10.1038/ncomms3980 PMC3905700

[28] Qin X, Guo H, Wang X, Zhu X, Yan M, Wang X, Xu Q, Shi J, Lu E, Chen W, Zhang J. Exosomal miR-196a derived from cancer-associated fibroblasts confers cisplatin resistance in head and neck cancer through targeting CDKN1B and ING5. Genome Biol. 2019;20:12. 30642385 10.1186/s13059-018-1604-0 PMC6332863

[29] Kumar A, Deep G. Hypoxia in tumor microenvironment regulates exosome biogenesis: Molecular mechanisms and translational opportunities. Cancer Lett. 2020;479:23–30. 32201202 10.1016/j.canlet.2020.03.017

[30] Mandriota SJ, Jussila L, Jeltsch M, Compagni A, Baetens D, Prevo R, Banerji S, Huarte J, Montesano R, Jackson DG, et al. Vascular endothelial growth factor-C-mediated lymphangiogenesis promotes tumour metastasis. Embo J. 2001;20:672–82. 11179212 10.1093/emboj/20.4.672 PMC145430

[31] Karpanen T, Egeblad M, Karkkainen MJ, Kubo H, Ylä-Herttuala S, Jäättelä M, Alitalo K. Vascular endothelial growth factor C promotes tumor lymphangiogenesis and intralymphatic tumor growth. Cancer Res. 2001;61:1786–90. 11280723

[32] Ji H, Hu C, Yang X, Liu Y, Ji G, Ge S, Wang X, Wang M. Lymph node metastasis in cancer progression: molecular mechanisms, clinical significance and therapeutic interventions. Signal Transduct Target Ther. 2023;8:367. 37752146 10.1038/s41392-023-01576-4 PMC10522642

[33] Astin JW, Haggerty MJ, Okuda KS, Le Guen L, Misa JP, Tromp A, Hogan BM, Crosier KE, Crosier PS. Vegfd can compensate for loss of Vegfc in zebrafish facial lymphatic sprouting. Development. 2014;141:2680–90. 24903752 10.1242/dev.106591

[34] Karnezis T, Shayan R, Caesar C, Roufail S, Harris NC, Ardipradja K, Zhang YF, Williams SP, Farnsworth RH, Chai MG, et al. VEGF-D promotes tumor metastasis by regulating prostaglandins produced by the collecting lymphatic endothelium. Cancer Cell. 2012;21:181–95. 22340592 10.1016/j.ccr.2011.12.026

[35] Cho H, Kim J, Ahn JH, Hong YK, Mäkinen T, Lim DS, Koh GY. YAP and TAZ negatively regulate Prox1 during developmental and pathologic lymphangiogenesis. Circ Res. 2019;124:225–42. 30582452 10.1161/CIRCRESAHA.118.313707

[36] Xu Y, Leng K, Yao Y, Kang P, Liao G, Han Y, Shi G, Ji D, Huang P, Zheng W, et al. A Circular RNA, Cholangiocarcinoma-Associated Circular RNA 1, Contributes to Cholangiocarcinoma Progression, Induces Angiogenesis, and Disrupts Vascular Endothelial Barriers. Hepatology. 2021;73:1419–35. 32750152 10.1002/hep.31493

[37] Xu C, Tsai YH, Galbo PM, Gong W, Storey AJ, Xu Y, Byrum SD, Xu L, Whang YE, Parker JS, et al. Cistrome analysis of YY1 uncovers a regulatory axis of YY1:BRD2/4-PFKP during tumorigenesis of advanced prostate cancer. Nucleic Acids Res. 2021;49:4971–88. 33849067 10.1093/nar/gkab252 PMC8136773

[38] Shinoda K, Zong D, Callen E, Wu W, Dumitrache LC, Belinky F, Chari R, Wong N, Ishikawa M, Stanlie A, et al. The dystonia gene THAP1 controls DNA double-strand break repair choice. Mol Cell. 2021;81:2611-2624.e2610. 33857404 10.1016/j.molcel.2021.03.034 PMC8985095

[39] Luo Y, Li Z, Kong Y, He W, Zheng H, An M, Lin Y, Zhang D, Yang J, Zhao Y, et al. KRAS mutant-driven SUMOylation controls extracellular vesicle transmission to trigger lymphangiogenesis in pancreatic cancer. J Clin Invest. 2022;132:e157644. 35579947 10.1172/JCI157644 PMC9282935

[40] Wigle JT, Harvey N, Detmar M, Lagutina I, Grosveld G, Gunn MD, Jackson DG, Oliver G. An essential role for Prox1 in the induction of the lymphatic endothelial cell phenotype. Embo J. 2002;21:1505–13. 11927535 10.1093/emboj/21.7.1505 PMC125938

[41] Lee S, Kang J, Yoo J, Ganesan SK, Cook SC, Aguilar B, Ramu S, Lee J, Hong YK. Prox1 physically and functionally interacts with COUP-TFII to specify lymphatic endothelial cell fate. Blood. 2009;113:1856–9. 18815287 10.1182/blood-2008-03-145789 PMC2647678

[42] Johnson NC, Dillard ME, Baluk P, McDonald DM, Harvey NL, Frase SL, Oliver G. Lymphatic endothelial cell identity is reversible and its maintenance requires Prox1 activity. Genes Dev. 2008;22:3282–91. 19056883 10.1101/gad.1727208 PMC2600759

[43] Petrova TV, Mäkinen T, Mäkelä TP, Saarela J, Virtanen I, Ferrell RE, Finegold DN, Kerjaschki D, Ylä-Herttuala S, Alitalo K. Lymphatic endothelial reprogramming of vascular endothelial cells by the Prox-1 homeobox transcription factor. Embo J. 2002;21:4593–9. 12198161 10.1093/emboj/cdf470 PMC125413

[44] Chen S, Huang V, Xu X, Livingstone J, Soares F, Jeon J, Zeng Y, Hua JT, Petricca J, Guo H, et al. Widespread and functional RNA circularization in localized prostate cancer. Cell. 2019;176:831-843.e822. 30735634 10.1016/j.cell.2019.01.025

[45] Yang F, Fang E, Mei H, Chen Y, Li H, Li D, Song H, Wang J, Hong M, Xiao W, et al. Cis-acting circ-CTNNB1 promotes β-catenin signaling and cancer progression via DDX3-mediated transactivation of YY1. Cancer Res. 2019;79:557–71. 30563889 10.1158/0008-5472.CAN-18-1559

[46] Dong X, Guo R, Ji T, Zhang J, Xu J, Li Y, Sheng Y, Wang Y, Fang K, Wen Y, et al. YY1 safeguard multidimensional epigenetic landscape associated with extended pluripotency. Nucleic Acids Res. 2022;50:12019–38. 35425987 10.1093/nar/gkac230 PMC9756953

[47] Liu D, Yang KY, Chan VW, Ye W, Chong CCN, Wang CC, Wang H, Zhou B, Cheng KKY, Lui KO. YY1 regulates glucose homeostasis through controlling insulin transcription in pancreatic β-cells. Diabetes. 2022;71:961–77. 35113157 10.2337/db21-0695 PMC9044128

[48] Liu T, Zhu Q, Kai Y, Bingham T, Wang S, Cha HJ, Mehta S, Schlaeger TM, Yuan GC, Orkin SH. Matrin3 mediates differentiation through stabilizing chromatin loop-domain interactions and YY1 mediated enhancer-promoter interactions. Nat Commun. 2024;15:1274. 38341433 10.1038/s41467-024-45386-w PMC10858947

[49] Alvarez-Erviti L, Seow Y, Yin H, Betts C, Lakhal S, Wood MJ. Delivery of siRNA to the mouse brain by systemic injection of targeted exosomes. Nat Biotechnol. 2011;29:341–5. 21423189 10.1038/nbt.1807

[50] Li C, Ni YQ, Xu H, Xiang QY, Zhao Y, Zhan JK, He JY, Li S, Liu YS. Roles and mechanisms of exosomal non-coding RNAs in human health and diseases. Signal Transduct Target Ther. 2021;6:383. 34753929 10.1038/s41392-021-00779-x PMC8578673

[51] Munagala R, Aqil F, Jeyabalan J, Kandimalla R, Wallen M, Tyagi N, Wilcher S, Yan J, Schultz DJ, Spencer W, Gupta RC. Exosome-mediated delivery of RNA and DNA for gene therapy. Cancer Lett. 2021;505:58–72. 33610731 10.1016/j.canlet.2021.02.011 PMC8005491

[52] Kim G, Kim M, Lee Y, Byun JW, Hwang DW, Lee M. Systemic delivery of microRNA-21 antisense oligonucleotides to the brain using T7-peptide decorated exosomes. J Control Release. 2020;317:273–81. 31730913 10.1016/j.jconrel.2019.11.009

[53] Hu C, Chen Q, Wu T, Du X, Dong Y, Peng Z, Xue W, Sunkara V, Cho YK, Dong L. The role of extracellular vesicles in the treatment of prostate cancer. Small. 2024;20:e2311071. 38639331 10.1002/smll.202311071

[54] Li LY, Liang SY, Cai MP, Ge JC, Tan HS, Wang CB, Xu B. Engineered extracellular vesicles as imaging biomarkers and therapeutic applications for urological diseases. Mater Today Bio. 2025;32:101646. 40160248 10.1016/j.mtbio.2025.101646 PMC11953971

[55] Crescitelli R, Lässer C, Lötvall J. Isolation and characterization of extracellular vesicle subpopulations from tissues. Nat Protoc. 2021;16:1548–80. 33495626 10.1038/s41596-020-00466-1

[56] Gao X, Pang J, Li LY, Liu WP, Di JM, Sun QP, Fang YQ, Liu XP, Pu XY, He D, et al. Expression profiling identifies new function of collapsin response mediator protein 4 as a metastasis-suppressor in prostate cancer. Oncogene. 2010;29:4555–66. 20543870 10.1038/onc.2010.213

[57] Piano MA, Brunello A, Cappellesso R, Del Bianco P, Mattiolo A, Fritegotto C, Montini B, Zamuner C, Del Fiore P, Rastrelli M, et al. Periostin and epithelial-mesenchymal transition score as novel prognostic markers for leiomyosarcoma, myxofibrosarcoma, and undifferentiated pleomorphic sarcoma. Clin Cancer Res. 2020;26:2921–31. 32127392 10.1158/1078-0432.CCR-19-2297

