# Supplementary material for: Extracellular vesicle-mediated transmission of circPDLIM5 promotes lymphatic metastasis in prostate cancer
Source: J Exp Clin Cancer Res. 2025 Jul 3;44:188. doi: 10.1186/s13046-025-03443-2 (PMC12224790; doi:10.1186/s13046-025-03443-2)
Supplement: Supplementary file 1 — Supplementary Material 1. [file 13046_2025_3443_MOESM1_ESM.pdf]

Extracellular vesicle-mediated transmission of circPDLIM5 promotes lymphatic metastasis in prostate cancer

Supplemental Material

Supplemental Figures

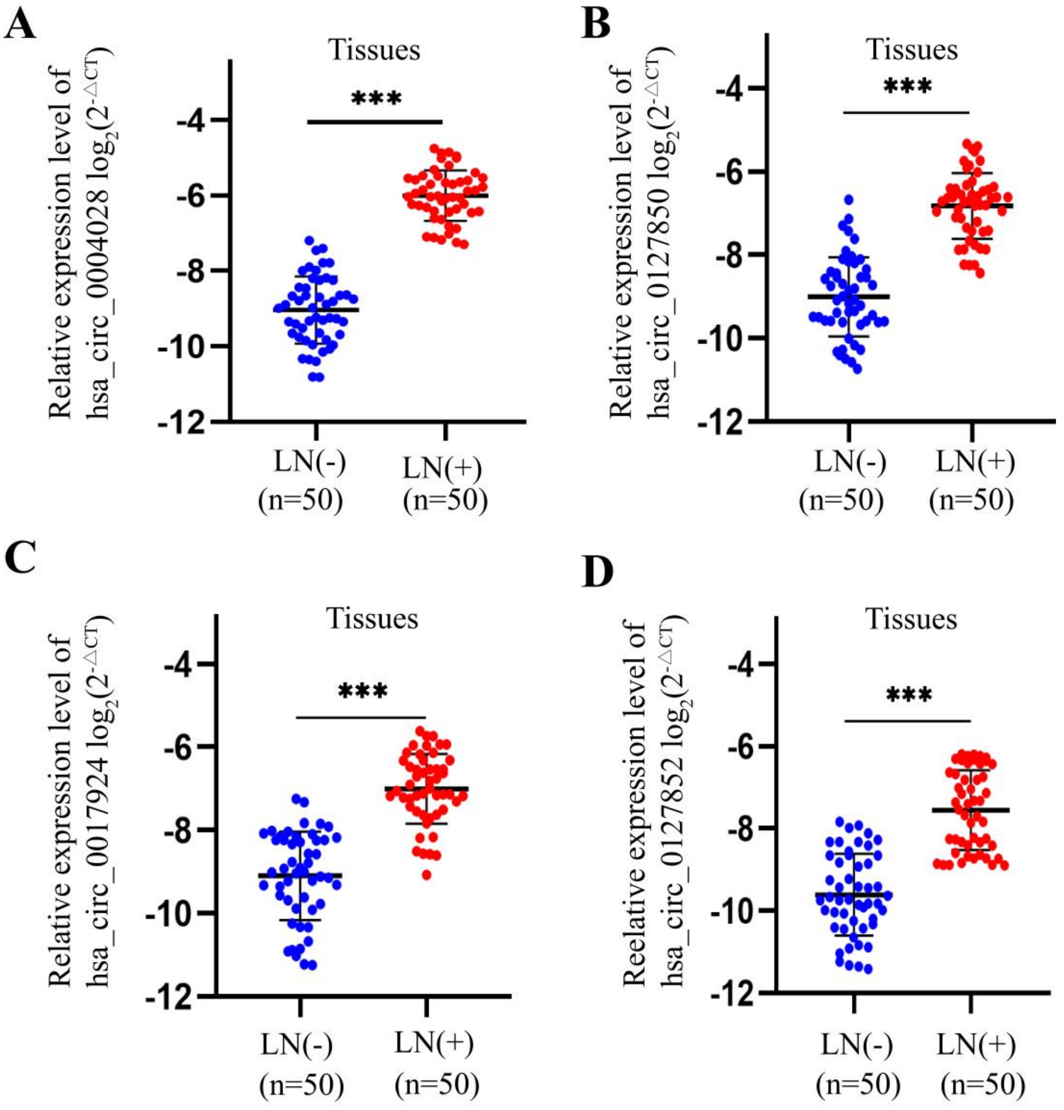

Supplemental Figure 1.The expression level of 4 candidate circRNAs in the LN(+) tumors and paired LN(-) tumors. **(A-D)** qRT-PCR analysis for the expression level of 4 candidate circRNAs in the LN (+) tumors and paired LN (-) tumors (n=50). **(A)** hsa_circ_0004028; **(B)** hsa_circ_0127850; **(C)** hsa_circ_0017924 and **(D)** hsa_circ_0127852. ***P<0.001. Statistical significance was assessed using nonparametric Mann-Whitney U test **(A-D)**.

A

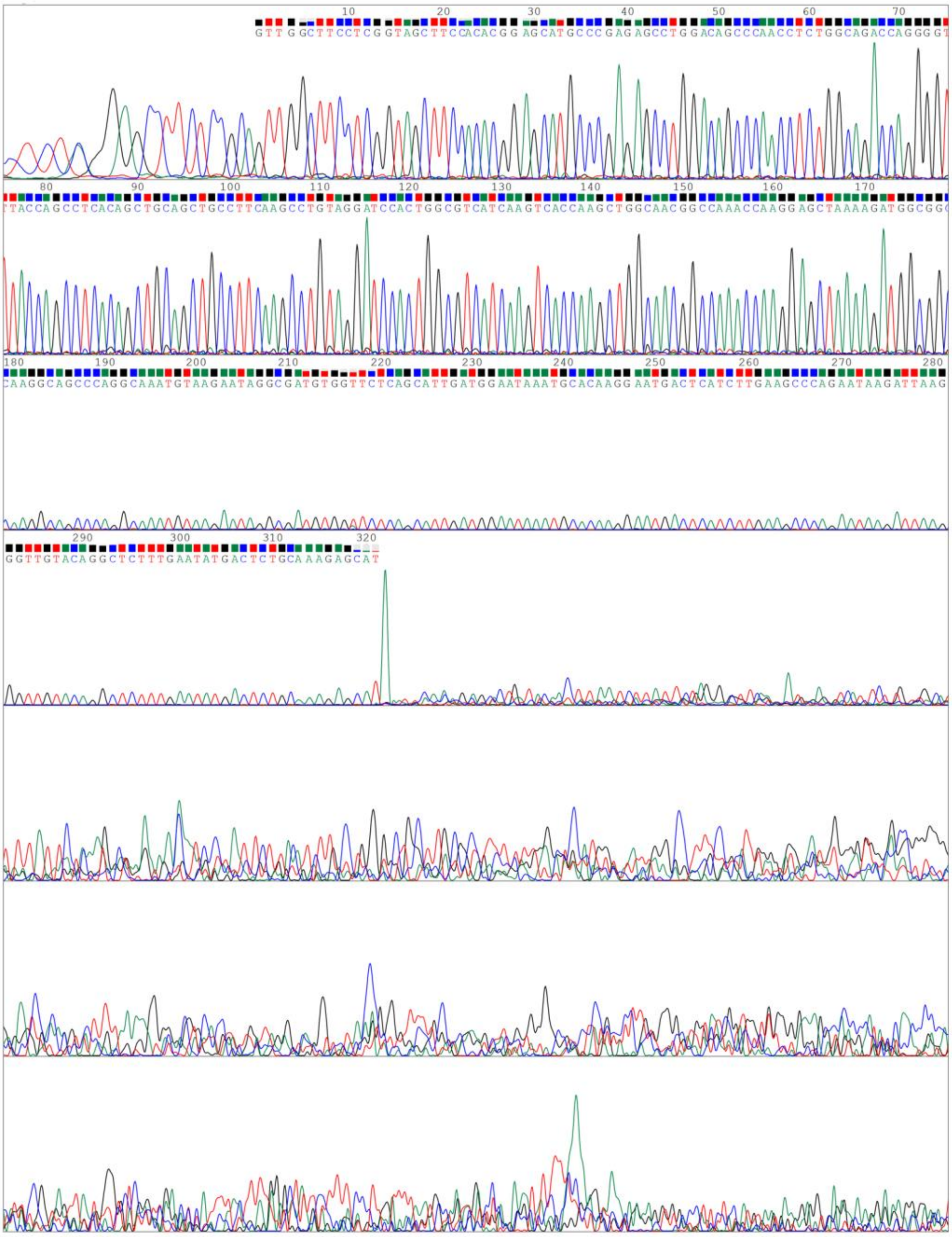

B

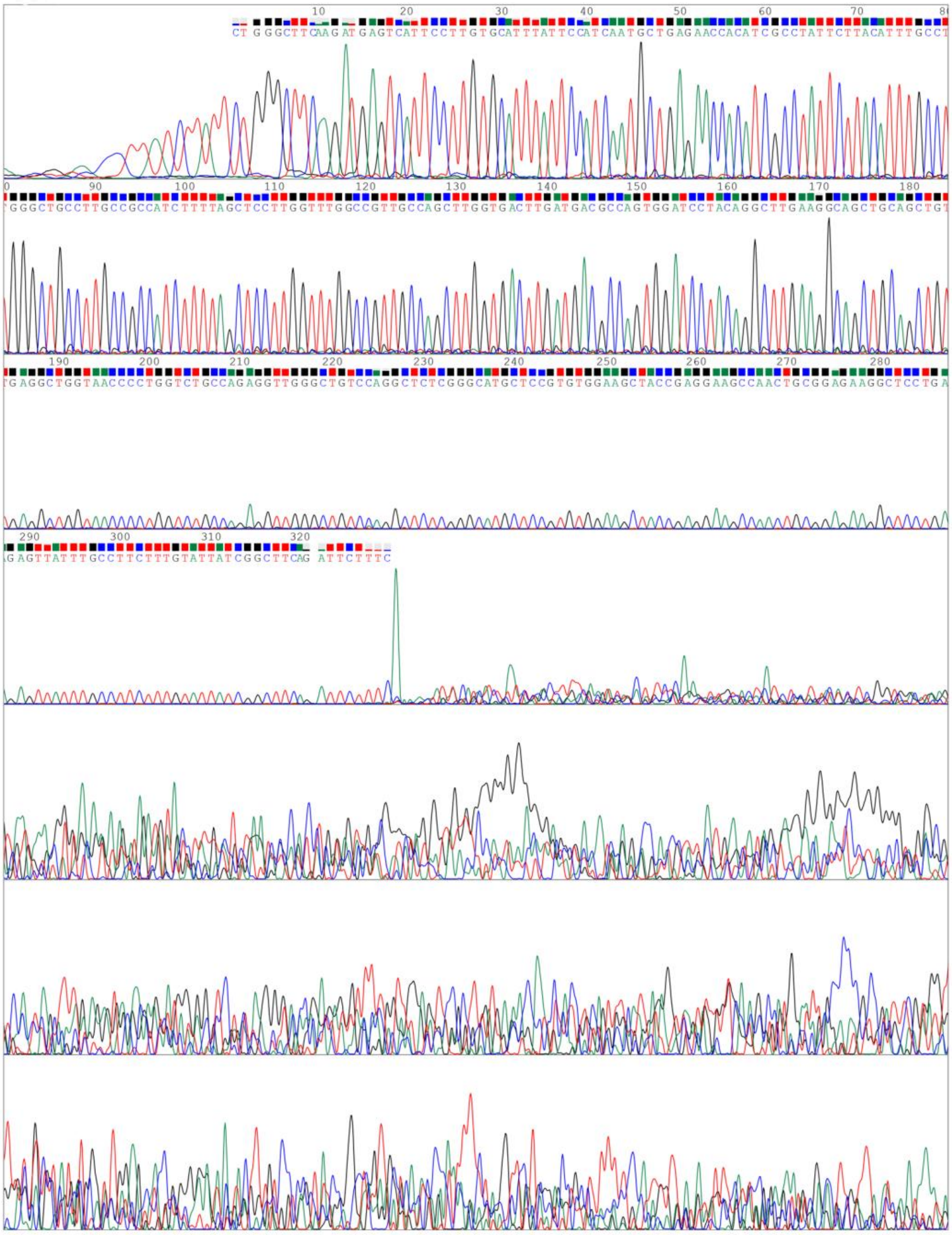

C

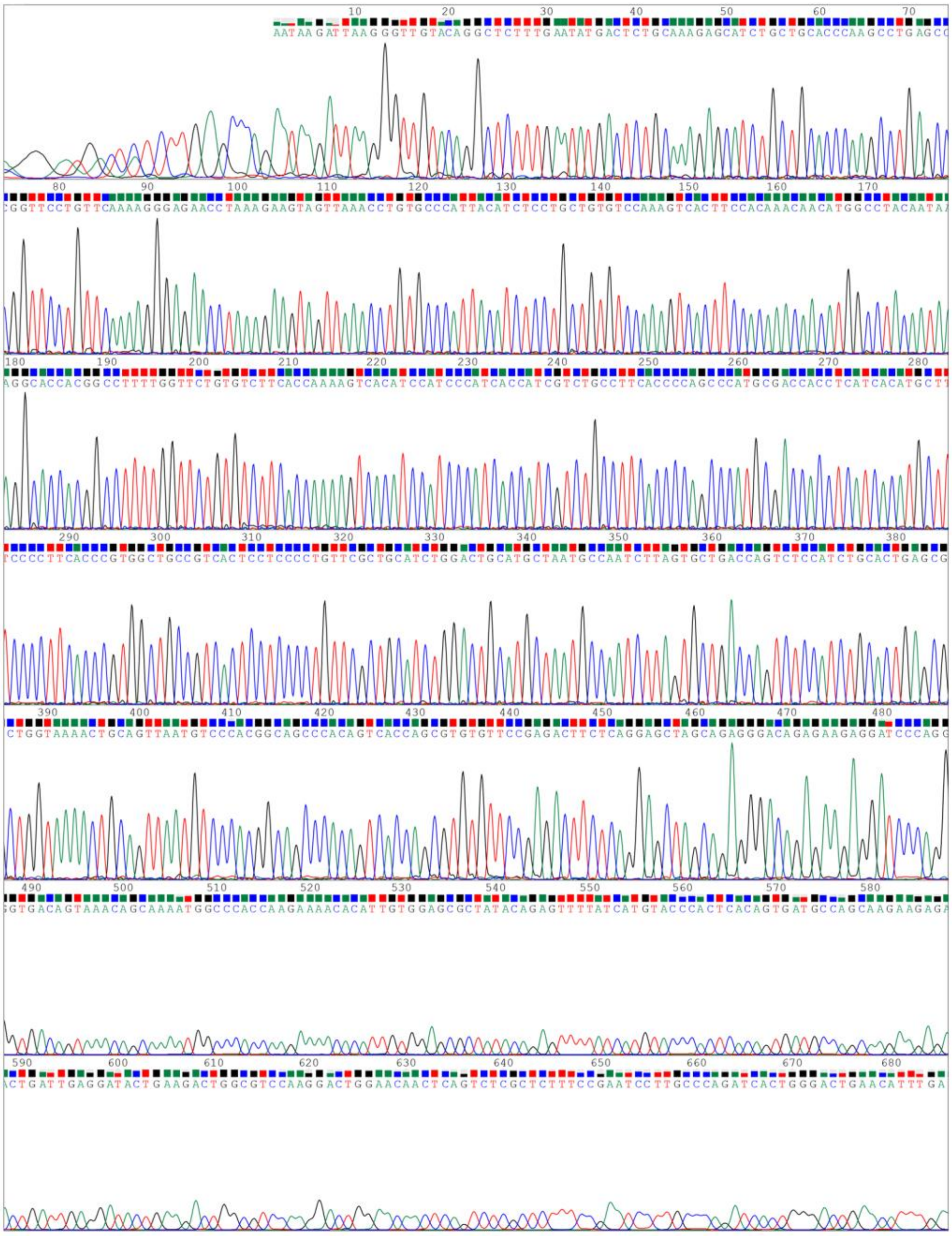

D

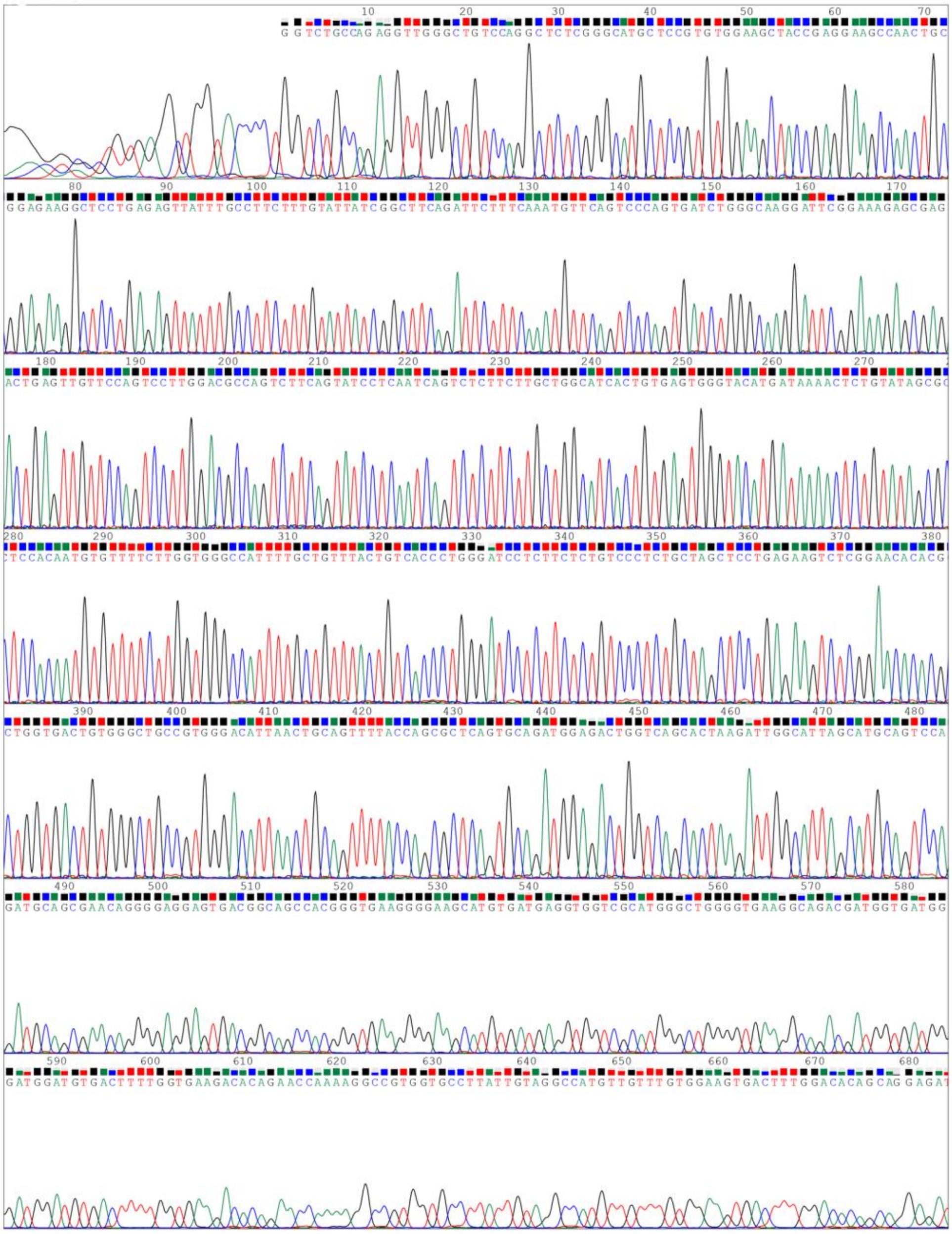

Supplemental Figure 2. The rolling circle reverse transcription and Sanger sequencing are performed to detect the full length sequence of circPDLIM5 in PC3 cells. The entire sequence of circPDLIM5 was confirmed to be identical to that from circBase database. The results of rolling circle reverse transcription and Sanger sequencing from primer of RT-789F **(A)**, RT-157R **(B)**, RT-48 F**(C)**, RT-972 R **(D)** were shown respectively.

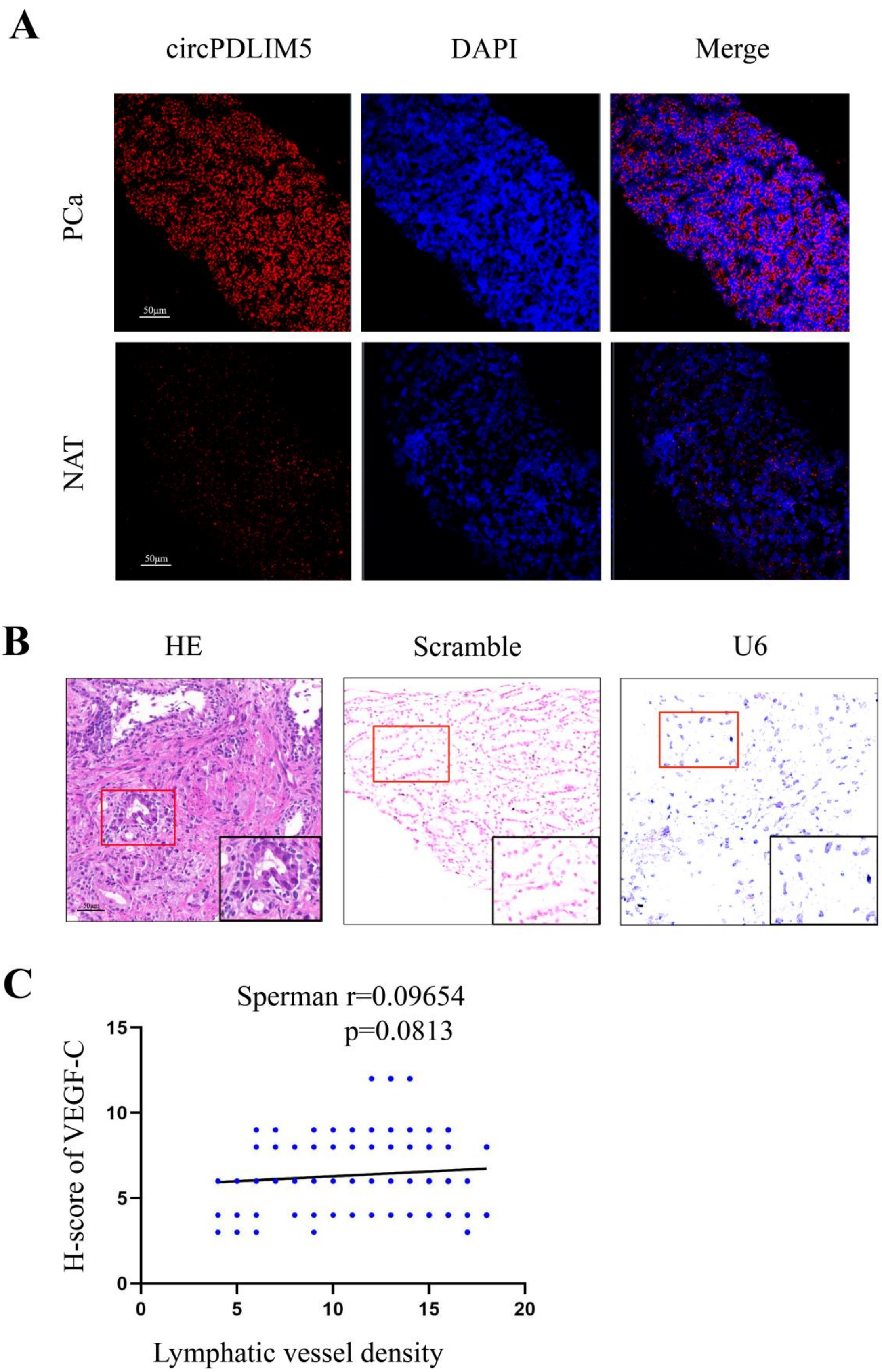

Supplemental Figure 3. CircPDLIM5 is up-regulated in PCa tissues. (A) Representative images of the circPDLIM5 expression in PCa/NAT tissues via FISH.

Scale bars: 50μm. **(B)** Representative HE or ISH images from scramble probe (red: negative control) and U6 probe (blue: positive control) in PCa tissues. The black rectangle inset is a magnification of the red rectangle area. Scale bar: 50μm. **(C)** The Pearson correlation analysis of LYVE-1 and VEGF-C.

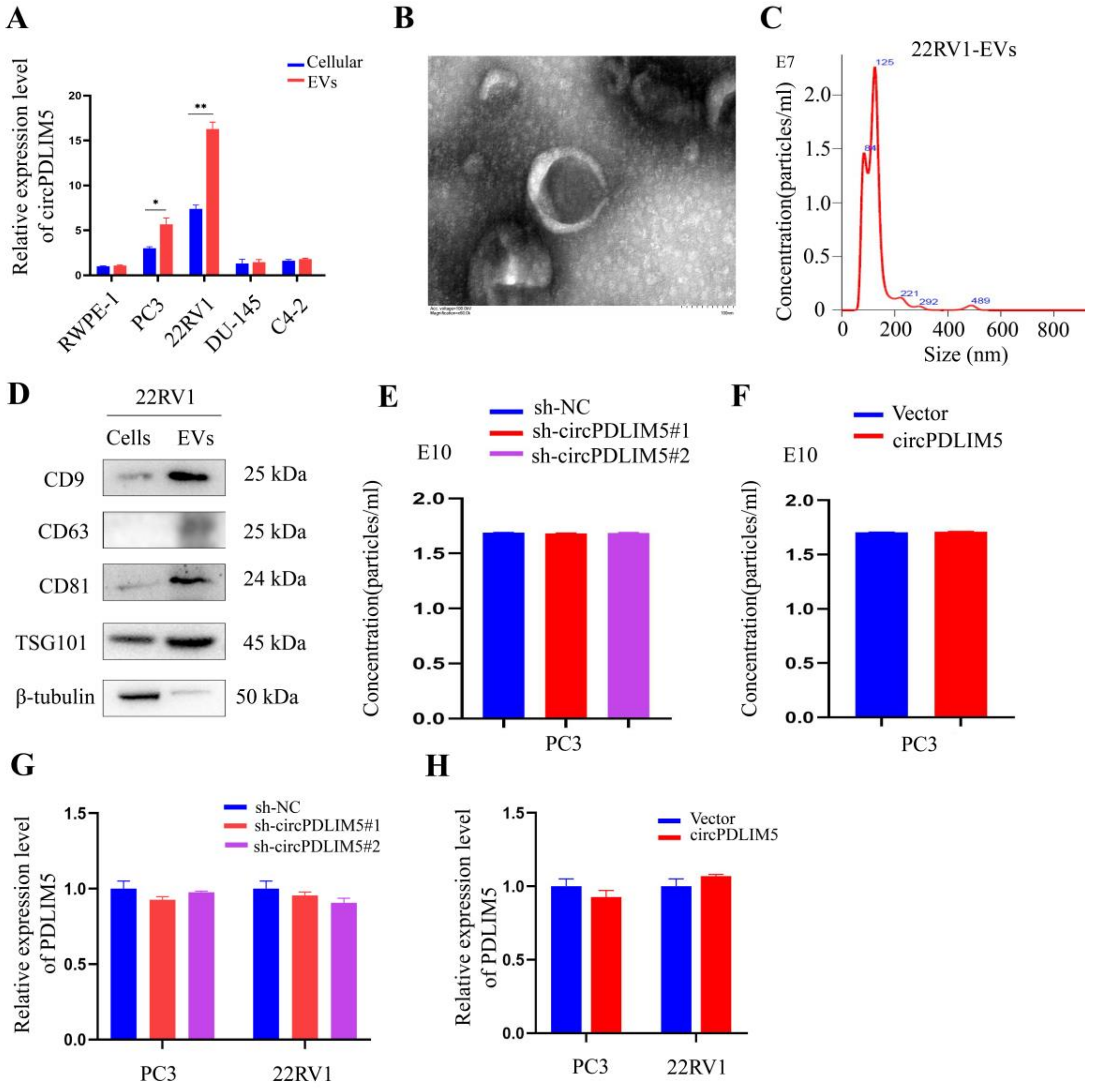

Supplemental Figure 4. circPDLIM5 is encapsulated in the PCa cell-derived EVs. **(A)** qRT-PCR analysis of circPDLIM5 expression levels in 4 human **PCa** cell lines and one human prostate epithelial cell line (RWPE-1) and their corresponding EVs. **(B** and **C)** TEM and NanoSight were used to identify the character of EVs purified from 22RV1 cells. Scale bars: 100 nm. **(D)** Western blot analysis of EVs-markers from 22RV1 EVs or cell lysates. **(E and F)** The quantity of EVs after knockdown and overexpression of circPDLIM5 in PC3 cells. qRT-PCR analysis of PDLIM5 mRNA expression after downregulation of circPDLIM5 **(G)** and overexpression of circPDLIM5(**H**). Error bars represent the standard deviation (SD) of three independent experiments. *P<0.05; **P<0.01. Statistical significance was assessed using 1-way ANOVA followed by Dunnett's tests **(A, E** and **G)**, 2-tailed Student's t test **(F and H).**

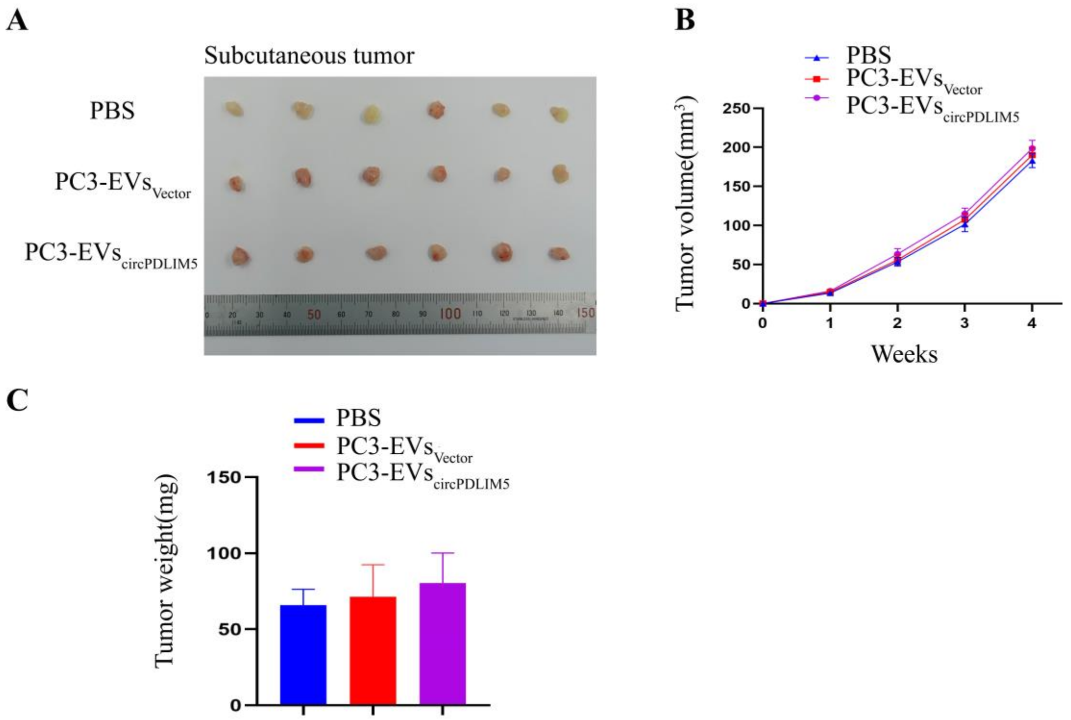

Supplemental Figure 5.EVs-circPDLIM5 is not associated with the tumorigenicity in vivo. **(A)** Subcutaneous xenograft tumors formed by PC3 cells were dissected and photographed after treated with PBS, PC3-EVs$_{\text{Vector}}$, or PC3-EVs$_{\text{circPDLIM5}}$ (n=6 for each group). Representative image of the volume of the tumors **(B)** and the weight of the tumors **(C)** in all groups (n=6 for each group). Statistical significance was assessed using 1-way ANOVA followed by Dunnett's tests **(B** and **C)**.

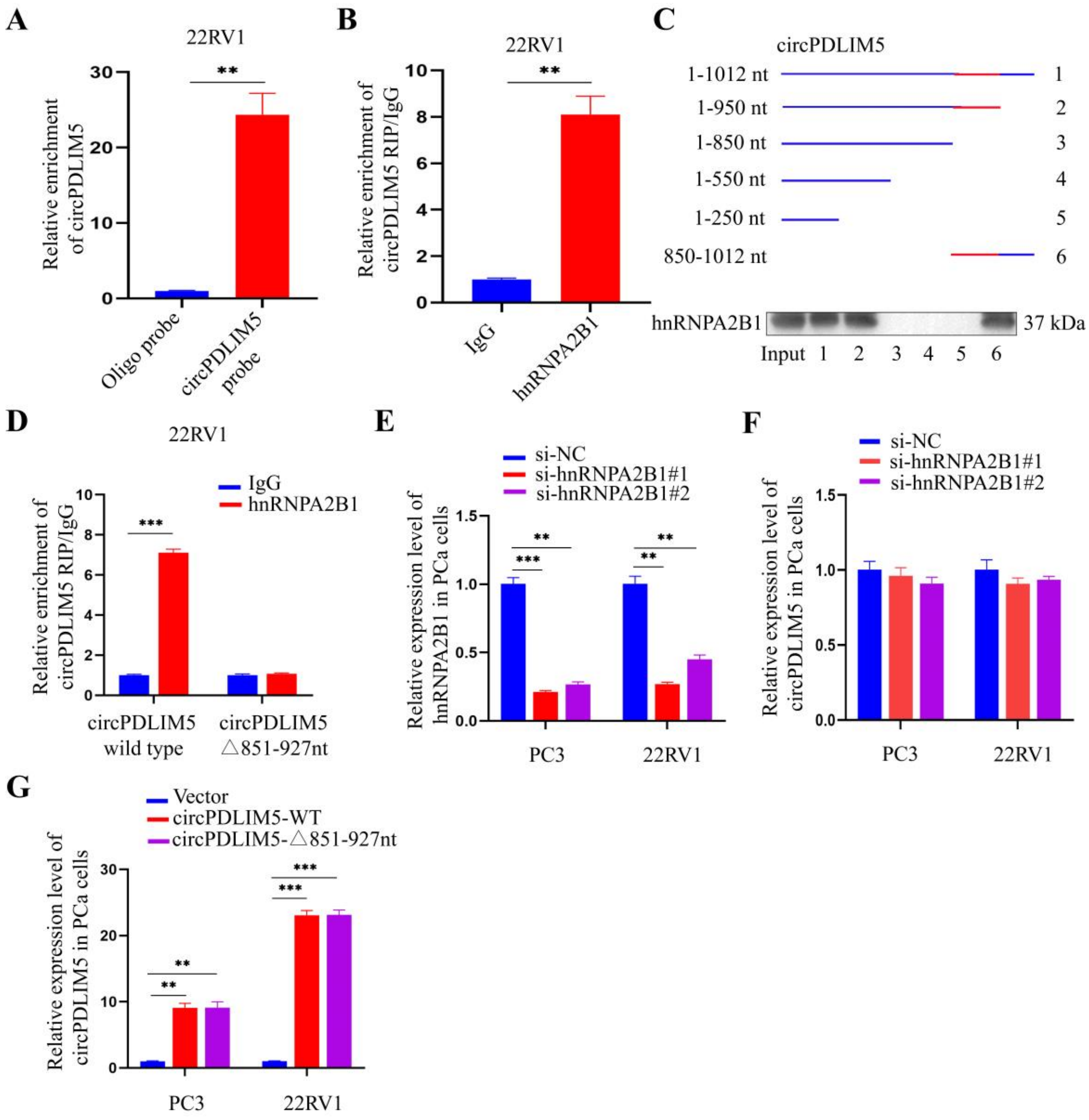

Supplemental Figure 6.The sorting of circPDLIM5 into EVs is mediated by hnRNPA2B1. (A) qRT-PCR analysis validated the circPDLIM5 probe could enrich circPDLIM5 through RNA pull-down assay in 22RV1 cells. **(B)** The enrichment of circPDLIM5 through RIP assay by anti-hnRNPA2B1 antibody in 22RV1 cells. IgG was used as a negative control. **(C)** Serial deletions of circPDLIM5 were used in RNA pull-down assays to confirm the interaction regions of circPDLIM5 to hnRNPA2B1. **(D)** RIP assay was used to validate the binding site after mutating the 851-927 nt region of circPDLIM5 in 22RV1 cells. **(E)** qRT-PCR analysis for the expression level of hnRNPA2B1 when knockdown of hnRNPA2B1 in PCa cells. **(F)** qRT-PCR analysis for the expression level of circPDLIM5 when knockdown of hnRNPA2B1 in PCa cells. **(G)** qRT-PCR analysis of circPDLIM5 in PCa cells when mutating the binding sites of circPDLIM5. Error bars represent the standard deviation (SD) of three

independent experiments. *P<0.05; **P<0.01;***P<0.001. Statistical significance was assessed using 2-tailed Student's t test **(A, B and D),** 1-way ANOVA followed by Dunnett's tests **(E-G)**.

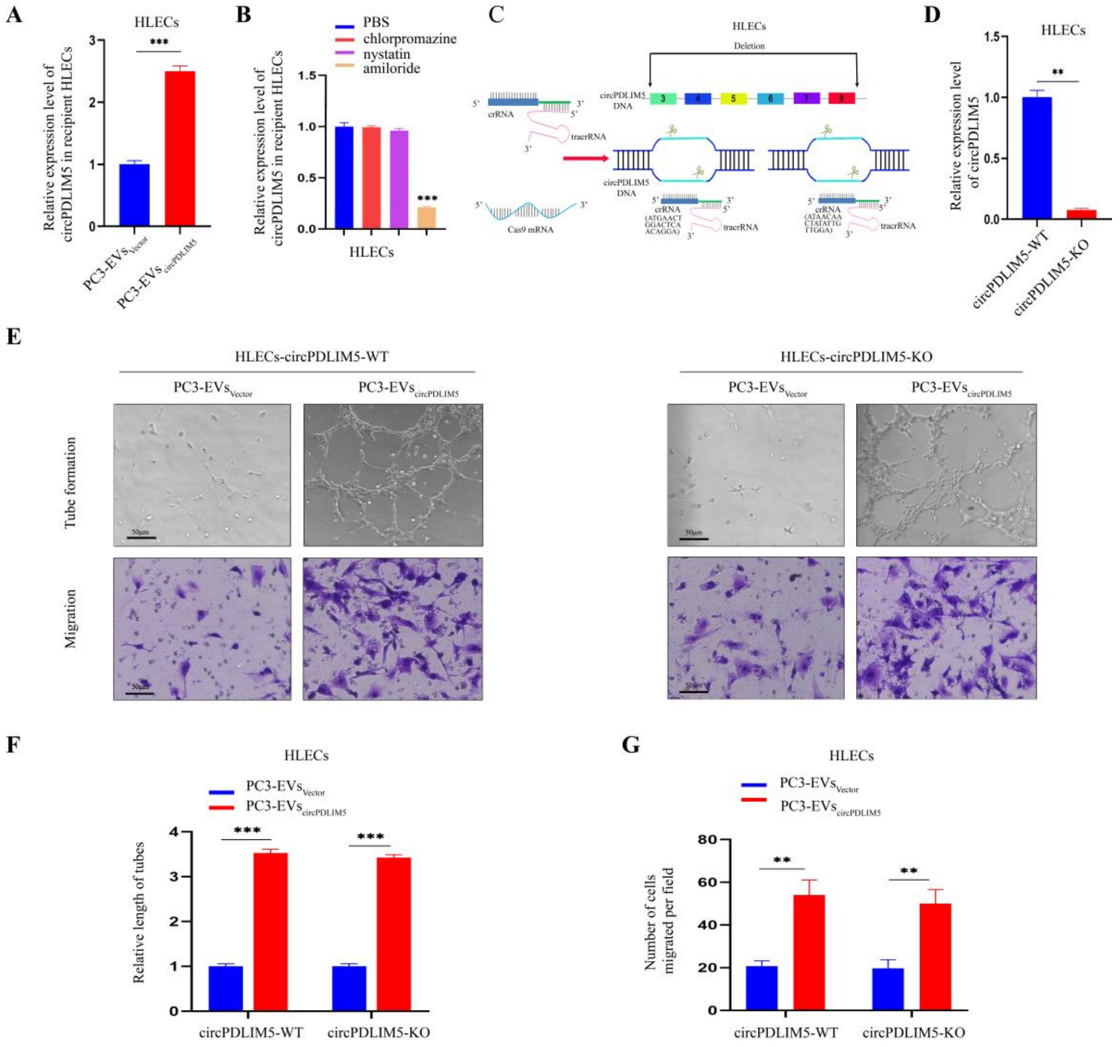

Supplemental Figure 7. The EVs derived from PCa cells which induce lymphangiogenesis are independent of the endogenous circPDLIM5 in HLECs. **(A)** The expression level of circPDLIM5 in HLECs when incubated with PC3-EVs$_{Vector}$ and PC3-EVs$_{circPDLIM5}$ through qRT-PCR. **(B)** The expression level of circPDLIM5 in HLECs when incubated with PC3-EVs and treated with chlorpromazine, nystatin, and amiloride through qRT-PCR. **(C)** The schematic model showed the establishment of HLECs-circPDLIM5-KO through CRISPR/Cas9. **(D)** qRT-PCR confirmed the knockout efficiency of circPDLIM5 in HLECs. **(E-G)** Representative images of HLECs (circPDLIM5-WT or circPDLIM5-KO) cultured with PC3-EVs$_{Vector}$ and PC3-EVs$_{circPDLIM5}$. Scale bars: 50μm. **(F)** The length of the formation tubes and **(G)** the number of Transwell migration cells. Error bars represent the standard deviation (SD) of three independent experiments. **P<0.01; ***P<0.001. Statistical significance was assessed using 2-tailed Student's t test **(A, B, C, E** and **F)**.

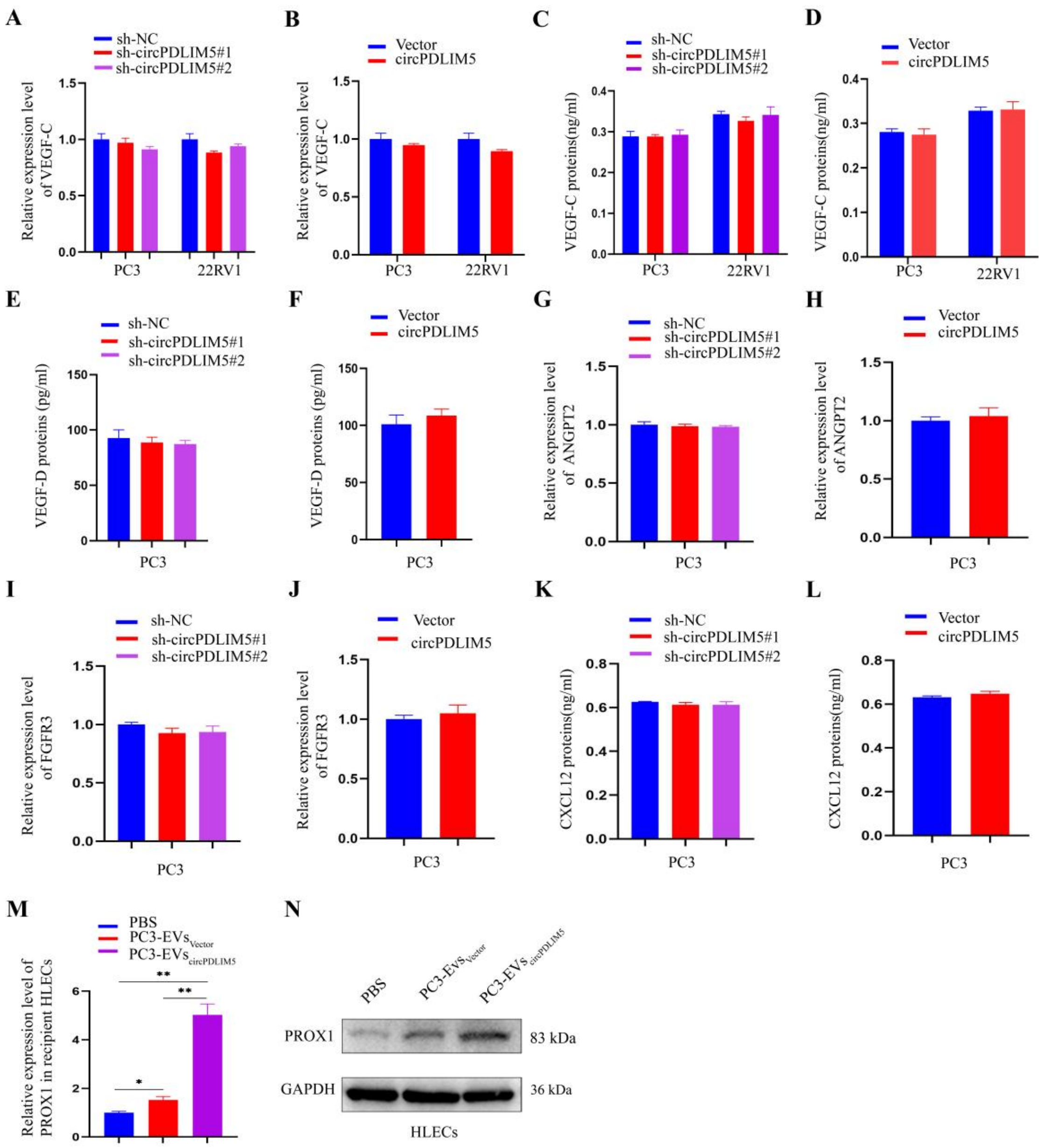

Supplemental Figure 8. EVs-circPDLIM5 promotes PROX1 expression in HLECs independently of VEGF-C and VEGF-D. (A and B) qRT-PCR analysis for the expression level of VEGF-C when knockdown or overexpress of circPDLIM5 in PCa cells. **(C and D)** The detection of VEGF-C protein level when knockdown or overexpress of circPDLIM5 in PCa cells by ELISA. (**E** and **F**) The detection of VEGF-D protein level when knockdown or overexpress circPDLIM5 in PC3 cells by ELISA. **(G and H)** qRT-PCR analysis for the expression level of ANGPT2 when knockdown or overexpress of circPDLIM5 in PC3 cells. **(I and J)** qRT-PCR analysis for the expression level of FGFR3 when knockdown or overexpress of circPDLIM5 in PC3 cells. **(K and L)** The detection of CXCL12 protein level when knockdown or overexpress of circPDLIM5 in PC3 cells by ELISA. The expression level of PROX1

was measured by qRT-PCR **(M)** and Western blot **(N)** in HLECs when cultured with PBS, PC3-EVs$_{Vector}$ and PC3-EVs$_{circPDLIM5}$. Error bars represent the standard deviation (SD) of three independent experiments. *P<0.05; **P<0.01. Statistical significance was assessed using 1-way ANOVA followed by Dunnett's tests **(A**, **C, E, G, I, K** and **M)**, 2-tailed Student's t test **(B, D, F, H, J,** and **L)**.

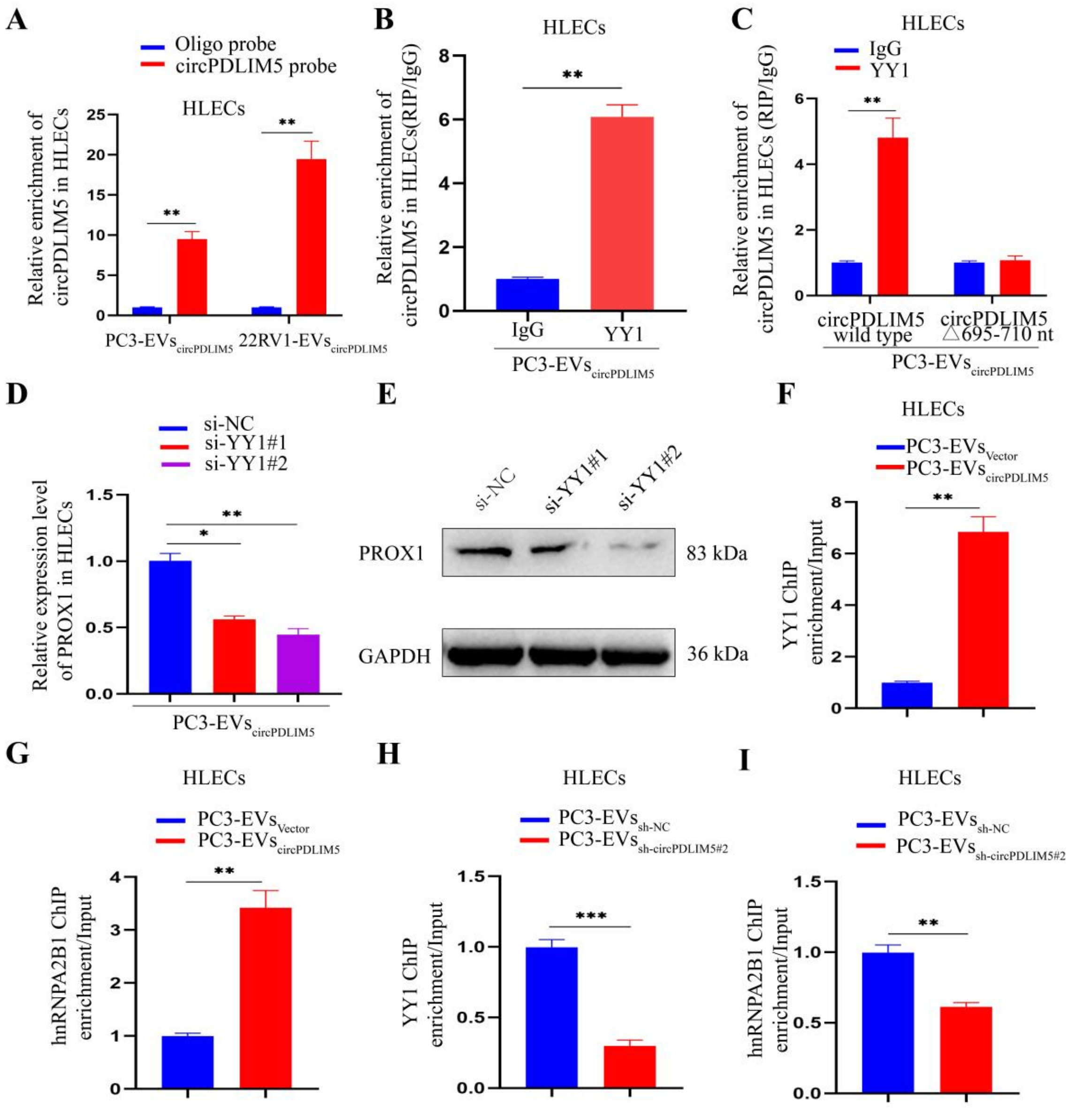

Supplemental Figure 9. EVs-circPDLIM5 up-regulates PROX1 expression by recruiting YY1 to the promoter of PROX1. (A) qRT-PCR analysis confirmed that circPDLIM5 probe could enrich circPDLIM5 through RNA pull-down assay in HLECs incubated with PC3-EVs$_{circPDLIM5}$ and 22RV1-EVs$_{circPDLIM5}$. **(B)** RIP assay through anti-YY1 antibody in HLECs treated with PC3-EVs$_{circPDLIM5}$. IgG was used as a negative control. **(C)** RIP assay was used to validate the binding sites of circPDLIM5 to YY1 after mutating the 695-710 nt region of circPDLIM5 in HLECs treated with PC3-EVs$_{circPDLIM5.}$ qRT-PCR analysis **(D)** and Western blot assay **(E)** showed the expression level of PROX1 in HLECs cocultured with PC3-EVs$_{circPDLIM5}$ after transfected with si-YY1. ChIP-qPCR of YY1 **(F** and **H)** and hnRNPA2B1 **(G** and **I)** revealed the enrichment of PROX1 promoter incubated with relevant EVs in

HLECs. Error bars represent the standard deviation (SD) of three independent experiments. *P<0.05; **P<0.01;***P<0.001. Statistical significance was assessed using 2-tailed Student's t test **(A-C** and **F-I)**, 1-way ANOVA followed by Dunnett's tests **(D)**.

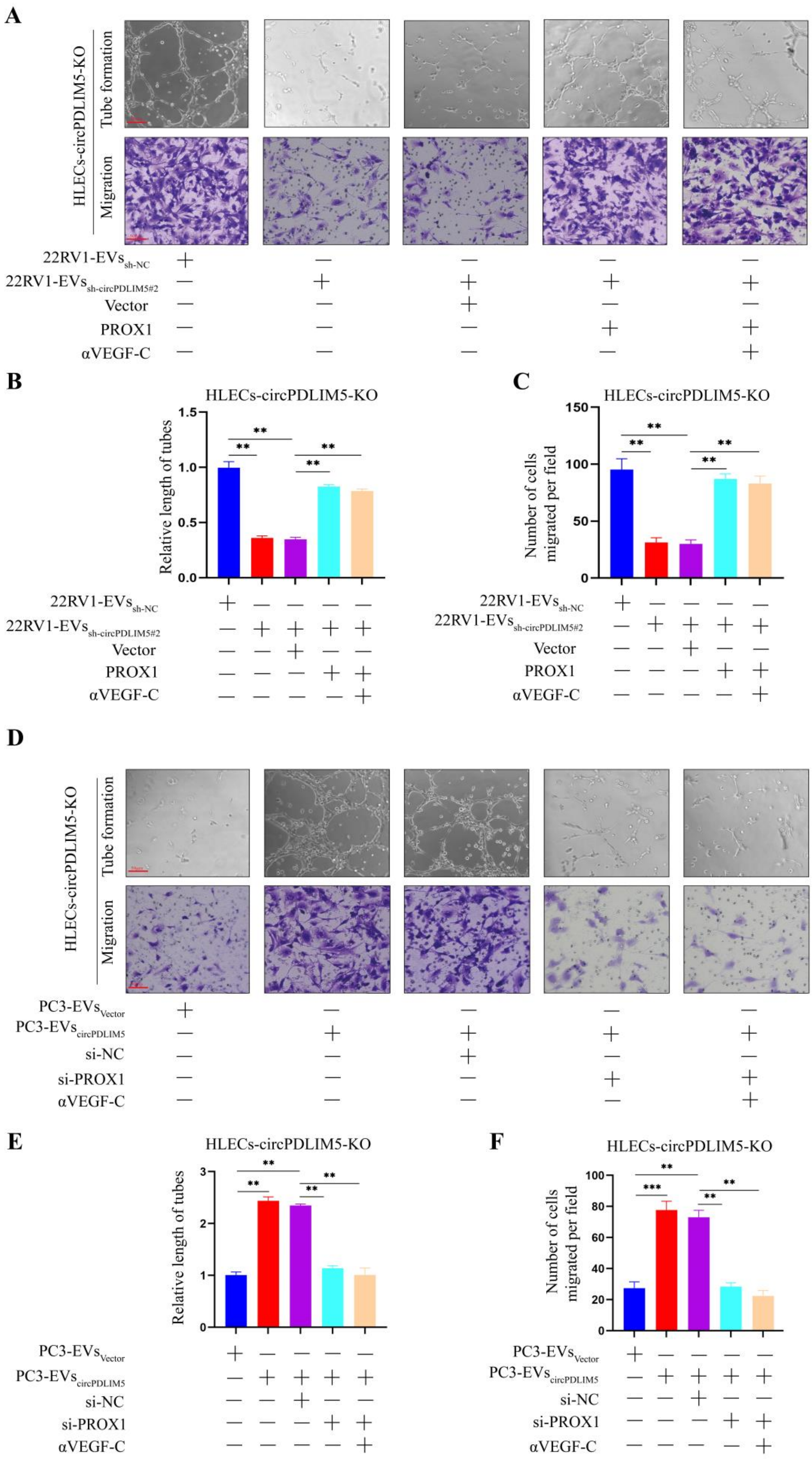
A
HLECs-circPDLIM5-KO
Tube formation
Migration
22RV1-EVs sh-NC
22RV1-EVs sh-circPDLIM5#2
Vector
PROX1
αVEGF-C
B
HLECs-circPDLIM5-KO
Relative length of tubes
C
HLECs-circPDLIM5-KO
Number of cells migrated per field
D
HLECs-circPDLIM5-KO
Tube formation
Migration
PC3-EVs Vector
PC3-EVs circPDLIM5
si-NC
si-PROX1
αVEGF-C
E
HLECs-circPDLIM5-KO
Relative length of tubes
F
HLECs-circPDLIM5-KO
Number of cells migrated per field

Supplemental Figure 10. EVs-circPDLIM5 derived from PCa cells induces lymphangiogenesis by up-regulation of PROX1 in HLECs. (A-C) Representative images of HLECs (circPDLIM5-KO) cocultured with 22RV1-EVs$_{sh\text{-}NC}$ and 22RV1-EVs$_{sh\text{-}circPDLIM5\#2}$, followed by treatment with vector, PROX1 and αVEGF-C, respectively. Scale bars: 50μm. **(B)** The length of the formation tubes and **(C)** the number of Transwell migration cells. **(D-F)** Representative images of HLECs (circPDLIM5-KO) cocultured with PC3-EVs$_{Vector}$ and PC3-EVs$_{circPDLIM5}$, followed by treatment with si-NC, si-PROX1 and αVEGF-C, respectively. Scale bars: 50μm. **(E)** The length of the formation tubes and **(F)** the number of Transwell migration cells. Error bars represent the standard deviation (SD) of three independent experiments. **P<0.01;***P<0.001. Statistical significance was assessed using 1-way ANOVA followed by Dunnett's tests **(B, C, E** and **F)**.

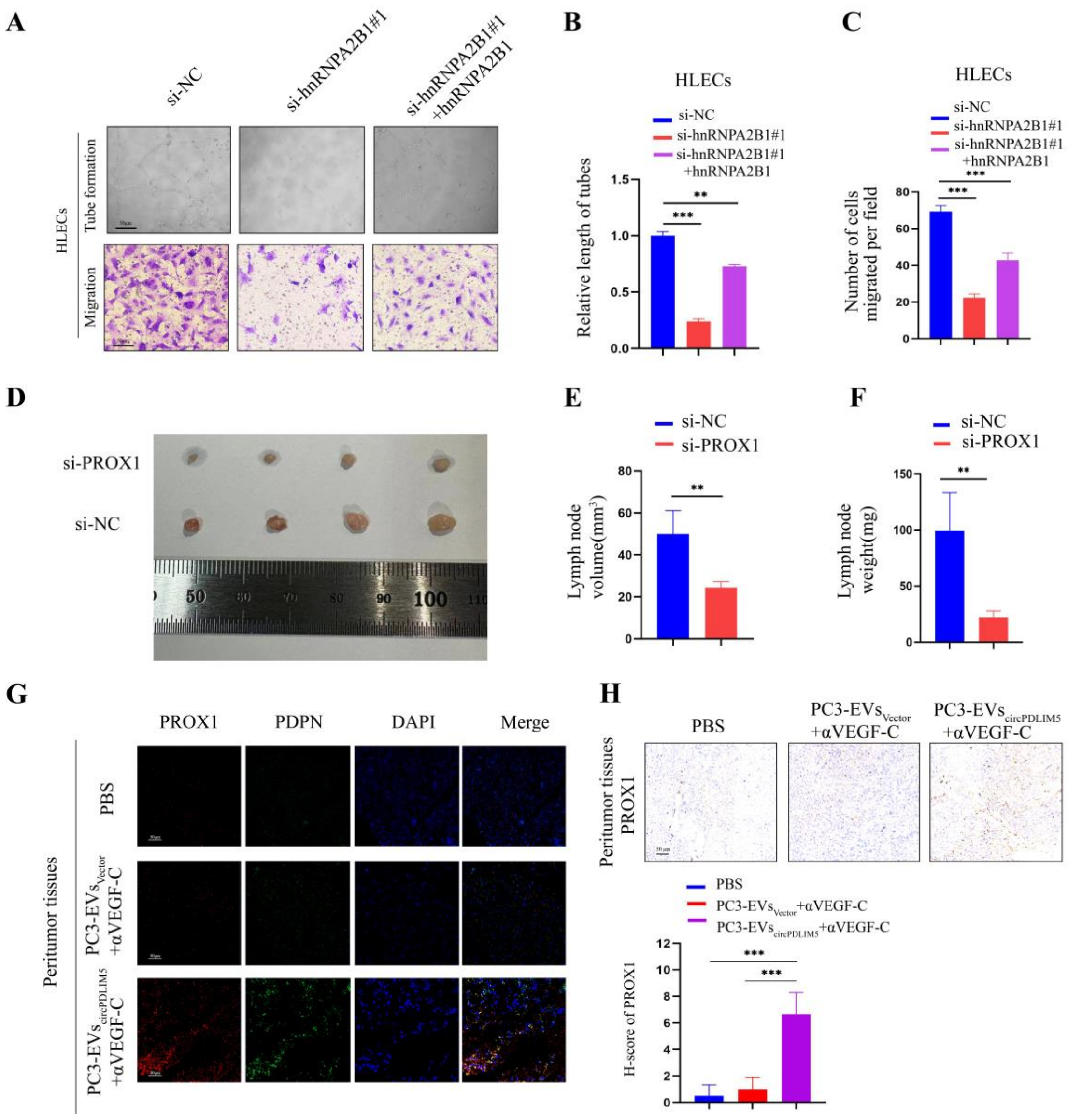

Supplemental Figure 11. hnRNPA2B1 and PROX1 were crucial for the LN metastasis of PCa. (A-C) Representative images of HLECs cocultured with EVs derived from PC3 cells treated with si-NC, si-hnRNPA2B1#1, and si-hnRNPA2B1#1+hnRNPA2B1. Scale bars: 50μm. **(B)** The length of the formation tubes and **(C)** the number of Transwell migration cells. **(D)** Representative images of dissected popliteal lymph nodes from nude mice with tail vein injected with si-NC and si-PROX1 after inoculation with PC3 cells and PC3-EVs into the footpad (n=4 per group). Representative images of the volume of lymph nodes **(E)**, and the weight of lymph nodes **(F)** in all groups. **(G)** Representative images for the expression of PROX1 and PDPN in peritumor tissues via IF. (**H**) Representative images of IHC staining by anti-PROX1 anti-body and the analysis of H-score of PROX1 in peritumor tissues (n=10 per group). Scale bars: 50μm. Statistical significance was assessed using

1-way ANOVA followed by Dunnett's tests (**B, C,** and **H**), 2-tailed Student's t test (**E** and **F**).

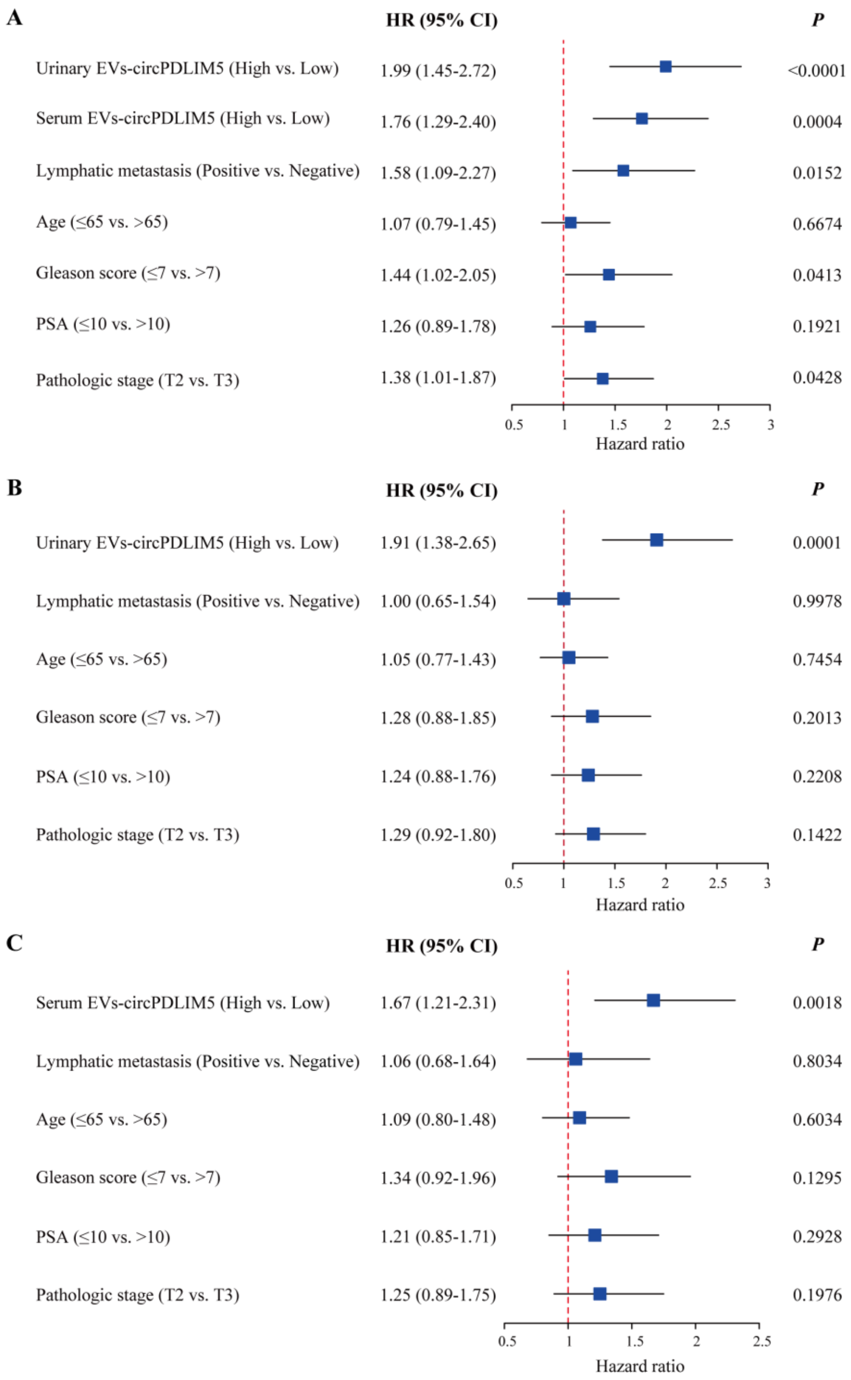

Supplemental Figure 12. Univariate and multivariate analysis of progression-free survival for EVs-circPDLIM5 expression in patients with PCa (n=327). (A) Univariate analysis of progression-free survival for urinary/serum EVs-circPDLIM5 expression in PCa patients (n=327). Multivariate analysis of progression-free survival for urinary EVs-circPDLIM5 expression **(B)** and serum EVs-circPDLIM5expression **(C)** in PCa patients (n=327).

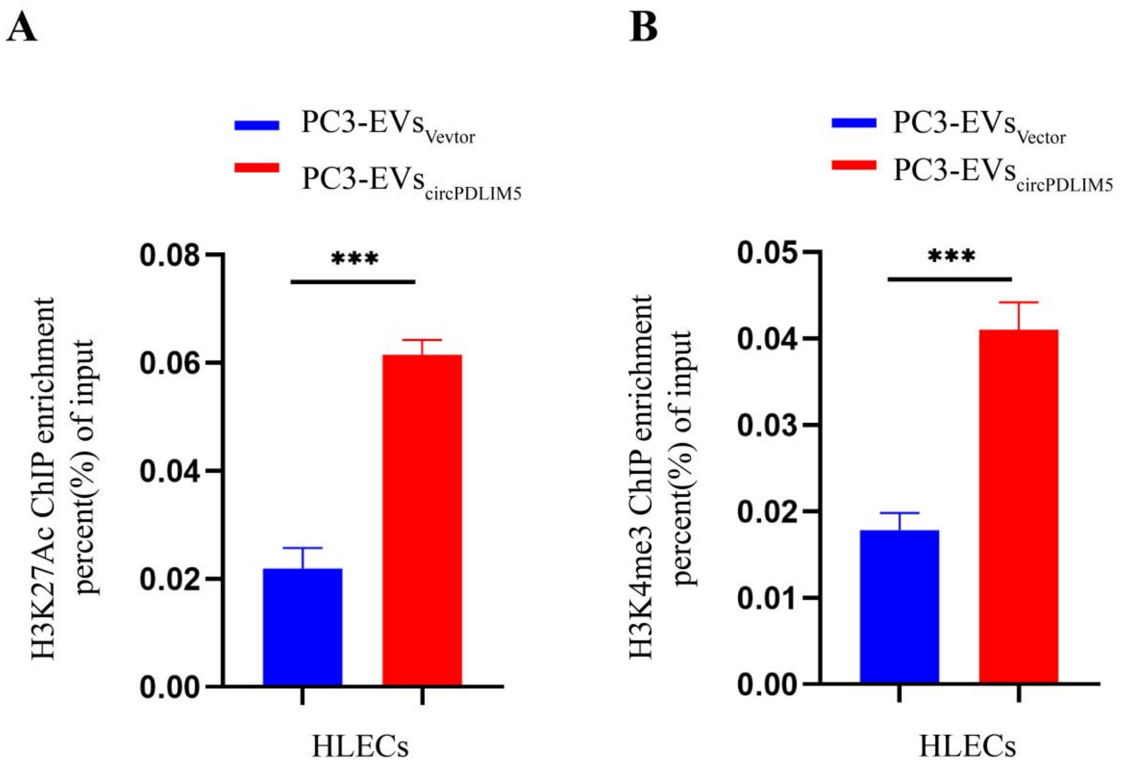

Supplemental Figure 13. Histone modifications play a significant role in the circPDLIM5/YY1 mediated lymph-node metastasis of PCa. ChIP-qPCR analysis of H3K27Ac occupancy (**A**) and H3K4me3 status (**B**) in the PROX1 promoter in HLECs treated with PC3-EVsVector and PC3-EVscircPDLIM5. ***P<0.001.

Supplemental Tables

Supplemental Table 1. The clinicopathological characteristics of PCa patients for RNA sequencing on urinary EVs.

	PCa with LN (-) (n=5)	PCa with LN (+) (n=5)	P value
Age, yr, median, IQR	65 (59-74)	69 (66-62)	0.500†
PSA, ng/mL, median, IQR	4.32 (4.14-5.93)	6.16 (5.61-7.03)	0.151†
Family history, n			—
Yes	0	0	
No	5	5	
Grade Group, n			—
GG 1 (ISUP1, GS3 + 3)	0	0	
GG 2 (ISUP2, GS3 + 4)	1	1	
GG 3 (ISUP3, GS4 + 3)	1	1	
GG 4 (ISUP4, GS8)	2	2	
GG 5 (ISUP5, >GS8)	1	1	

PCa=prostate cancer; EV=extracellular vesicles; LN (+) = lymph node positive; LN (-) =lymph node negative; IQR=interquartile range; PSA=prostate-specific antigen; GG=Grade Group; ISUP=International Society of Urological Pathology; GS=Gleason score. †Mann-Whitney U test.

Supplemental Table 2. Four circRNAs consistently upregulated in the urinary EVs from both high-grade and LN-positive PCa patients

CircRNA ID	Chromesome	Location
hsa_circ_0004028	4	chr4:95444874-95539342
hsa_circ_0127850	5	chr5:128861976-128932357
hsa_circ_0017924	10	chr10:20432223-20453496
hsa_circ_0127852	5	chr5:129030412-129040084

EV=extracellular vesicles; LN = lymph node positive; PCa=prostate cancer.

Supplemental Table 3. The correlation between EVs-circPDLIM5 expression and clinicopathologic characteristics of 327 PCa patients.

Characteristics	Patients(n)	Urinary EVs-circPDLIM5 expression			Serum EVs-circPDLIM5 expression			Tissue EVs-circPDLIM5 expression		
		low	high	P value	low	high	P value	low	high	P value
Age(y), No. (%)				0.348			0.549			0.3918
≤65	174	92(52.9%)	82(47.1%)		91(52.3%)	83(47.7%)		89(51.1%)	85(48.9%)	
>65	153	72(47.1%)	81(52.9%)		74(48.4%)	79(51.6%)		71(46.4%)	82(53.6%)	
PSA (ng/mL), No. (%)				0.649			0.262			0.7392
≤10	93	49(52.7%)	44(47.3%)		52(56.0%)	41(44.0%)		48(51.6%)	45(48.4%)	
>10	234	115(49.1%)	119(50.9%)		113(48.3%)	121(51.7%)		116(49.6%)	118(50.4%)	
Gleason score, No. (%)				0.075			0.447			0.1685
≤7	257	136(52.9%)	121(47.1%)		133(51.8%)	124(48.2%)		134(52.1%)	123(47.9%)	
>7	70	28(40%)	42(60%)		32(45.7%)	38(54.3%)		30(42.9%)	40(57.1%)	
Pathologic stage, No. (%)				0.106			0.087			0.1222
T2	192	104(54.2%)	88(45.8%)		105(54.7%)	87(45.3%)		102(53.1%)	90(46.9%)	
T3	135	60(44.4%)	75(55.6%)		60(44.4%)	75(55.6%)		60(44.4%)	75(55.6%)	
Lymphatic metastasis, No. (%)				<0.0001			<0.0001			<0.0001
Negative	265	151(57%)	114(43%)		151(57%)	114(43%)		151(57%)	114(43%)	
Positive	62	13(21%)	49(79%)		14(22.6%)	48(77.4%)		11(18.3%)	51(81.7%)	

EV=extracellular vesicles; PCa=prostate cancer; PSA=prostate-specific antigen.

Supplemental Table 4. The assessment of LN status by Urinary EVs-circPDLIM5 expression compared with MRI in PCa patients.

Pathological LN status	Urinary EVs-circPDLIM5-reported LN (+)*			Urinary EVs-circPDLIM5-reported LN (-)			Total
	MRI-reported LN (+)	MRI-reported LN (-)	Total	MRI-reported LN (+)	MRI-reported LN (-)	Total	
LN (+)	8	32	40	9	13	22	62
LN (-)	2	39	41	10	214	224	265
Total	10	71	81	19	227	246	327

EV=extracellular vesicles; PCa=prostate cancer; LN (+) = lymph node positive; LN (-) = lymph node negative. * The best cut-off of urinary EVs-circPDLIM5 was used to distinguish LN-positive and LN-negative PCa.

Supplemental Table 5. The sh-RNAs, si-RNAs and CRISPR-Cas9 used in this study

sh-RNAs or si-RNAs	Sequence(5'-3')
sh-NC(circPDLIM5)	TTCTCCGAACGTGTCACGT
sh-circPDLIM5#1	GGCCAAACCAAGGAGCTAAAA
sh-circPDLIM5#2	CAAACCAAGGAGCTAAAAGAT
si-hnRNPA2B1#1	GGAGAGTAGTTGAGCCAAA
si-hnRNPA2B1#2	GCTACGGAGGTGGTTATGA
si-YY1#1	GCCUCUCCUUUGUAUAUUAUUTT
si-YY1#2	GACGACGACUACAUUGAACAATT
CRISPR-Cas9-circPDLIM5-crRNA (upstream)	ATGAACTGGACTCAACAGGA
CRISPR-Cas9-circPDLIM5-crRNA (downstream)	ATAACAACTATATTGTTGGA
si-PROX1	GGCTCTCCTTGTCGCTCATAA

Supplemental Table 6. Primes used in qRT-PCR and PCR analysis in this study

Primers	Sequence(5’-3’)
GAPDH	F: CAGGAGGCATTGCTGATGAT<br>R: GAAGGCTGGGGCTCATTT
U6	RT:GTCGTATCCAGTGCAGGGTCCGAGGTATTCGCACTGGA TACGACAAAATA<br>F: CTCGCTTCGGCAGCACA<br>R: AACGCTTCACGAATTTGCGT
circPDLIM5	F: GTTACCAGCCTCACAGCTGC<br>R: GCCGCCATCTTTTAGCTCCT
hsa_circ_0127850	F:CTTGAGTGGAATGTGTAGTG<br>R:GTCTTCCTTTATTTGTGACC
hsa_circ_0017924	F: CCAACAAATTCCCAGTTTCA<br>R: CCACAAGTGCTGTGCCATTA
hsa_circ_0127852	F: GGTGACTGGTCTAAGGAGAA<br>R: GGCTCTGGCTTGGTTAAGTA
PDLIM5	F: CGGACCCGAGCATATTTCATT<br>R:ACTAGAGATTGTCAGAGGCATGTTG
circPDLIM5 (divergent)	F: GTTACCAGCCTCACAGCTGC<br>R: GCCGCCATCTTTTAGCTCCT
circPDLIM5 (convergent)	F: TAACTCTCAGGAGCCTTCTCCG<br>R: CGTTGCCAGCTTGGTGACTT
GAPDH (convergent)	F: CAGGAGGCATTGCTGATGAT<br>R: GAAGGCTGGGGCTCATTT
Rolling circle amplification (circPDLIM5)	RT-789F: GAAAGAATCTGAAGCCGATAATACA<br>RT-157R: ATGCTCTTTGCAGAGTCATATTCAA<br>RT-48 F: TGTGGTTCTCAGCATTGATGGA<br>RT-972 R: GCCAGTGGATCCTACAGGCTT
miR-39-3p	RT:GTCGTATCCAGTGCAGGGTCCGAGGTATTCGCACTGGA

	TACGACTCCACA
	F: ACACCATCACCGGGTGTAAAT
	R: AGTGCAGGGTCCGAGGTATT
hnRNPA2B1	F: GGTGCTCCTCGCAGAGTTGT
	R: TCGCTTCAGCCCGATTTC
VEGF-C	F: GAGCAGTTACGGTCTGTGTCCA
	R: CTTCTGTCCTTGAGTTGAGGTTG
ANGPT2	F: CAATTATTCAGCGACGTGAG
	R: AAGGGTTACCAAATCCCAC
FGFR3	F: CCTCGGGAGATGACGAAGC
	R: CGGGCCGTGTCCAGTAAGG
PROX1	F: CAGCCCGAAAAGAACAGAAG
	R: GGGTCTAGCTCGCACATCTC
PROX1(P1)	F: AAACAGGGAGGGTGAACGGG
	R: AGCCAGCTGGACTGTCATGG
PROX1(P2)	F: ACACAGCACTCATTTGTGGGC
	R: TGTGGAGTGGTGCTTCCGAA
PROX1(P3)	F: CGTCAGGCAGGCACCTACTT
	R: GCGTGCACGTTTGAAGGGAT
PROX1(P4)	F: ATCTGGCGCTCGCTCTCTCT
	R: ATCTCCTCTCCAGGCACTCG
PROX1(P5)	F: TGGCACACTGGAGGAGGTACAC
	R: GGAGTCCCTTTCGGATGTGTG
PROX1 (ChIP)	F: ATGTGAAACCTCTGGCACCT
	R: GGAGAAAAAGTGGGGGTTTT

Supplemental Table 7. Probes of FISH, RNA pull-down, ChIRP and ISH in this study

Probes	Sequence(5'-3')
circPDLIM5 FISH probe	CTTTTAGCTCCTTGGTTTGG
Biotin labeled control probe (RNA pull-down)	AGTCGGAACTGAAATCATGT
Biotin labeled circPDLIM5 probe (RNA pull-down)	CTTTTAGCTCCTTGGTTTGG
circPDLIM5 ChIRP control probe	AGTCGGAACTGAAATCATGT
circPDLIM5 ChIRP probe	CTTTTAGCTCCTTGGTTTGG
circPDLIM5 ISH probe	CTTTTAGCTCCTTGGTTTGG 5'-DIG labeled
Scramble ISH probe (negative control)	AGTCGGAACTGAAATCATGT 5'-DIG labeled
U6 ISH probe	CACGAATTTGCGTGTCATCCTT 5'-DIG labeled

FISH=Fluorescence in situ hybridization; ChIRP=Chromatin isolation by RNA purification; ISH=In situ hybridization; DIG=Digoxigenin.
