# Supplementary figures and images for: Extracellular vesicle-mediated transmission of circPDLIM5 promotes lymphatic metastasis in prostate cancer
Source: J Exp Clin Cancer Res. 2025 Jul 3;44:188. doi: 10.1186/s13046-025-03443-2 (PMC12224790; doi:10.1186/s13046-025-03443-2)

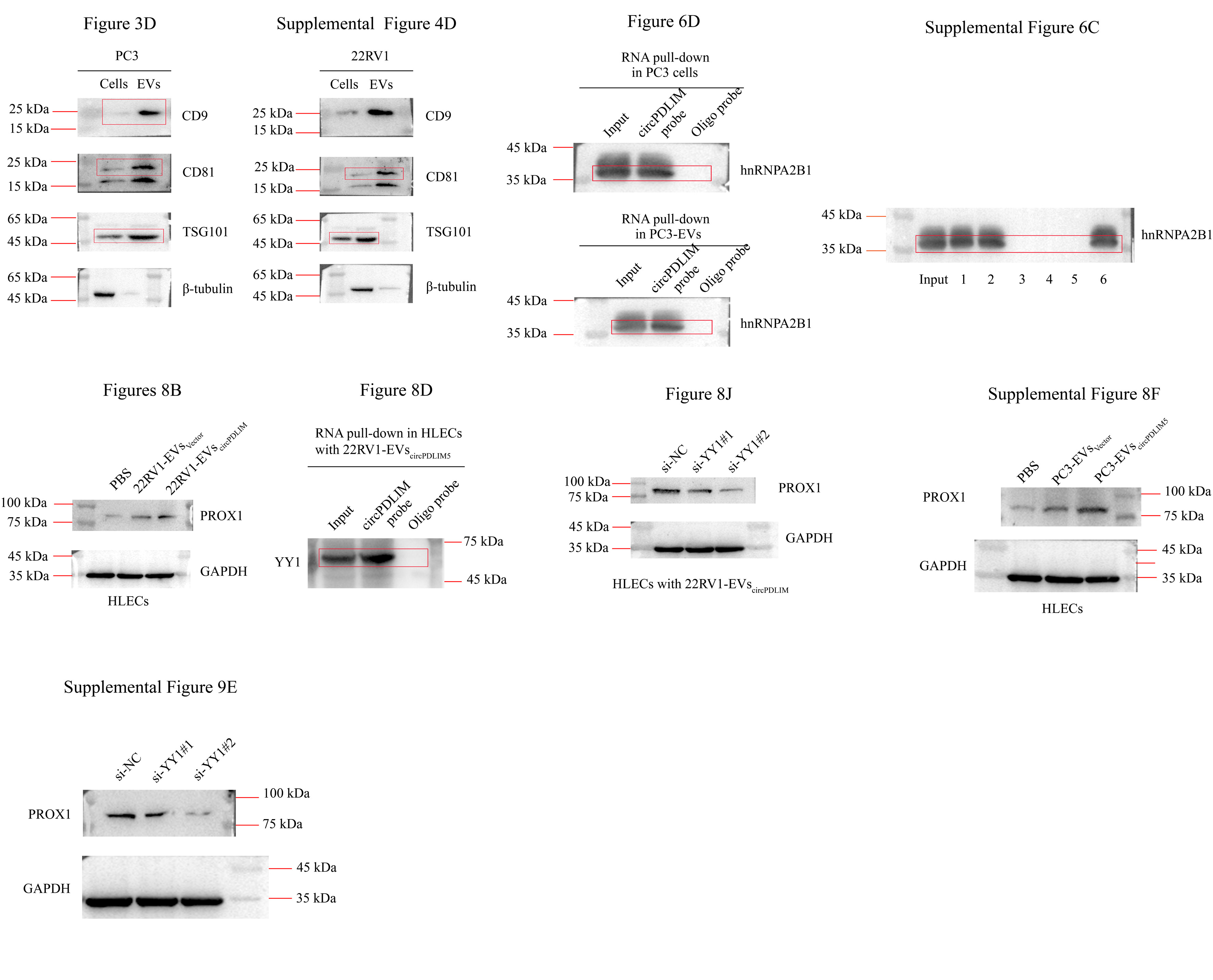

Supplement: Supplementary file 4 — Supplementary Material 4. [file 13046_2025_3443_MOESM4_ESM.jpg]
